# Navigating protein landscapes with a machine-learned transferable coarse-grained model

**DOI:** 10.1038/s41557-025-01874-0

**Published:** 2025-07-18

**Authors:** Nicholas E. Charron, Klara Bonneau, Aldo S. Pasos-Trejo, Andrea Guljas, Yaoyi Chen, Félix Musil, Jacopo Venturin, Daria Gusew, Iryna Zaporozhets, Andreas Krämer, Clark Templeton, Atharva Kelkar, Aleksander E. P. Durumeric, Simon Olsson, Adrià Pérez, Maciej Majewski, Brooke E. Husic, Ankit Patel, Gianni De Fabritiis, Frank Noé, Cecilia Clementi

**Affiliations:** 1 Department of Supercomputing, Zuse Institute Berlin, Berlin, Germany; 2 Department of Physics, Freie Universität Berlin, Berlin, Germany; 3 Department of Physics and Astronomy, Rice University, Houston, TX USA; 4 Center for Theoretical Biological Physics, Rice University, Houston, TX USA; 5 Department of Mathematics and Computer Science, Freie Universität Berlin, Berlin, Germany; 6 Department of Chemistry, Rice University, Houston, TX USA; 7 Department of Computer Science and Engineering, Chalmers University of Technology and University of Gothenburg, Gothenburg, Sweden; 8 Computational Science Laboratory, Universitat Pompeu Fabra, Barcelona, Spain; 9 Acellera Labs, Barcelona, Spain; 10 Lewis Sigler Institute for Integrative Genomics, Princeton University, Princeton, NJ USA; 11 Department of Neuroscience, Baylor College of Medicine, Houston, TX USA; 12 Department of Electrical and Computer Engineering, Rice University, Houston, TX USA; 13 Institució Catalana de Recerca i Estudis Avançats (ICREA), Barcelona, Spain; 14 AI4Science, Microsoft Research, Berlin, Germany

**Keywords:** Computational chemistry, Computational biophysics

## Abstract

The most popular and universally predictive protein simulation models employ all-atom molecular dynamics, but they come at extreme computational cost. The development of a universal, computationally efficient coarse-grained (CG) model with similar prediction performance has been a long-standing challenge. By combining recent deep-learning methods with a large and diverse training set of all-atom protein simulations, we here develop a bottom–up CG force field with chemical transferability, which can be used for extrapolative molecular dynamics on new sequences not used during model parameterization. We demonstrate that the model successfully predicts metastable states of folded, unfolded and intermediate structures, the fluctuations of intrinsically disordered proteins and relative folding free energies of protein mutants, while being several orders of magnitude faster than an all-atom model. This showcases the feasibility of a universal and computationally efficient machine-learned CG model for proteins.

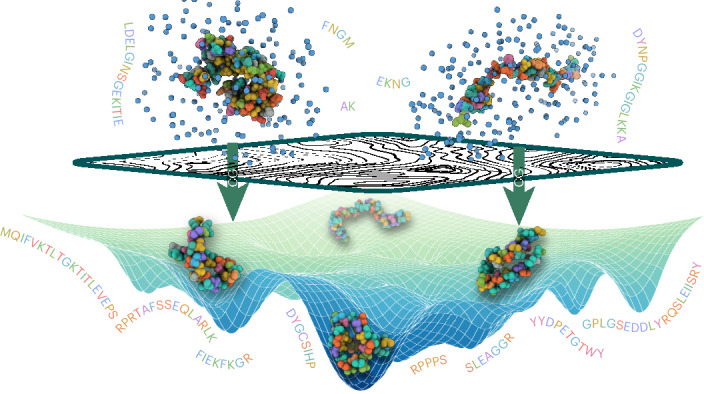

## Abstract (web summary)

The development of a universal protein coarse-grained model has been a long-standing challenge. A coarse-grained model with chemical transferability has now been developed by combining deep-learning methods with a large and diverse training set of all-atom protein simulations. The model can be used for extrapolative molecular dynamics on new sequences.

## Main

Over the past 50 years, substantial developments in hardware, software and theory have advanced the simulation of macromolecules from proof of principle to in silico study of protein folding and dynamics^1,2^. Despite this success, an ongoing challenge of the field is the accurate and efficient representation of large, biologically relevant systems. These systems are inherently multiscale: while fine-grained models must be used to describe local and fast processes, the long-timescale dynamics may be better captured at a coarse-grained (CG) resolution, which is both more computationally efficient and facilitates a more direct understanding of how macroscopic observables arise from interactions between microscopic degrees of freedom. Up to now, the most successful and widely used simulation approach is molecular dynamics (MD) with all-atom resolution^1,3^. Atomistic MD effectively models macromolecular changes, such as protein folding or protein–ligand binding, and predicts their thermodynamic properties. However, all-atom MD comes at extreme computational costs and requires great efforts to post-process and analyse the data^4,5^. It is still unclear whether there is a computationally efficient CG scale that lends itself to a general and accurate simulation model. Although deep-learning methods have been wildly successful in predicting protein structure and function by reasoning over large-scale genomic and structure datasets^6,7^, they often do not tie into a physical level of understanding. In this Article we show that deep learning can be used to develop a universal CG protein force field capable of predicting protein structures, structure transitions, folding mechanisms, folding upon binding of an intrinsically disordered peptide, and changes of free energy upon mutation, similar to all-atom MD methods, but orders of magnitude faster.

Most MD simulation studies employ atomistic force fields fitted on a combination of quantum-chemical calculations and experimental data. Modern force fields have been shown to be qualitatively accurate for processes on nanosecond to millisecond timescales and are often quantitatively consistent with experiments^2,8^. Recently introduced machine-learned force fields^9–11^ may capture the quantum-mechanical interactions between nuclei in the Born–Oppenheimer approximation even more accurately than conventional MD, but they also come at higher computational cost^12^.

Ever since the first protein simulations, the community has striven to develop universal (CG) macromolecular models that are computationally more efficient and more simple to analyse. The feasibility of such models is justified by statistical mechanical descriptions of protein dynamics, such as energy landscape theory^13^, and results from decades of analysis of atomistic simulations^4^. These studies suggest that a protein’s free energy landscape can be sufficiently described by a reduced number of collective variables with minimal loss of accuracy compared with atomistic MD. Some CG models have shown success in specific systems. These include structure-based models^14^, which rely on the known native structure of a protein to explore its free energy landscape, the Martini^15^ CG force field, which can effectively model intermolecular interactions including membrane structure formation and protein interactions, and CG force fields developed to model protein folding and conformational dynamics such as UNRES^16^ or AWSEM^17^. These models are limited to system-specific applications. For instance, Martini inaccurately models intramolecular protein dynamics, and UNRES and AWSEM often do not capture alternative metastable states.

The main hindrance to the development of an accurate biomolecular CG model is the difficulty in efficiently modelling multi-body interaction terms, which are essential to realistically represent correct protein thermodynamics and implicit solvation effects^18,19^. In contrast, classical all-atom force fields model most non-bonded interactions as a sum of two-body terms.

Bottom–up CG force fields^20^ are typically fit to match the equilibrium distribution of an all-atom model, so they could in principle reach atomistic-level accuracy and predictiveness. By leveraging recent developments in deep learning, it has become possible to machine-learn such many-body CG force fields using neural networks^18,21–30^. In particular, using the variational force-matching approach^31,32^, such force fields have been shown to accurately reproduce the all-atom distributions of CG observables for single^18,21,22,25,27,33^ and multiple proteins^24^. Despite these advancements, a transferable CG force field that could be considered as universal, quantitative, predictive and as reliable as a modern atomistic force field is still missing^34^.

In this Article we propose a neural network-based CG model that is truly transferable in sequence space. We learn the model parameters using a bottom–up approach from atomistic simulations of a set of proteins and then use it to successfully simulate the conformational dynamics of proteins never seen at any learning stage, with low (16–40%) sequence similarities to the training or validation protein set. This CG model is orders of magnitude faster than all-atom MD simulations, predicts metastable folded, unfolded and intermediate states comparable with all-atom MD simulations, and is consistent with experimental data for larger proteins, such as relative folding free energies of protein mutants, where converged all-atom simulations are not available. These results indicate that the CG model ‘learns’ to represent effective physical interactions between the CG degrees of freedom and provides strong support for the hypothesis that, using deep-learning methods, a universal CG model for realistic and predictive protein simulations at low computational cost is within reach.

## Results

**Fig. 1:**
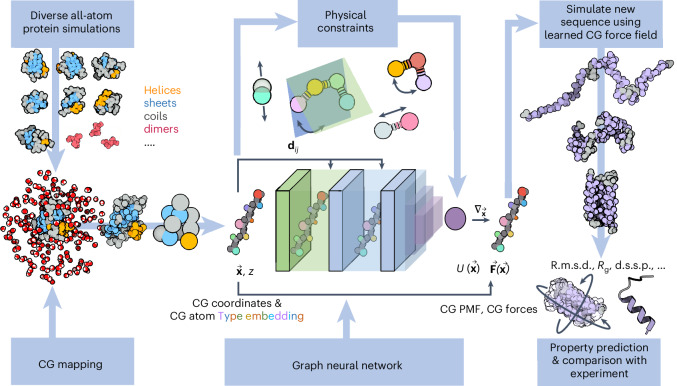
Conceptual illustration of the pipeline for building and testing a transferable, bottom–up, machine-learned, CG protein force field. Pipeline for building and testing a transferable, bottom–up, machine-learned, CG protein force field from a diverse dataset of all-atom simulations, a chosen CG resolution, and a set of basic physical prior energy terms (bonds, angles, dihedrals and purely repulsive interactions). The CG atom types *z* and CG coordinates **x** are transformed into pairwise distances **d**_*ij*_ are fed into the neural network architecture to predict the CG effective potential energy *U* and corresponding CG forces **F**. The trained neural network can subsequently be used to simulate new sequences and predict observables such as root mean square deviations (RMSD), radii of gyration (*R*_g_) or dictionaries of secondary structure in proteins (d.s.s.p.).

We generated a dataset of all-atom explicit solvent simulations of small proteins with diverse folded structures, as well as many combinations of dimers of mono- and dipeptides. Using the training data, we trained a CG force field, CGSchNet^22^, and conducted extensive simulations of the learned CG model on new, unseen proteins of various sizes and structures (details are presented in Fig. 1, Methods and Supplementary Section 1).

### Conformational landscape of peptides and small proteins

**Fig. 2:**
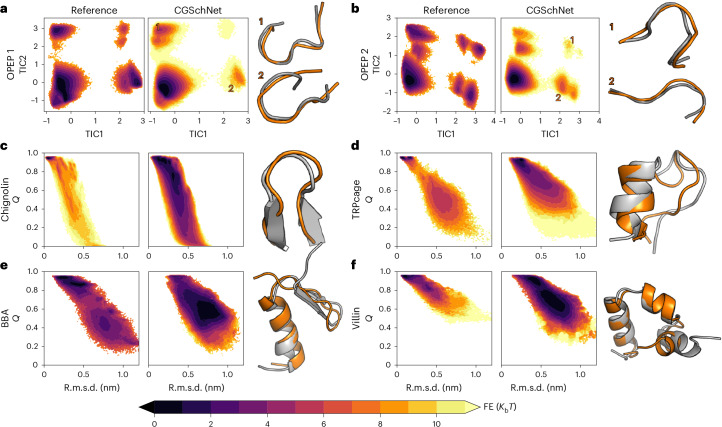
Transferable CGSchNet performance on test peptides and proteins. **a**–**f**, 8-residue peptide DYGCSIHP (**a**), 8-residue peptide SLEAGGRG (**b**), chignolin (2RVD) (**c**), TRPcage (2JOF) (**d**), BBA (1FME) (**e**) and villin (1YRF) (**f**). Each subfigure shows the two-dimensional (2D) free energy (FE) surface of the CG model (CGSchNet) and reference atomistic simulations at 300 K as a function of the first two TICA coordinates^39^ for the two 8-peptides and as a function of the fraction of native contacts, *Q*, and the C_α_ r.m.s.d. to the native state for the four small proteins. The structures shown are sampled from the most folded-like metastable basin (or labelled metastable basins for the 8-peptides) for CGSchNet (orange) and atomistic (grey) models. CG free energy landscapes are obtained through Multistate Bennett Acceptance Ratio (MBAR)-reweighted^61^ parallel-tempering simulations (details are provided in Supplementary Sections 4.2 and 6.4). Source data

To assess the ability of our approach for learning a transferable CG force field, we first tested how it reproduces the folding/unfolding free energy landscape of all-atom MD simulations for a set of unseen 8-peptides (Fig. 2a,b) and unseen small fast-folding proteins: the 025 mutant of chignolin (PDB 2RVD; Fig. 2c), TRPcage (2JOF; Fig. 2d), the beta–beta–alpha fold (BBA) (1FME; Fig. 2e) and the villin headpiece (1YRF; Fig. 2f). None of these proteins had sequence similarity >40% to any stretch of sequence from the training or validation datasets (Table 1 and Supplementary Section 5.3). The free energy surfaces of the CG model were obtained through parallel-tempering (PT) simulations to ensure converged sampling of the equilibrium distribution (Supplementary Section 4.2). Long constant-temperature (300 K) Langevin simulations were also performed for comparison, producing consistent results and multiple folding/unfolding events for all proteins (Supplementary Sections 6.4 and 6.9). We also obtained converged folding/unfolding reference landscapes from atomistic simulations for comparison.

**Table 1:** Maximum sequence similarities of test proteins to the proteins used in the model training

Test protein	Length (amino acids)	Sequence similarity to train (%)
DYGCSIHP	8	38
SLEAGGRG	8	50
Chignolin (2RVD)	10	40
TRPcage (2JOF)	20	35
BBA (1FME)	28	29
Villin (1YRF)	35	26
Homeodomain (1ENH)	54	20
SH3 (2NUZ)	55	24
CI2 (2CI2)	65	18
PaaA2 (3ZBE)	71	17
Alpha3D (2A3D)	73	19
S6 (1RIS)	97	16

Details on sequence similarity are in Supplementary Section 5.3.

The free energy landscapes of the two 8-peptides match the atomistic references closely (Fig. 2a,b). These peptides are mostly disordered, and their landscapes are mostly determined by the torsional dynamics contained in the prior energy term of the model, whereas the machine-learned multi-body terms have a small effect on the result. In contrast, for the four fast-folding proteins (Fig. 2c–f), the neural network must learn to predict the configurational landscape; control simulations with only the prior energy term only visit the unfolded state for these proteins (Supplementary Fig. 7). For these systems, the CG model predicts metastable folding and unfolding transitions, and the CG folded states are predicted with a fraction of native contacts *Q* close to 1 and low C_α_ root-mean-square deviation (r.m.s.d.) values, and they are populated with structures closely resembling the correct native state (Fig. 2c–f). For chignolin, the model is also able to stabilize the same misfolded state with misaligned TYR1 and TYR2 residues, as found in the reference atomistic simulations (Fig. 4).

For three of the four fast-folding proteins in Fig. 2, the free energy basin containing the native state is the global minimum, whereas for BBA it is a local minimum, indicating that all proteins are able to fold/unfold correctly (also Supplementary Fig. 25). However, the relative free energy differences between the metastable states do not exactly match the reference. The model performs better on chignolin, TRPcage and villin than on BBA, which contains both helical and anti-parallel β-sheet motifs. Previous CG models have also noted difficulty on this target system^24,35^.

### Extrapolation on larger proteins

**Fig. 3:**
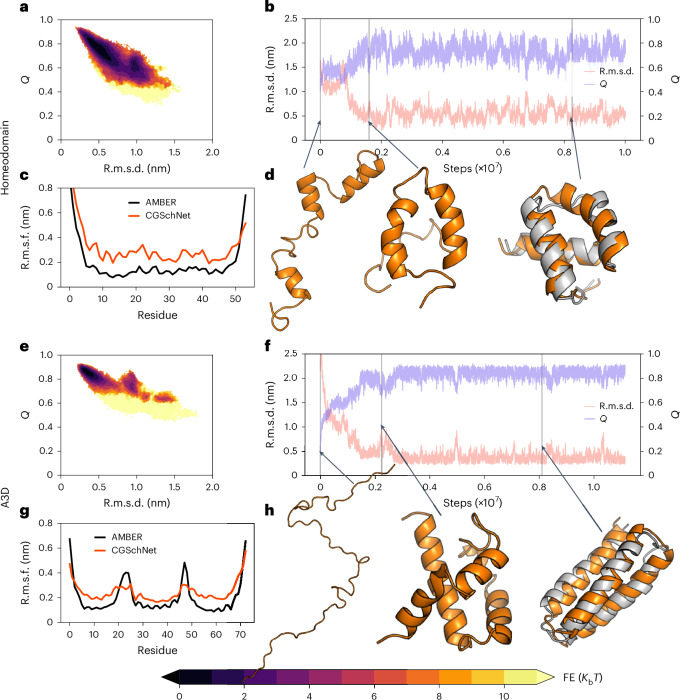
Extrapolative performance of CGSchNet on two large proteins withheld from the training and validation sets. **a**–**h**, 54-residue homeodomain (PDB 1ENH; **a**–**d**) and 73-residue alpha3D (PDB 2A3D; **e**–**h**). C_α_ r.m.s.f. values of the folded state to the crystal structure are shown (taken from a trajectory window that remained folded for more than one million MD steps; Supplementary Section 5.2) in comparison to reference (AMBER) all-atom simulations (**c**,**g**), along with an exploration of the free energy surface as a function of the fraction of native contacts, *Q*, and the C_α_ r.m.s.d. to the native state, obtained through MBAR-reweighted^61^ PT simulations (**a**,**e**). A folding trajectory starting from a completely elongated structure is shown (**b**,**f**), with orange structures illustrating folding and the crystal structure shown in grey (**d**,**h**). Source data

To assess the ability of our CG model to fold and maintain the folded states of larger and more complicated systems, we considered the following proteins: 54-residue engrailed homeodomain (1ENH) and 73-residue de novo designed protein alpha3D (2A3D) (Fig. 3). The sizes of these proteins prevent atomistic simulations from sampling the folding/unfolding transitions in reasonable time, whereas the full free energy landscape can be easily explored by the CG model. Therefore, we simulate the folded state with the atomistic force field and compared these dynamics with those of our CG model, defining the lowest free energy minimum as the CG folded state. From extended configurations, the model simulates the folding of both proteins to their correct native structure (Fig. 3a,b).

We also compared the C_α_ root-mean-square fluctuations (r.m.s.f.) within the CG folded-state free energy minimum with the reference all-atom simulations. The CG model stabilizes homeodomain in a state very close to the reference native structure, with similar terminal flexibility to the all-atom simulations (Fig. 3a, bottom left) but with slightly higher fluctuations along the length of the sequence. The difference between the folded state predicted by our model and the crystal structure (mean r.m.s.d., ~0.5 nm; fraction of native contacts, ~0.75) is similar to that of the all-atom simulations from ref. ^1^ (Supplementary Fig. 12), suggesting the difficulty in accurately predicting homeodomain’s crystalline structure.

Our reference simulations of alpha3D show flexibility at the termini as well as between each helical bundle (Fig. 3b), similar to the CG model. The CG model also stabilizes a metastable state of alpha3D very close to the native structure corresponding to an alternative three-helix bundle topology with a different packing of the helices (a detailed analysis is provided in Supplementary Section 6.8 and Supplementary Fig. 24). Alpha3D is a protein designed de novo by iteratively stabilizing the selected native state topology, and precursors of the protein populate both the native state and the alternative topology similar to the one detected by our model^36^.

These results show that the transferable machine-learned CG model can extrapolate to larger unseen proteins, stabilizing the correct native states and reproducing their associated backbone fluctuations. As an additional analysis, we also demonstrate the extrapolative performance of our CG model on stabilizing the folded states of two large proteins for which we only have experimentally determined structures as reference data, and on reproducing the conformational heterogeneity of a partially disordered protein. The results are discussed in detail in Supplementary Section 6.1.

### Detailed analysis and comparison with other CG force fields

We compared the characteristics of the learned CG energy landscapes with the reference simulations and with three other CG force fields with similar resolutions: AWSEM^17^, UNRES^16^ and Martini^15^ (Supplementary Sections 4.3, 4.4 and 4.5). We note that AWSEM is parameterized to stabilize native states^17^, and all presented targets should be stable at this temperature. Similarly, UNRES is parameterized with conformational data for multiple systems at several temperatures at ~300 K (ref. ^16^). The Martini force field is unable to stabilize the folded state of a protein without elastic restraints^37,38^; here, we show Martini simulation results without native restraints to compare the force field’s ability to explore the conformational landscape of a protein system without prior knowledge of the protein’s structure. In Supplementary Fig. 27, we show that Martini simulations with an elastic network only allow for small fluctuations around the native structure.

**Fig. 4:**
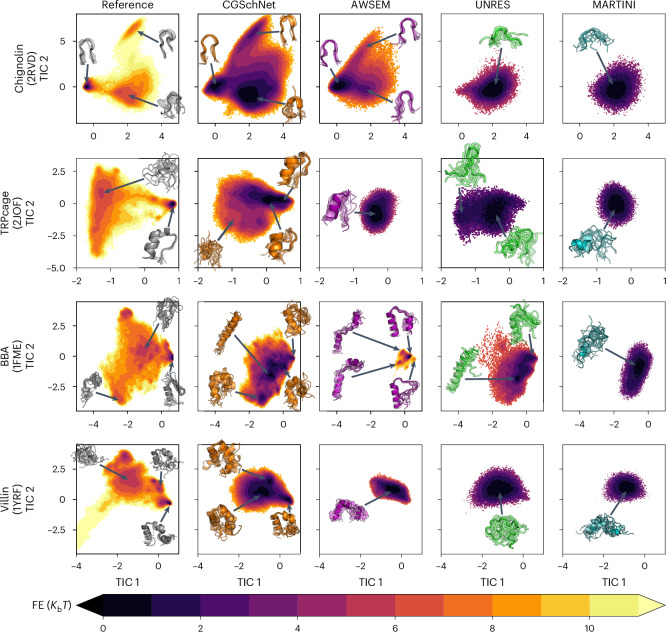
Free energy landscapes as a function of the first two TICA coordinates for four small, fast folding proteins. Results are presented for the reference atomistic simulations, for Langevin simulations of our CG model (CGSchNet), and for three different CG force fields of a comparable resolution to our CG model, AWSEM, UNRES and Martini. Each landscape includes representative structures from different metastable minima. Source data

Figure 4 shows the free energy landscapes of the four small fast-folding proteins from Fig. 2 as a function of the two slowest time-lagged independent component analysis (TICA) coordinates^39^, generated from extensive MD simulations using the reference all-atom model, CGSchNet, AWSEM, UNRES and Martini. The all-atom landscapes exhibit the most structure and have the most metastable states, whereas the CG landscapes are smoother. CGSchNet explores much of the all-atom free energy landscape and it clearly resolves folded and unfolded states as well as other metastable states, whereas this behaviour is rarely observed with the other CG models. Often, AWSEM, UNRES and Martini only stabilize a single metastable state, which is either folded or unfolded. This behaviour is expected, as both the AWSEM and UNRES force fields have been primarily parameterized for stabilizing proteins with a more pronounced fold rather than whole free energy landscapes of less stable proteins. Interestingly, there is also appreciable similarity between the all-atom reference and our machine-learned CGSchNet in structural ensembles besides the folded state prediction. For chignolin, all three all-atom main states (folded, misfolded and unfolded) are visited by both CGSchNet and AWSEM, but these are clearly metastable only with CGSchNet. AWSEM explores but does not stabilize the additional states, and UNRES and Martini do not fold chignolin at all. In the landscapes of TRPcage, BBA and villin, these differences are even more striking, as CGSchnet captures several of the metastable states of the all-atom reference, in particular those with a partial fold, but these states are not explored by the other CG force fields. Nevertheless, substantial differences in the unstructured states between the reference and CGSchNet indicate that there is still room for improvement in our CG model.

A quantitative comparison between the folded states obtained with an all-atom model for these proteins and the CG models considered here is presented in Supplementary Section 5.4. Not only does our model better populate and stabilize the native state structure than the other CG models, but it is comparable to a reference all-atom model (Supplementary Fig. 12). In particular, in the case of homeodomain (1ENH), the folded-like metastable state visited by the atomistic model is at a *Q*-value of around 0.6, lower than in our CG model.

It is important to note that our model is not designed primarily for structure prediction, but rather for the exploration of free energy landscapes for protein systems through CG MD. Unsurprisingly, structures predicted by AlphaFold3^6^ for these test proteins are very close to the corresponding crystal structures, as AlphaFold models are primarily trained to recover PDB structures.

## Beyond globular protein folding

### Folding upon binding of an intrinsically disordered peptide

**Fig. 5:**
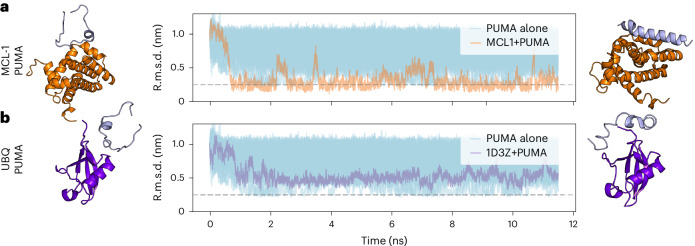
Simulation of the PUMA peptide. **a**, Time series of the r.m.s.d. of the PUMA ligand to its reference helical (folded) structure from the PDB (2ROC) when simulated alone (light blue traces) or close to the folded MCL-1 protein (orange traces). On the left and on the right are shown the starting structure and a structure at the end of the simulation, respectively. **b**, As in **a** but when the PUMA peptide is simulated close to ubiquitin (PDB 1D3Z), which is not its correct binding partner. The horizontal dashed line marks the 0.25 nm threshold for comparison. Source data

To test our CG model’s extrapolative ability beyond protein folding, we consider the PUMA-MCL-1 system as a case study of concerted folding and binding of an intrinsically disordered peptide (IDP). The disordered BH3 motif of the PUMA ligand undergoes coupled folding and binding to the induced myeloid leukaemia cell differentiation protein MCL-1^40^. Starting from an extended structure, we simulated the PUMA peptide with our transferable CG model either alone or in the presence of the folded MCL-1 protein. Figure 5 reports the evolution of the C_α_ r.m.s.d. of the ligand to its helical (folded) state during the simulations in both cases. The trajectories of the isolated PUMA (Fig. 5a,b, light blue) exhibit large r.m.s.d. fluctuations, indicating that the peptide remains disordered on its own. By contrast, the peptide simulated in the presence of the MCL-1 protein (orange) rapidly drops to an average r.m.s.d. value of ~2.5 Å, indicating induced folding of the peptide by the binding partner. The final simulation snapshot reported on the top right of Fig. 5a reinforces this result. Details about these simulations are provided in Supplementary Section 5.5.

As a control, we simulated the unfolded PUMA ligand with a protein that is not known to induce its folding, ubiquitin (PDB 1D3Z). Here, although the peptide remains close to the protein, in none of the simulated trajectories does it fold into a stable helix, as indicated by the much larger deviations of the r.m.s.d. trace (in purple), and by the final simulation snapshot on the right of Fig. 5b. Together, these results indicate that, when simulated with our CG model, the PUMA peptide forms a stable helix only when in the presence of its correct binding partner, MCL-1. Although the CG model was trained partially on interacting mono/dipeptide pairs (Supplementary Section 1.2), the training data contain no protein–protein complexes such as MCL-1/PUMA. Despite this, the model learns nontrivial interactions that can correctly model the PUMA peptide both alone and in the presence of its correct binding partner.

### Mutational analysis of ubiquitin

**Fig. 6:**
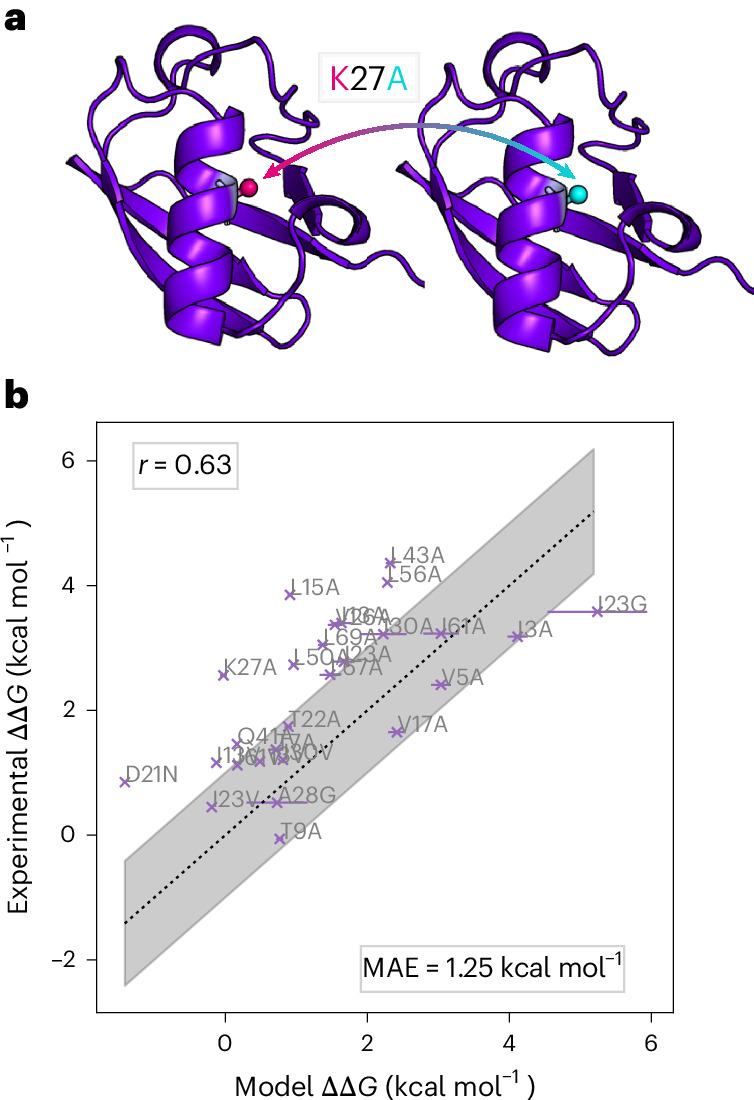
Mutational study with ubiquitin. **a**, The resolution of the CG model allows for straightforward point mutations. **b**, Comparison of the ΔΔ*G* value estimated from our model and the reference experimental ΔΔ*G* at 298 K from ref. ^41^. The Pearson correlation coefficient (*r* = 0.63) and mean absolute error (MAE = 1.25 kcal mol^−1^) are also reported. The black dotted line shows *y* = *x*, and the shaded region marks the interval *y* = *x* ± 1 kcal mol^−1^. Error bars are estimated via bootstrapping, resampling 99 times by taking batches of 10,000 independent elements of the initial folded and unfolded ensembles for each mutation. Source data

We illustrate the extrapolative power of our chemically transferable CG model in estimating folding free energy changes upon mutations, comparable to experimentally measured ΔΔ*G* values, as described in Methods and Supplementary Section 5.6. Such mutational analysis is straightforward using our CG model, because the identity of an amino acid is solely defined by the type of the C_β_ bead in our model (or the C_α_ bead for GLY): mutations can be performed simply by changing these bead types as illustrated in Fig. 6a. We chose ubiquitin as a test system, given its extensive and available experimental data, focusing specifically on the set of conservative mutations investigated by Went and Jackson^41^. We note that ubiquitin has only 18% sequence similarly with any proteins in the training/validation datasets.

Figure 6b presents the comparison between the experimental ΔΔ*G* values from ref. ^41^ and those obtained by our model as described in Supplementary Section 5.6. There is a strong correlation between our results and the experimental values; the Pearson correlation coefficient obtained with our CG model is comparable with what can be obtained with all-atom approaches^42,43^. This result indicates that the model has learned the general physical interactions among the residues at the CG resolution, thereby allowing useful predictions on new systems such as mutation effects.

## Discussion and conclusion

We have shown that it is possible to machine-learn a transferable, bottom–up, CG effective force field that can be used for MD simulation on proteins with little sequence overlap with the systems used for training the model. Notably, the number of training systems is very small, containing only 50 small protein domains and 1,245 dimers of mono- or dipeptides. We have demonstrated that the model samples similar conformational spaces as an explicit water all-atom model, but is orders of magnitude faster (Supplementary Table 9). With this increased efficiency, the CG model can characterize the folding/unfolding free energy landscapes of larger proteins where comprehensive atomistic MD is unaffordable. Despite this substantial improvement, our current MD code is not optimized, and the simulation throughput can be further improved by implementing speedups and optimizing parallel batch simulation. Our model also excels in more difficult tasks, such as predicting the folding upon binding of an intrinsically disordered peptide in the presence of its protein partner, despite the lack of protein complexes in the training dataset. However, intrinsically disordered proteins appear too structured and compact in our model, which should be a subject for future investigation. Finally, we have used the model to estimate changes in stability upon mutation in the protein ubiquitin, finding good correlation with the experimentally measured values.

In contrast to models such as AlphaFold^6^, our model is not a protein structure prediction tool. Rather, it explores the complete free energy surface of the systems of interest, including but not limited to the folded protein structure. The ultimate goal of our model would be to reproduce the thermodynamics of our systems consistently with the underlying atomistic model, but there are some protein targets, such as BBA, where our model predicts the folded state as being less stable than the unfolded. Yet, even in these systems, our model predicts a metastability in the folded region of BBA’s free energy landscape, whereas other CG models do not. Further improving the reliability of free energy predictions is an important future aim that will require both expanding the training dataset and further method development.

The key property of our CG model is the deep graph neural network (GNN) representation of its effective energy that can capture multi-body terms without imposing restrictive functional forms. The importance of multi-body terms in CG models has been extensively discussed in the literature^18,19,44–46^. Although it is expected that neural networks can capture the important multi-body effects, it is remarkable that such a CG force field is transferable in sequence space, especially given the rather small sequence coverage of the training set. A trade-off between structural accuracy and transferability has been observed in the past for various CG protein models^47,48^. However, most CG effective energy functions have been previously parameterized with pre-designed functional forms, limiting the ability to model multi-body interactions. In practice, this precluded the possibility of quantitatively investigating the accuracy/transferability trade-off in protein systems. A deep neural network is the natural answer to such a problem and allows us to address this challenge. Although this is not the first instance of a bottom–up machine-learning-based protein CG model^18,21–30^, previously proposed versions were either explicitly parameterized for single specific systems or were not transferable to proteins substantially different from those used in training/validation datasets.

The particular neural network chosen here (SchNet^49^) is quite simple. It consists of a series of continuous-filter convolutions and does not include an attention mechanism, nor explicit long-range interaction terms. This architectural choice was motivated by the goal of developing a ‘proof-of-concept’ model, that can be trained and simulated as fast as possible while still yielding the desired results. More sophisticated architectural choices could produce better-performing CG models. In particular, the lack of long-range interactions in our model may affect the model performance on much larger and multi-protein systems, where electrostatic interactions may play an important role^50,51^. Multiple approaches for including long-range interactions^52–55^ and/or attention mechanisms^56^ have been recently proposed for all-atom resolutions and could be incorporated into our modelling framework in the future.

To prevent our model from exploring nonphysical regions of conformational space, we employed a prior energy model (Supplementary Section 3.1); however, the model is quite sensitive to any change in this prior energy (Supplementary Section 3.3). The current functional form and parameterization of the prior model is the result of extensive testing, and this set of terms can be further optimized in future work.

It is also important to note that our CG model was trained at a specific thermodynamic condition. Transferability in temperature/pressure or other additional environmental parameters is therefore not expected at this point. In particular, the temperature dependence of the CG effective energy is highly nontrivial, as it really represents a free energy with an entropic component^57^. An explicit dependence of the model on thermodynamic conditions could, in principle, be included in the framework^58,59^. However, in practice, its training would require the curation of a substantially larger dataset encompassing multiple simulations at multiple thermodynamic conditions, which would probably require even more large-scale computational resources than those used in this work.

The results presented here were obtained with a model that, although aggressively coarse-grained with respect to an explicit water atomistic model, still retains the full backbone heavy atoms and an additional atom per side chain (excluding GLY). We have not yet investigated alternative resolutions for building a transferable model, and we expect transferability to be strongly tied to the chosen resolution. Although different methods have been proposed for the simultaneous optimization of CG effective energy and CG mapping^60^, it remains unclear if and which additional resolutions allow for the optimal design of a transferable and quantitatively accurate model. We believe that the results presented here open the way to a systematic investigation of this point.

## Methods

We generated a dataset of all-atom explicit solvent simulations of 50 CATH domains^62^, representing small proteins with diverse folded structures, as well as ~1,200 dimers of mono- and dipeptides. We stored all instantaneous forces on the protein atoms, performed basic force aggregation on a CG backbone representation of the proteins^27^, and trained a CG force field, CGSchNet^22^, which combines a deep GNN with physically motivated prior terms. We then conducted a series of extensive Langevin and PT simulations of the learned CG model on new, unseen proteins of various sizes and structures to demonstrate its capabilities and limitations. Wherever feasible, we also performed extensive all-atom MD simulations for the test systems and analysed them with Markov state modelling^4,5^ for comparison.

### Neural network model

Our model was built using the deep-learning Python packages PyTorch^63^ and PyTorch Geometric^64^. Building on previous efforts^22,27,33^, we chose to model the optimizable term of our CG effective energy with the GNN architecture CGSchNet, which is based on a previous architecture, SchNet^49^. See Supplementary Section 2 for a detailed description of network architecture, hyperparameter choices and training routines. The ability to directly learn species-dependent interactions and CG bead-wise features from data represents the primary advantage of using a convolutional GNN such as CGSchNet in this pursuit. More recent GNN architectures^65,66^ may be used as an alternative. However, we note that newer architectures can require more computational resources and more extensive hyperparameter searches, thereby creating substantial training barriers given the large number of MD simulation frames and the system sizes used in training.

### Loss function

We designed our CG model within the framework of variational force-matching^31,32^. In practice, we optimize the parameters {*θ*} of a network representing the effective energy ${\tilde{U}}_{\rm{CG}}({\bf{R}};\{\theta \})$, where **R** are the CG coordinates, by minimizing a loss function in the form

1 $${\chi }^{2}[{\tilde{{\bf{F}}}}_{{\rm{CG}},\,\Delta }({\bf{R}};\{\theta \})]={\left\langle \frac{1}{3N}\sum\limits_{j = 1}^{N}{\left\Vert {\left[{{\mathcal{M}}}_{F}{{\bf{f}}}_{AA}({\bf{r}})\right]}_{\Delta ,\,j}-{\tilde{{\bf{F}}}}_{{\rm{CG}},\,\Delta }{({\bf{R}};\{\theta \})}_{j}\right\Vert}^{2}\right\rangle }_{{\bf{r}}}$$

Here, *N* is the number of CG atoms in the system. In equation (1), ${\tilde{{\bf{F}}}}_{{\rm{CG}},\,\Delta }({\bf{R}};\{\theta \})$ are the forces associated with the CG effective energy, ${\tilde{{\bf{F}}}}_{\rm{CG}}({\bf{R}};\{\theta \})=-{\nabla }_{{\bf{R}}}{\tilde{U}}_{\rm{CG}}({\bf{R}};\{\theta \})$, after subtraction of the ‘prior forces’:

2 $${\tilde{{\bf{F}}}}_{{\rm{CG}},\,\Delta }({\bf{R}};\{\theta \})={\tilde{{\bf{F}}}}_{\rm{CG}}({\bf{R}};\{\theta \})-{{\bf{F}}}_{{\rm{prior}}}({\bf{R}})$$

where ${{\bf{F}}}_{{\rm{prior}}}({\bf{R}})=-{\nabla }_{{\bf{R}}}{\tilde{U}}_{{\rm{prior}}}({\bf{R}})$ and ${\tilde{U}}_{{\rm{prior}}}({\bf{R}})$ is a pre-fit ‘prior energy’ term. The atomistic force is similarly modified. The definition of a prior energy is discussed in the next section and it has been shown to play an important role in constructing stable and accurate neural network-based CG models by enforcing asymptotic physical behaviour in regions of phase space not covered adequately by training/validation datasets obtained through all-atom MD^21,22,33,34^. In equation (1), the operator ${{\mathcal{M}}}_{F}$ projects the atomistic forces **f**_*AA*_(**r**), as a function of the atomistic coordinates **r**, in CG space. We have shown in previous work that a careful choice of ${{\mathcal{M}}}_{F}$ is crucial to the optimization of the CG model^27^. In this Article, ${{\mathcal{M}}}_{F}$ is defined for each CG site as the sum of forces on the preserved atom and neighbouring hydrogen atoms connected via constrained bonds^27^.

### CG resolution and prior energy

A good choice of the prior energy model should be connected to the resolution chosen to define the CG model^34^. Previous non-transferable CG studies^22,25,27^ have utilized a resolution that retains only the C_α_ atoms for each amino acid. However, when considering 20 naturally occurring amino acids, the type enumeration for common local energy terms, such as four-body dihedral interactions, becomes very large. Efforts in the past^48^ have attempted to mitigate such scaling, but this can lead to potentially limiting or overly biasing expressions for the associated prior energies.

For this work we chose to retain the following five atoms for each residue: backbone N, C_α_, C, O and side-chain C_β_. We label different atoms with an integer atom type, with the C_β_ atom having a residue-dependent atom type number. In the case of GLY residues, which do not contain a C_β_, we retain only four atoms—N, C_α_, C and O—and assign the residue-dependent atom type number to the C_α_ atom. Supplementary Fig. 3 provides a graphical description of the CG resolution and atom type labelling.

This five-bead-per-residue CG mapping is not unprecedented—the successful AWSEM^17^ CG force field, which retains the C_α_, the C_β_ and the O atoms (as well as virtual sites for *N* and *C* atoms), utilizes a comparable resolution. This choice of CG resolution allows for a direct interpretation of secondary structures and leads to intuitive prior energy choices (for example, physical bond/angle terms, physical dihedral angles and so on). A description of the terms in the prior force field is provided in Supplementary Section 3.1.

It is important to stress that if the prior energy is used alone (without the trainable neural network energy term ${\tilde{U}}_{\rm{CG}}({\bf{R}};\{\theta \})$), it is completely incapable of stabilizing any secondary or tertiary protein structures (Supplementary Fig. 7 presents the results from control, prior-only simulations). The function of the prior energy is only to prevent the model from visiting configurationally nonphysical regions (for example, configurations involving overlapping atoms), with little to no additional bias on the configurational landscape. To illustrate the relative importance of each prior on model stability/ability to access unphysical configurations, we include a prior ablation study, where the effect of removing each prior subinteraction, one by one, is investigated on a chignolin-specific model at the same five-bead-per-residue resolution (Supplementary Fig. 10).

### Training data

Three strategies were used to create the training dataset. First, to capture sequence and secondary/tertiary structure diversity in proteins, we constructed a dataset of all-atom simulations of 50 protein domains in their native state from the CATH^62^ database (Supplementary Section 1.1 presents the domain selection procedure). Each simulation represents 100,000 frames of all-atom MD data in which the forces and positions of the solute are saved. In addition to this dataset of folded CATH simulations, we constructed a second dataset wherein ~1,200 mono/dipeptide dimer systems were simulated using umbrella sampling with dimer centre-of-mass distances as a reaction coordinate, and each system consists of 27,000 frames. Although this dataset does not contain direct secondary/tertiary structure information, it contains valuable asymptotic force information for atoms that are brought very close together through the nature of the enhanced sampling strategy. The necessity for both the CATH and dimer datasets was demonstrated by an ablation study in which we systematically remove both entire datasets and selected samples (Supplementary Fig. 20). Finally, additional frames were constructed from the previously described CATH and dimers datasets by taking every 50th frame of each simulation and additively combining bead positions with a Gaussian noise of mean 0 and standard deviation 0.5 Å (Supplementary Section 6.6 provides details of the hyperparameter selection). These distorted ‘decoy’ frames were combined with a zero delta-force label and used as additional training data, and are designed to prevent uncontrolled neural network extrapolation on distorted high-energy configurations that may arise transiently during CG simulation. Due to the induced distortion, the prior alone predicts a high baseline energy on the corresponding areas of phase space. Effectively, the decoy training data helps to ensure that the network does not attempt to predict strong forces in configurations that should be dominated by the prior terms. A comprehensive discussion on the training and validation datasets that are used to parameterize the model is provided in Supplementary Section 1.

### Mutational analysis

From the CG model, we can estimate ΔΔ*G* values by treating the effect of a single point mutation as a perturbation to the wild-type energy^67,68^. Under the assumption that the mutation does not significantly perturb the density of states, the effect of the mutation on protein stability can be estimated from perturbation theory^67,68^ (a detailed analysis is provided in Supplementary Section 5.6).

## Online content

Any methods, additional references, Nature Portfolio reporting summaries, source data, extended data, supplementary information, acknowledgements, peer review information; details of author contributions and competing interests; and statements of data and code availability are available at 10.1038/s41557-025-01874-0.

## Supplementary information

Supplementary Information Supplementary Figs. 1–28, Tables 1–9, Sections 1–6 and Discussion.

## Source data

Source Data Fig. 2 .csv files with free energy landscape data.

Source Data Fig. 3 .csv files with RMSF and free energy landscape data.

Source Data Fig. 4 .csv files with free energy landscape data.

Source Data Fig. 5 .csv files with RMSD data.

Source Data Fig. 6 .csv files with ddG data.

## Data Availability

All training data, simulation data, and trained models are available at 10.5281/zenodo.15465782 (ref. ^69^). Source data are provided with this paper.

## Code availability

Code for the generation of the training datasets is available at 10.5281/zenodo.15465782 (ref. ^69^). The scripts for model training and running simulations are available at 10.5281/zenodo.15482457 (ref. ^70^). Model training and simulation was done using the mlcg package (https://github.com/ClementiGroup/mlcg). Additional tools for the prior pipeline can be found in the mlcg-tk package (https://github.com/ClementiGroup/mlcg-tk). Finally, analysis tools can be found in the Proteka package (https://github.com/ClementiGroup/proteka).
