# Supplementary material for: Navigating protein landscapes with a machine-learned transferable coarse-grained model
Source: Nat Chem. 2025 Jul 18;17(8):1284–92. doi: 10.1038/s41557-025-01874-0 (PMC12313527; doi:10.1038/s41557-025-01874-0)
Supplement: Supplementary file 1 — Supplementary Figs. 1–28, Tables 1–9, Sections 1–6 and Discussion. [file 41557_2025_1874_MOESM1_ESM.pdf]

nature chemistry

Supplementary information

https://doi.org/10.1038/s41557-025-01874-0

Navigating protein landscapes with a machine-learned transferable coarse-grained model

In the format provided by the authors and unedited

Supplementary Information

Contents

1 Data Preparation **3**
1.1 CATH Domains 3
1.2 Dimers 4
1.3 Decoys 4
1.4 Octapeptides 4
1.5 Training and validation set 4
1.6 Fast folders 5
1.6.1 Chignolin 5
1.6.2 TRPcage & BBA 5
1.6.3 Villin 5
1.6.4 Alpha 3D & Homeodomain 5
1.6.5 Markov State Models 6

2 Network model **6**
2.1 Atom type labeling 6
2.2 Network architecture 6
2.3 Training 7

3 Prior model **9**
3.1 Definition of the prior terms 9
3.2 Prior-only simulations 11
3.3 Prior studies on protein-specific models 11

4 CG simulations **14**
4.1 Langevin simulations 14
4.2 Parallel-tempering simulations 15
4.3 UNRES simulations 15
4.4 AWSEM simulations 16
4.5 MARTINI simulations 16

5 Analysis details **16**
5.1 Calculation of fraction of native contacts, Q 16
5.2 Calculation of the root mean square fluctuations 17
5.3 Extrapolation target sequence similarity 17
5.4 Computation of the structural metrics 18
5.4.1 Structural metrics 18
5.4.2 GDT-TS score 19
5.4.3 Extraction of structure ensembles 19
5.4.4 Computation of the highest metastable Q 22
5.5 Details on the MCL-1/PUMA simulations 22
5.6 Details on the Ubiquitin mutational study 23

6 Additional analysis **24**
6.1 Additional systems 25
6.2 Computational efficiency of CG simulations 26
6.3 Comparison to previous machine-learned CG models 28
6.4 Comparison between Langevin and parallel-tempering simulations 28
6.5 Dataset ablation study 30

6.6 Decoy study . . . 31
6.7 Timestep analysis . . . 32
6.8 Detailed analysis of alpha3D free energy landscape . . . 33
6.9 Langevin trajectories and folding/unfolding events . . . 34
6.10 Simulation of training proteins . . . 34
6.11 Martini large protein simulations . . . 36
6.12 Contact maps . . . 37

1 Data Preparation

1.1 CATH Domains

A set of candidate 14942 non-redundant structural domains was selected from the CATH database [1]. From this set, domains were further excluded if: 1) they contained non-contiguous structures/sequences, 2) they had less than 50 residues, 3) they had more than 75 residues, 4) they had a relative shape anisotropy of > 0.04, or 5) they had a combined helix and sheet fraction (as defined by coarse DSSP [2] using MDTraj [3]) of less than 50 percent. Applying these exclusions resulted in a compact set of 95 non-redundant domains. From this set, a final set of 50 domains, with a nearly equal fraction of helix to beta-sheet secondary structure, was formed. A tiled ribbon representation of all 50 chosen CATH domains is shown in Supplementary Fig. 1 along with their respective sequence length.

For each sequestered CATH domain in the final set, structures and topologies were processed from molecular dynamics (MD) simulations using OpenMM and tools from GROMACS [4]. Each domain was solvated and equilibrated using Gromacs, using the AMBER ff-99SB-ILDN force field [5] and the TIP3P water model [6]. All domains were neutralized and simulated at a concentration of 0.1M NaCl. All heavy-hydrogen bonds were constrained and the hydrogen masses were increased by a factor of 4. PME was utilized with a 1.0 nm grid spacing, while for nonbonded interactions a cutoff at 0.9nm and a switching distance of 0.75 nm were employed. The friction constant was set to 1 ps^{-1}, and a timestep of 4 fs was used. After this, four 0.5-μs production all-atom simulations at 300 K were run for each domain in OpenMM using Langevin dynamics, resulting in 2 μs of simulation for each domain and 100 μs of simulation coordinates and forces across all final CATH domains. Coordinates and forces of protein atoms were stored at a 20 ps interval.

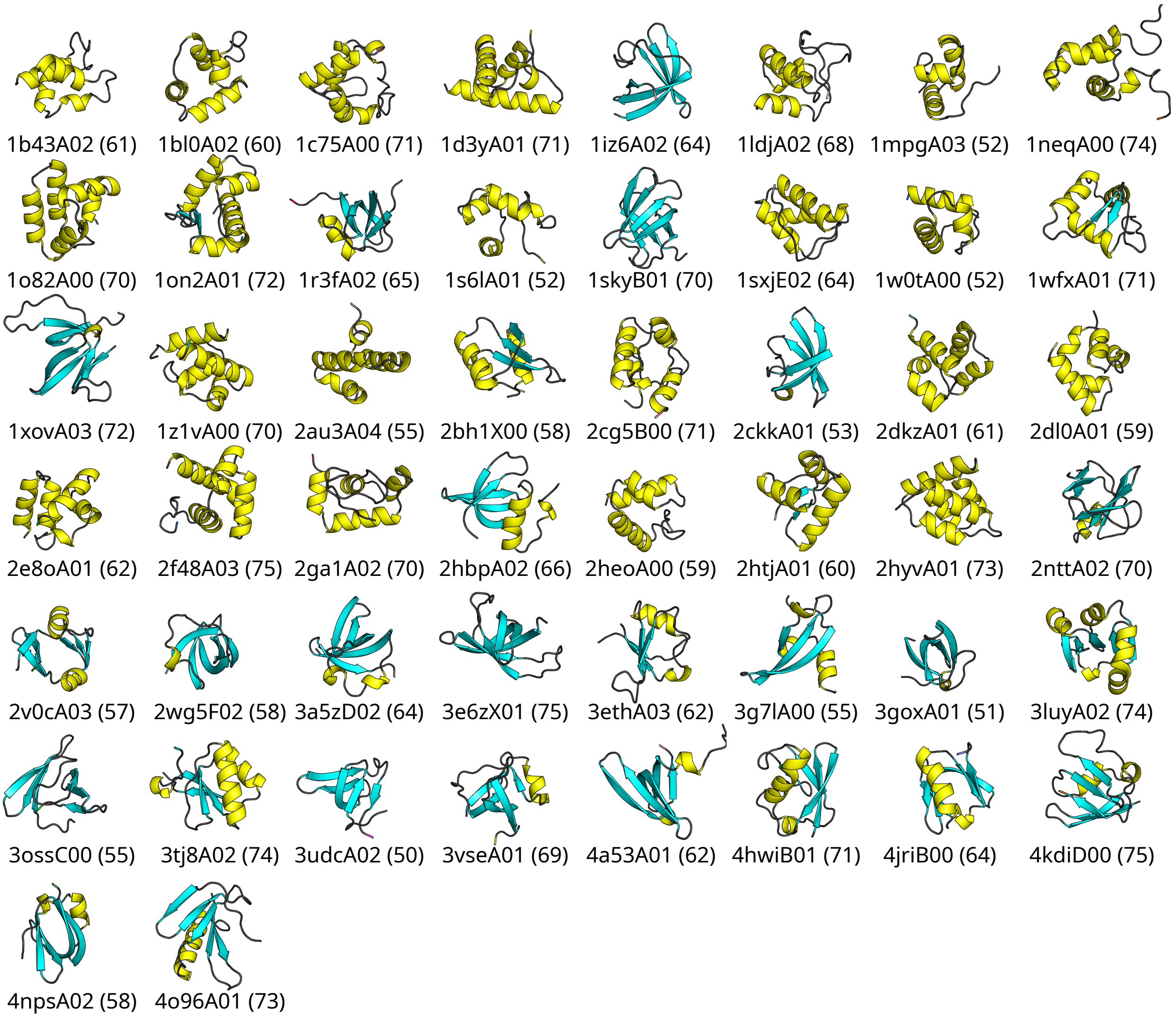

Fig. 1: Chosen set of 50 CATH Domains: each label contains the name of the CATH domain an its sequence length. Disordered regions are colored black, helical regions are yellow, and sheet regions are blue. Figure generated using PyMOL [7].

1.2 Dimers

1245 systems of capped mono/dipeptide dimer pairs for a variety of sequences were solvated and prepared using OpenMM [8]. Umbrella sampling simulations were carried out with the center of mass (COM) distance between the molecules as a reaction coordinate, which varied on a linear grid between 3 and 30 Å using a force constant of 500 kJ/mol/nm^2. The same force field, temperature, and simulation settings were used as described in Supplementary Section 1.1, though the integration timestep was set to 2 fs and no hydrogen mass re-partitioning was in effect. At each window, systems were equilibrated using NPT for 50 ps with a high friction constant 100 ps^{-1}, followed by production simulation for 1 ns. Protein coordinates and forces were saved from production runs every 1 ps. Configurations were used without reweighting for force-matching.

1.3 Decoys

In combination with the delta-learning strategy introduced in the main text, we propose an empirical approach to asymptotically force the learned model to the behavior of the prior energy term near unphysical configurations. To this aim, we introduce so-called "decoy" configurations into the training dataset. These decoys correspond to configurations where the CG beads positions are modified through the addition of Gaussian noise and associated with a zero force label. This approach is inspired by our recent work [9] and is designed to enrich the delta-learning dataset with artificial, unphysically distorted configurations, with the zero force label encouraging the network to rely solely on the prior potential in these areas of phase space. Various amounts of decoys and noise levels were tested (see Supplementary Section 6.6 and Supplementary Fig. 21). The final chosen model contains one decoy frame for every 50 original frames, with a Gaussian noise of 0.5 Å added to each CG bead.

1.4 Octapeptides

In order to parametrize the transferable prior model for the delta learning scheme, a diverse set of octapeptide sequences were simulated using MD to create a fitting dataset for the prior interaction terms described in Supplementary Section 3. A set of candidate sequences was picked from the PISCES database [10] using default query parameters and was cut into 3,326,176 octapeptide subsequences. Each subsequence was featurized into a 6-dimensional vector containing the following physicochemical properties from Table 3 in [11]: RF (chromatographic relative-to-front) rank, bulkiness, pK1, isoelectric point (pI), hydrophobicity, and polarity. Principal component analysis (PCA) was then used to obtain a reduced-dimension representation of these features across all peptides by projecting onto the first two principal components. K-means clustering was then performed in this reduced space with 2000 cluster centers. Random subsequences were sampled from the cluster centers until 1100 unique subsequences were obtained.

For each of the 1100 sequences, an octapeptide structure was generated using PyMol [7]. These structures were then solvated and equilibrated using Langevin dynamics in ACEMD [12], using the same parameters as described in Supplementary Section 1.1 with the difference that the friction was set to 0.1 ps^{-1}. Production simulations at 300K were run for each peptide, in which the adaptive sampling strategy described in [13] was used to generate around 100 all-atom trajectories, each about 10 ns long for a total of 1 μs simulation time for each peptide. The entire completed dataset consisted of 1.1 ms of all-atom simulation coordinates and forces.

Two peptides, DYGCSIHP and SLEAGGRG, were selected for further, longer simulation to prepare reference free energy surfaces (FESs) to test the extrapolation of the transferable CGSchNet. For each of these two peptides, an initial configuration was chosen randomly from the original octapeptide dataset described above, and a single, long, all-atom MD simulation was performed with the same settings in OpenMM for a total of 3.5 microseconds, saving every 2 ps.

To ensure a complete convergence of the MD simulation, a Markov state model (MSM) [14, 15] was evaluated for the two molecules. Considering sin and cos of the ϕ and ψ dihedral angles, together with the distances between all C_α atoms as features, a TICA [16] model was built, with a lag-time of 1000 frames (2 ns). This model was also used to project the CG simulation results. A k-means clustering with 500 cluster centers was then performed for the estimation of a maximum likelihood MSM using a lagtime of 20 frames (0.04 ns). The MSM was also used to reweight the trajectory. Supplementary Fig. 2 shows the convergence of the MSM implied timescales for both systems.

1.5 Training and validation set

Training and validation sets were constructed exclusively from the dimer and folded CATH simulations and the generated decoys; the octapetides were only used as part of the prior model parametrization (see Supplementary Section 3). To construct the training and validation sets for the network, dataset splits were made on a *molecular* basis. That is, for each subdataset (dimers or CATH), the molecule identities of that subdataset were further partitioned according to a random 80/20 % train/validation split. Every 50th frame for each molecule in the training set was further noised and added as an additional decoy frame to the training set. In the end, the training/validation set each consists of a selection of dimer molecules, CATH molecules and decoy molecules. Furthermore, 6 purely helical CATH domains were discarded from the

final CATH training set to better balance the ratio of helix to sheet structures. In this way, the validation loss assessed how well the model was learning to predict CG forces on sets of molecules (sequences and structures) that were never seen by the network during training. Importantly, none of the extrapolation targets or tasks presented in the main text were part of these training or validation sets.

1.6 Fast folders

To generate the reference data used to test the CG model, we simulated several proteins found in, or similar to, the fast folder suite studied by Lindorff-Larsen et al [17]. These proteins are listed in Supplementary Table 1 below. Simulation parameters including the force field and the integration settings are identical to the ones described in the dimer dataset above (see Supplementary Section 1.2). The sampling strategy used for each protein differed. In the case of TRPcage, BBA, and Villin, trajectories starting from different portions of phase space were used. For the remaining proteins, multiple trajectories were started from a single configuration. A summary of the aggregate simulation length and number of constituent trajectories for these proteins is given in Supplementary Table 1. The trajectories for Chignolin, TRPcage, BBA, and Villin were postprocessed using MSMs.

Name (PDB)	# Traj.	Aggr. length (μs)	Median length (μs)
Chignolin (2RVD)	20	100	5.0
TRPcage (2JOF)	3226	1613	0.5
BBA (1FME)	3514	1757	0.5
Villin (1YRF)	147	747	4.9
Alpha 3D (2A3D)	24	89	4.2
Homeodomain (1ENH)	28	78	3.8

Table 1: Description of atomistic MD trajectories used for analyzing select fast-folding proteins.

1.6.1 Chignolin

20 independent simulations were run for 5 μs with the coordinates of protein atoms saved at a 50 ps interval. All simulations were started from the same extended structure. The non-bonded cutoff was set to 1.0 nm and no switching function was used.

1.6.2 TRPcage & BBA

3525 (TRPcage) and 3295 (BBA) starting configurations were taken from a previously published study [18]. These simulation starting points were selected at random from a 2D slow collective variable projection computed with TICA with a 120 ns lagtime using the pairwise distance between the C_α atoms. In the case of TRPcage, the final four C_α atoms were discarded as this region is highly disordered and does not correspond to large scale protein motion. In the slow collective variable space, bins were placed uniformly and 5 structures from each bin were selected and run for 500 ns with positions saved every 2 ps.

1.6.3 Villin

In total, 151 individual trajectories were initialized in different parts of Villin's phase space and simulated for approximately 5 μs each. Of these, an initial set of simulations were started from folded and unfolded structures; after completion, simulations that explored novel parts of phase space were extended with an additional round of simulations manually spawned from their endpoints. This process was repeated two more times to generate a collection of 151 trajectories. Generated trajectories varied in exact length due to fixed walltime limits and varying hardware. 4 trajectories were removed from the analysis as they irreversibly entered misfolded states and could not be included in the MSM analysis, reflecting the fundamental difficulty in fully converging atomistic protein simulations on consumer hardware. Frames were saved every 10 ps.

1.6.4 Alpha 3D & Homeodomain

24 (Alpha 3D) and 28 (Homeodomain) MD simulations were started from the folded structure found in the PDB. Individual simulations were approximately 5 μs in length. These two proteins exhibit neither unfolding nor any large-scale fluctuations during the atomistic simulations. Frames were saved every 10 ps.

Protein	TICA input features	# clusters	Lagtime (ns)
Chignolin	Pairwise CA distances	500	5
TRPcage	Pairwise CA distances of all but the first residue	500	5
BBA	Sine and cosine of CA dihedral angles	350	5
Villin	Pairwise CA distances	250	250

Table 2: MSMs hyperparameters. The MSMs were estimated using the Deeptime library [19].

1.6.5 Markov State Models

To obtain a converged FES from short simulation trajectories for Chignolin, TRPcage, BBA, and Villin, an MSM was estimated for each ensemble of trajectories. First, TICA was performed to obtain the dominant TICs, and the MSM analysis was performed in this projected, 2D space. We discretized the trajectories using k-means clustering over the first 2 TIC coordinates and, from this discretization, a maximum-likelihood MSM was estimated. Hyperparameters for this analysis are reported in Supplementary Table 2. The lagtimes were selected after visually inspecting a plot of the implied timescales as a function of the lagtime. For Villin, as the first two processes do not converge, the lagtime was selected based on the convergence of the third process. Timescale plots are shown in Supplementary Fig. 2. We note that in the case of Villin, no trajectories spontaneously unfolded (after removing those 4 that irreversibly misfolded, as mentioned in Supplementary Section 1.6.3) and all 8 trajectories started in the unfolded state quickly refolded, supporting the conclusion that the unfolded state of Villin is not stable at these thermodynamic conditions. This phenomenon was reaffirmed by the MSM reweighting process.

2 Network model

2.1 Atom type labeling

CG atoms were integer-typed using the following strategy: integers 1-20 were used to type the C_β beads according to their amino-acid identity (with the exception of GLY residues, in which the associated C_α bead was typed with the integer representing GLY), while integers 21-24 were used to type the non-C_β beads according to their backbone atom identity (see Supplementary Fig. 3 for a visual description).

2.2 Network architecture

For a molecular graph of N nodes, corresponding to a CG molecule of N CG atoms, a vector $\mathbf{z} \in \{1, 2, \dots, K\}^N$ of CG atom types (in our case, $K = 24$) are input into an initial (learnable) embedding layer that maps to embedded features $\mathbf{x}^0 \in \mathbb{R}^{N\times H}$:

$$\mathbf{x}^0 = \text{Embedding}(\mathbf{z}) \tag{1}$$

wherein $\mathbf{x}_i^0 \in \mathbb{R}^H$ constitutes a vector of initial node properties for the node corresponding to the CG bead i. The CG atom properties are then refined through successive network *interaction blocks*, which model CG bead interactions through edge features and *continuous filters*. For a CG bead i, a corresponding set of edge attributes $\{E_{i1}, ..., E_{ij}, ..., E_{iJ}\}$ is generated from its J CG bead neighbors contained within a finite radial cutoff, r_{cut} centered on CG bead i. The initial edge attributes are chosen to be pairwise distances linearly expanded into a basis of M univariate radial basis functions $\{f_1, ..., f_m, ...f_M\}$:

$$E_{ij} = [\alpha_m f_m (|\mathbf{r}_i - \mathbf{r}_j|)]_{m=1,\cdots,M} \tag{2}$$

where $\mathbf{r}_i \in \mathbb{R}^3$ is the Cartesian position vector of CG bead i and $\{\alpha_1, ..., \alpha_M\}$ are (learnable) basis expansion coefficients. This edge information is used as input to a *filter generating network*, $\mathcal{W}(\cdot)$, whose outputs represent continuous filter values for a set of L filters. The filter-generating network is chosen to be a multi-layer perceptron (MLP), consisting of two linear transformations separated by an element-wise non-linear activation function σ. It is useful (especially for large molecules) to apply an additional multiplicative *filter cutoff*, $\mathcal{F}(|\mathbf{r}_i - \mathbf{r}_j|)$ to the generated filters [20]. As such, the full *interaction block* refinement of the embedded CG features is defined as:

$$\mathbf{x}_i^t = \mathbf{x}_i^{t-1} + W^{t,3}\sigma\left(W^{t,2}\left(\sum_{j=1}^{J} W^{t,1}x_i^{t-1} \circ [\mathcal{F}(|\mathbf{r}_i - \mathbf{r}_j|) \circ \mathcal{W}(E_{ij})]\right)\right) \tag{3}$$

where $W^{t,1}$, $W^{t,2}$ and $W^{t,3}$ are trainable weight matrices for the linear transformations before and after convolution and activation, and embedded feature refinements are added residually to the initial node feature $\mathbf{x}_i^{t-1}$. Case $t = 1$ shows that

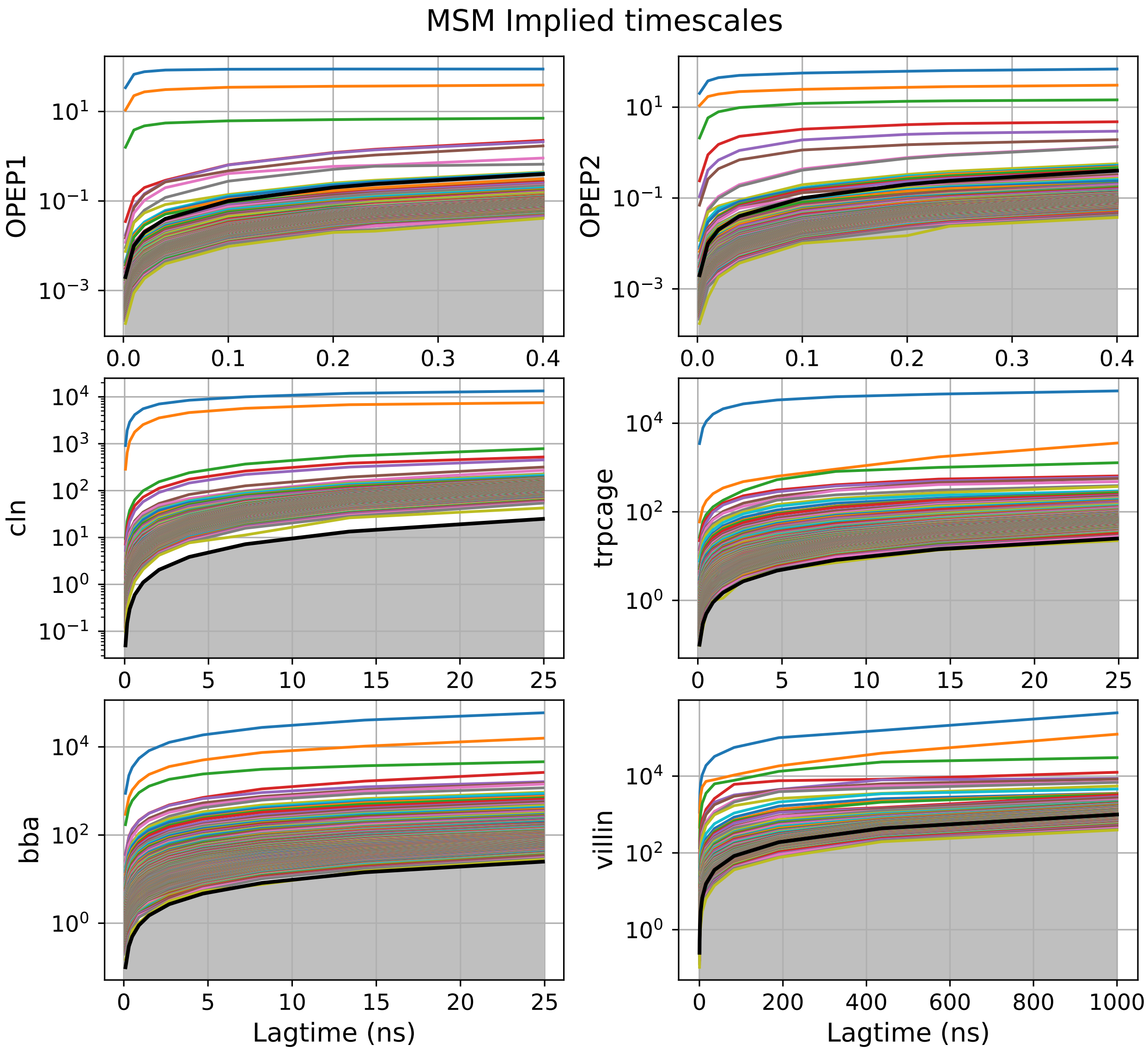

Fig. 2: MSM implied timescales as functions of lagtime for the 2 small peptides OPEP 1 (DYGCSIHP) and OPEP 2 (SLEAGGRG) and 4 fast-folding proteins. In all plots, the Y-axis is in ns with a log-scale.

the first interaction block incorporates relative positional information of the nodes into the initial embedded features $\mathbf{x}^0$. In general, multiple interaction blocks may be assembled in series when $t \in \{1, 2, \cdots, T\}$ with T representing the number of interaction blocks in the network. Analogous to deep convolutional neural networks, the continuous filter convolutions over several interaction blocks gradually incorporate information from bead pairs that are further and further apart, including those beyond the fixed cutoff radius defined for a single interaction block.

Lastly, the output of the final interaction block is fed through a small MLP that contracts down to a scalar prediction of the CG potential of mean force (PMF):

$$U = \sum_{i=1}^{N} \mathrm{MLP}(\mathbf{x}_i^T) \tag{4}$$

from which CG forces can be extracted through a gradient operation w.r.t. to the input CG configuration coordinates and be used in a force matching loss [21]. Supplementary Table 3 provides the SchNet neural network hyperparameters used in this work.

2.3 Training

Models were trained using data from the shuffled training set with the Adam optimizer [23], a base learning rate of 0.0001 and a batch size of 80 molecules. In every training/validation batch, the ratio of CG beads from CATH systems to those from dimer systems was fixed to 10:1 on average (with deviations due to random size differences between sampled

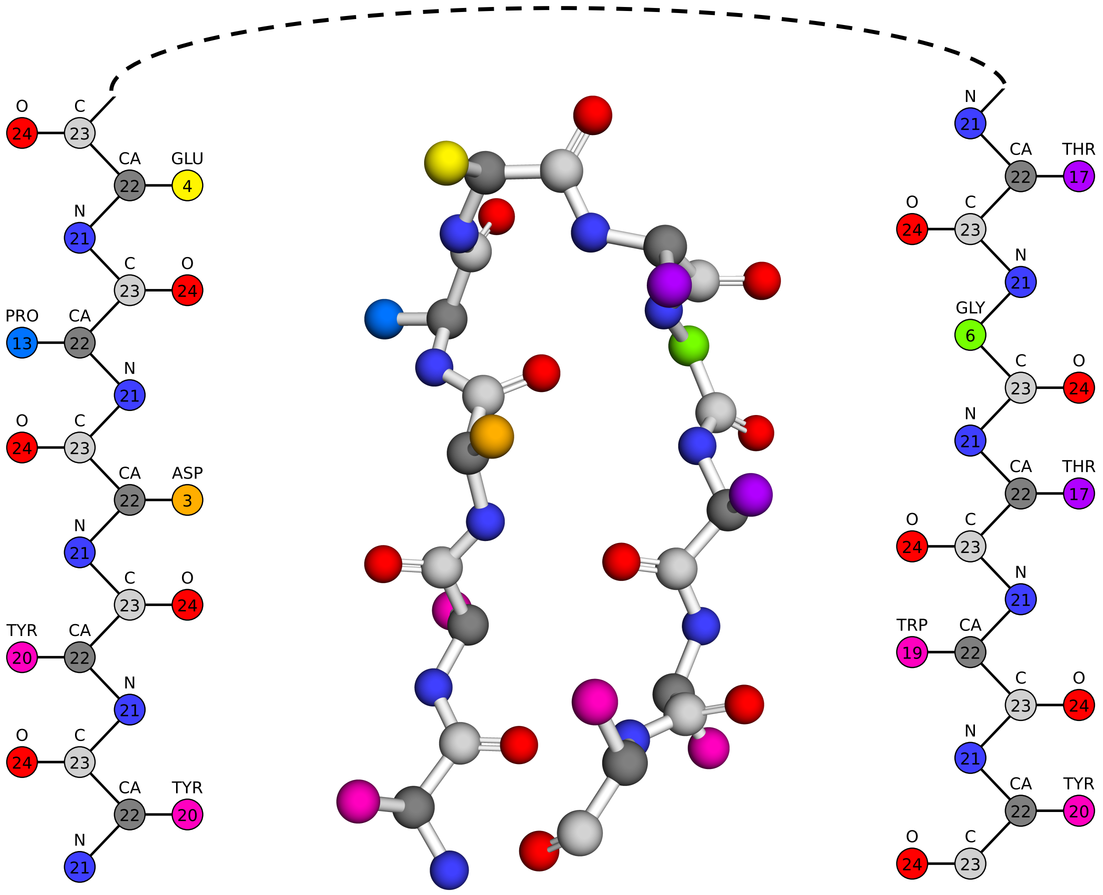

Fig. 3: Graphical representation of the embedding and CG resolution for Chignolin. Left and right: a 2D representation of the protein at the CG resolution, with the type number inside the bead. Center: 3D representation of the protein in its folded state at the CG resolution.

Embedding Feature Size (H)	128
Number of Basis Functions (M)	64
Number of Filters (L)	128
Interaction Blocks (T)	4
Lower Cutoff	0
Upper Cutoff	15 $\AA$
Distance Expansion Basis	PhysNet [22]
Output Network	MLP, 2 layers, [128,64] features
Output Prediction	Scalar energy, Delta forces

Table 3: Neural network hyperparameters

systems during batch construction). All training was done using PyTorch Lightning [24]. All training was performed using computational resources at both Freie Universität Berlin and the Zuse Institute Berlin. Batch order was fixed to be the same every time the training was restarted/extended. Supplementary Fig. 4 shows the loss over the validation set of molecules as a function of the training epoch. Training took about 50min/epoch on 16 NVIDIA A100 GPUs. The final model was selected at epoch 73, choosing the best performing epoch among a selected set of simulated epochs, as shown in Supplementary Fig. 5. This epoch is not associated to the lowest validation loss, as the force-matching validation loss alone is not a sufficient metric to identify the highest-performing model for simulation, and multiple models corresponding to different epochs need to be tested to arrive at the most stable force field. Our model selection strategies align with previous similar works [9, 25] and in part stem from the intrinsic noise of the loss function as a result of the CG mapping operator [18, 21, 26, 27].

In order to assess the reproducibility of the method, we train three additional replicas using our original model setup with a different random seed. All other hyperparameters and training data are kept the same as in the original model. The random seed affects various aspects of training including the initialization of the training weights and batch selection. All models were trained up to an epoch similar to the one chosen for the original model. As one can see in Supplementary Fig. 6 all four models perform comparatively well on the four fast-folders.

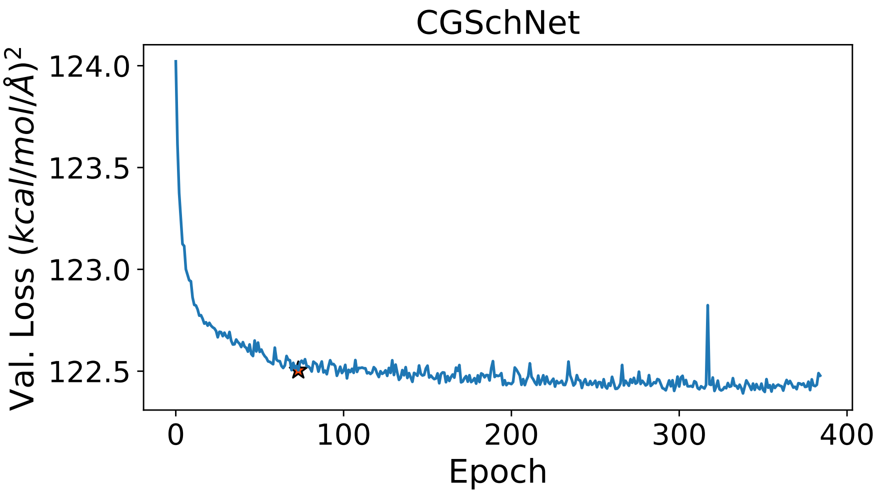

Fig. 4: Validation loss for the transferable model as a function of the training epoch number. The orange star shows the epoch for which the main transferable model was chosen. Note that the validation set contains systems of many different sizes, and as such the absolute loss is not as meaningful. The unoptimizable portion of the force matching loss function is a function of an (unoptimizable) CG mapping operator, a point demonstrated in previous studies [18, 21, 26, 27].

3 Prior model

We have shown in previous works [21, 26] that training a neural network on only atomistic simulation data can generate CG force fields that explore unphysical states and lead to diverging and unrealistic simulation results [21]. These poor force predictions stem from the absence of unphysical configurations in the training data, as atomistic simulations do not explore such regions of phase space. To mitigate this limitation, we add a prior energy term to our force field which ensures that the free energy of the final model approaches infinity for configurations which are unrealistic for a given system, including those with stretched bonds, distorted dihedrals, or overlapping atoms. Our prior model does not include any attractive non-bonded terms, therefore these remain to be learned by the neural network.

3.1 Definition of the prior terms

Supplementary Table 4 enumerates the features, interaction types, and specificity of priors that together constitute the prior model. All terms in the prior model are fit and fixed *before* the neural network begins training. Parametrization of each prior term (aside from the repulsions) was achieved through SciPy [28] curve fitting. Harmonic interactions have the following form:

$$V(x) = V_0 + k(x - x_0)^2 \tag{5}$$

where x can either be a pairwise distance or a triplet angle, V_0 is constant offset, k is a stiffness constant, and x_0 is an equilibrium distance/angle. All dihedral priors (including the two improper dihedrals, $\Gamma_{1/2}$) are parametrized to the following truncated Fourier series:

$$V(\theta) = V_0 + \sum_{n=1}^{n_{\text{deg}}} k_{1,n} \sin(n\theta) + k_{2,n} \cos(n\theta) \tag{6}$$

where V_0 is again a constant offset, n_{deg} determines the maximum frequency term, and $k_{1/2}$ are strength coefficients for each *sin* and *cos* term respectively. For phi dihedrals, all amino acids were constrained to $n_{deg} = 3$, except for PRO, which was constrained to $n_{deg} = 1$. For psi dihedrals, all amino acids were constrained to $n_{deg} = 3$. For omega dihedrals, all amino acids were constrained to $n_{deg} = 1$, except for PRO, which was fit with $n_{deg} = 2$ to allow for cis/trans isomerization. For all Γ dihedral priors, $n_{deg} = 1$. For the prior repulsion, all atom pairs separated by 6 or more bonds contributed the following energy:

$$V(r_{ij}) = \left(\frac{\sigma_{ij}}{r_{ij}}\right)^6 \tag{7}$$

where r_{ij} is the pairwise distance between atoms i and j, and σ_{ij} is their mutual excluded volume in 1-D. σ_{ij} was estimated for all pair types by selecting the minimum observed pairwise simulation distance for those pair types. For C_α and O pairs, we found it important to further increase the value to the 0.1 percentile of the observed distances in order to prevent the CG model from becoming too sticky due to otherwise poor network extrapolation.

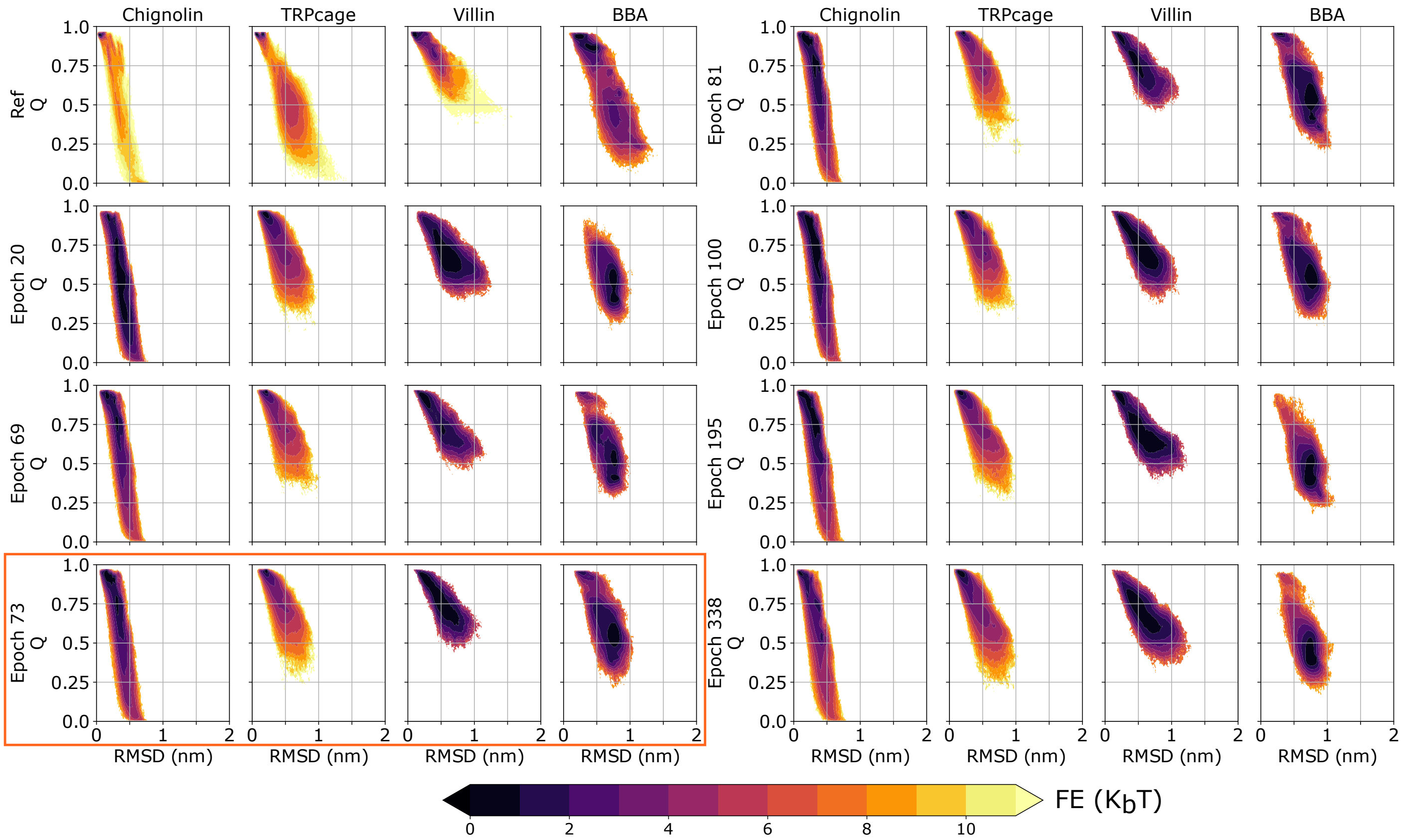

Fig. 5: Performance of the trained model on the four fast-folders Chignolin (2RVD), TRPcage (2JOF), BBA (1FME) and Villin (1YRF) at different training epochs. The selected model is outlined in orange.

Type	CG atoms	Energy Function	Specificity
Physical Bond	$N_i^n - C_{\alpha,j}^n$	Harmonic	N/Non/C-term
Physical Bond	$C_{\alpha,i}^n - C_j^n$	Harmonic	N/Non/C-term
Physical Bond	$C_i^n - O_j^n$	Harmonic	N/Non/C-term
Physical Bond	$C_i^n - N_j^{n+1}$	Harmonic	N/Non/C-term
Physical Bond	$C_{\alpha,i}^n - C_{\beta,j}^n$	Harmonic	N/Non/C-term/Residue
Physical Angle	$N_i^n - C_{\alpha,j}^n - C_k^n$	Harmonic	N/Non/C-term
Physical Angle	$C_{\alpha,i}^n - C_j^n - O_k^n$	Harmonic	N/Non/C-term
Physical Angle	$O_i^n - C_j^n - N_k^{n+1}$	Harmonic	N/Non/C-term
Physical Angle	$N_i^n - C_{\alpha,j}^n - C_{\beta,k}^n$	Harmonic	N/Non/C-term/Residue
Physical Angle	$C_{\beta,i}^n - C_{\alpha,j}^n - C_k^n$	Harmonic	N/Non/C-term
Phi Dihedral	$C_i^{n-1} - N_j^n - C_{\alpha,k}^n - C_l^n$	Fourier Series	Residue
Psi Dihedral	$N_i^n - C_{\alpha,j}^n - C_k^n - N_l^{n+1}$	Fourier Series	Residue
Omega Dihedral	$C_{\alpha,i}^{n-1} - C_j^{n-1} - N_k^n - C_{\alpha,l}^n$	Fourier Series	Residue
Γ_1 Dihedral	$N_i^n - C_{\beta,j}^n - C_k^n - C_{\alpha,l}^n$	Fourier Series	Residue
Γ_2 Dihedral	$C_{\alpha,i}^n - O_j^n - N_k^{n+1} - C_l^n$	Fourier Series	Residue
Repulsion	≥ 6 beads apart	Inverse Power Law	CG bead type

Table 4: Definition of prior energy terms. n indexes the residue, while $i - l$ indexes the atom sequence defining the feature. For each listed subset of interactions the parameters were fitted independently.

The prior model was parametrized directly through Boltzmann inversion using the above-described octapeptide and CATH datasets (using every 100th frame to reduce memory demands). We found it necessary to fit different sets of parameters for subsets of the same type of interactions based on the general position (e.g., in the middle or on the N- or C-terminal of a peptide) and/or the chemical identity of the involved residue. Because the prior model contains no attractive interactions, it cannot stabilize compact folded states or secondary structures, such as helices or beta sheets, as can be seen in Supplementary Fig. 7.

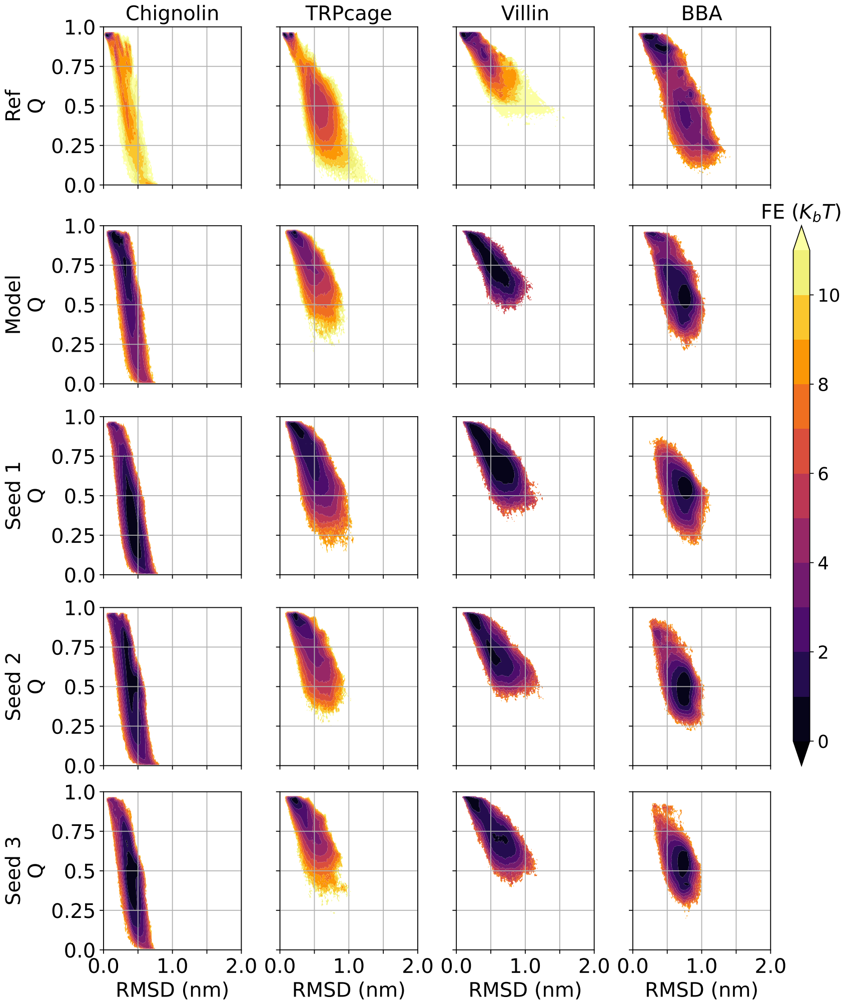

Fig. 6: Performance of models trained with different training seeds. The first row contains the all-atom AMBER reference simulations and the model presented in the main text is on the second row.

3.2 Prior-only simulations

We conducted direct MD CG simulations of all proteins for which free energy surfaces are shown in the main text, by using only the prior energy model, thus with no learned network interactions. This illustrates the corrections to the underlying prior model that the CGSchNet model is learning. These simulations are shown in Supplementary Fig. 7.

As expected, in the prior-only simulations, all proteins unfold and remain unfolded throughout the simulation. Interestingly, while results similar to the the trained CG model and the reference atomistic simulations are obtained for the two 8-residue peptides, the 71-residue intrinsically disordered peptide explores a rather different configurational landscape with respect to CGSchNet when simulated with only the prior energy model. The prior-only model primarily explores a region of the free energy landscape poorly populated by the CGSchNet model, suggesting that the prior only model has no information about stabilizing interactions which are learned in the trained model. This region corresponds to an entirely disordered ensemble, while our trained model is able to reproduce the secondary structure distribution of the NMR ensemble. It is thus clear that the simulation results obtained by our trained CG model arise from the ability of the network to learn the necessary multi-bodied interactions and corrections to the underlying prior model.

3.3 Prior studies on protein-specific models

In order to rule out potentially poor prior modeling choices at the 5-CG-bead-per-residue resolution, a protein-specific (non-transferable) model of Chignolin (2RVD) was trained using the same hyperparameters and training routine as the transferable model and subsequently simulated to see if it could reproduce the free energy landscape of the reference dataset. For the reference dataset, we chose to use an extended version of the AMBER Chignolin dataset described previously (see Supplementary Section 1.6.1). We started 1000 independent simulations from configurations obtained from a higher temperature simulations and ran them for 50ns each. The non-bonded cutoff was set to 0.9nm and all other parameters and force-field were the same as previously described in Supplementary Section 1.6.1. In total, 5 million

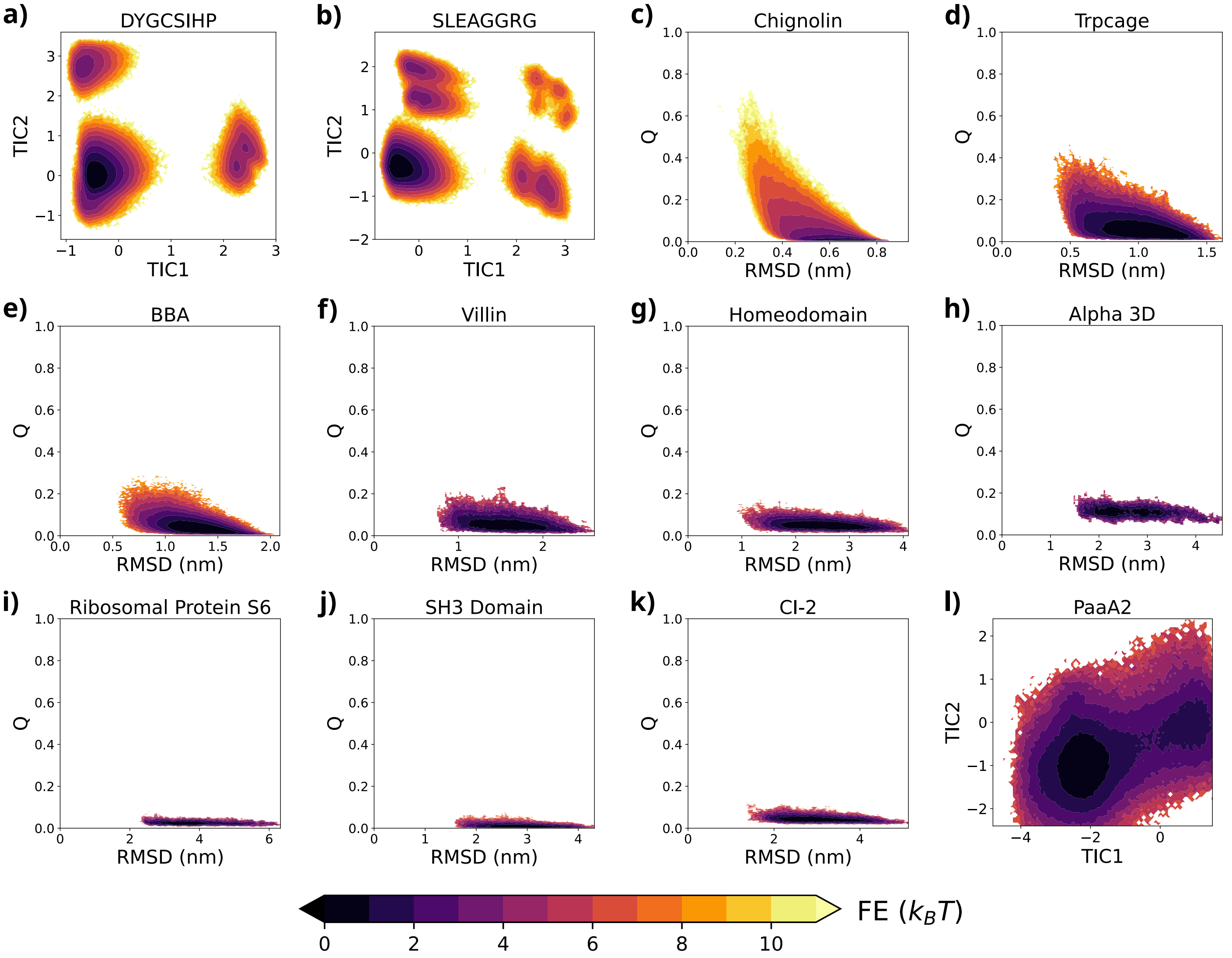

Fig. 7: Simulations of all proteins discussed in the main text from figures 2 - 4 performed only with a prior energy model, i.e. with no neural network. These proteins consist of a) 8-residue peptide DYGCSIHP, b) 8-residue peptide SLEAGGRG, c) CLN025 (2RVD), d) Trpcage (2JOF), e) BBA (1FME), f) Villin (1YRF), g) Homeodomain (1ENH), h) Alpha3D (2A3D), i) Ribosomal protein S6 (1RIS), j) SH3 domain (2NUZ), k) CI2 (2CI2), and l) antitoxin peptide PaaA2 (3ZBE). All free energy landscapes are shown as a function of RMSD and Q except for the two 8-peptides and PaaA2, where these coordinates cannot be easily defined due to the lack of unique folded states. Instead, the landscape is shown as a function of the first two TICA coordinates [16].

frames were used to train/validate the protein-specific model (striding of 5 from the full dataset). We applied the same constraint-aware force mapping strategy [29] as in the transferable dataset, and the same set of priors were fit directly to this dataset. We trained a version of the protein-specific model where we added decoys to the training dataset in the same way as it was done for the transferable model (see Supplementary Section 1.3) as well as a version without decoys. The resulting free energy surfaces obtained from both models align very well with the reference, as shown in Supplementary Fig. 8, thereby providing evidence that our prior strategy is reasonable for a delta-learning force approach. Additionally, it suggests that the network hyperparameters are expressive enough to model the multi-body interactions of the CG PMF (at least for a single protein). Despite its smaller number of frames, the protein-specific dataset contains information on Chignolin that is absent from the transferable dataset. The Chignolin-specific model has not only seen structures from all three metastable states during training, it has also seen transitions between them; this makes it easier for the model to learn the CG PMF and more correctly reproduce the overall landscape. Interestingly, when used to simulate a different protein, such as TRPcage, the protein specific model trained on Chignolin data rapidly unfolds initially folded structures and populates a misfolded basin reminiscent of the Chignolin native state, as shown in Supplementary Fig. 9.

None of this is true for the transferable model which has seen many frames from a dataset of high sequence diversity but with a rather low sequence and structural similarity to Chignolin. The very small difference between the presence or lack of decoys in the protein specific model further supports this claim. Indeed, while the decoys improve performance

of the transferable model (see Supplementary Fig. 21), in particular for stabilizing configurations outside the dataset, the marginal improvement of the protein-specific model is likely due to the presence of training data in most of the configurational space that can be visited by Chignolin.

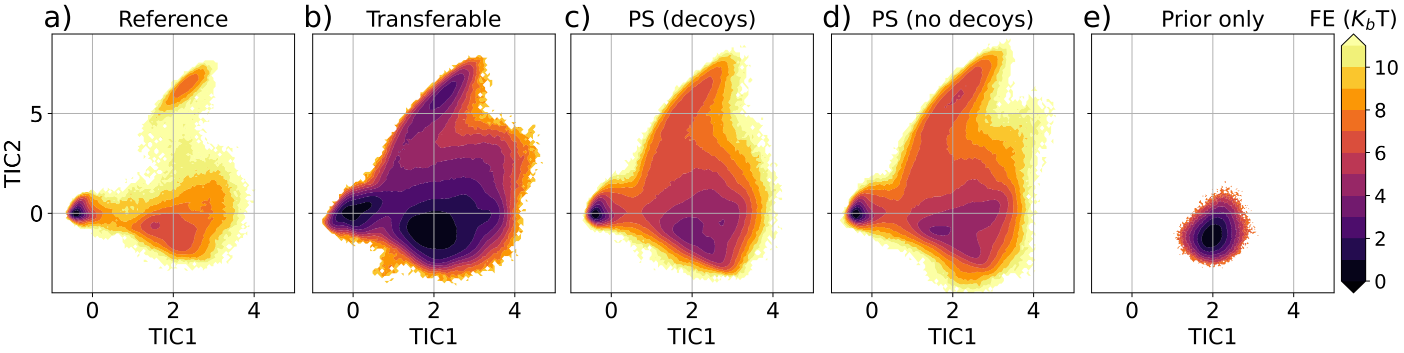

Fig. 8: Performance of a single protein model of Chignolin at the same resolution of the transferable model, illustrated by the free energy surface in the space of the first two components of a TICA analysis. a) All-atom reference, b) transferable model shown in the main text, reproduced here for comparison purposes, c) protein-specific (PS) CGSchNet trained on the AMBER Chignolin reference data with a prior energy and decoys, d) protein-specific CGSchNet trained with a prior energy but without decoys, e) simulation of the prior force field only.

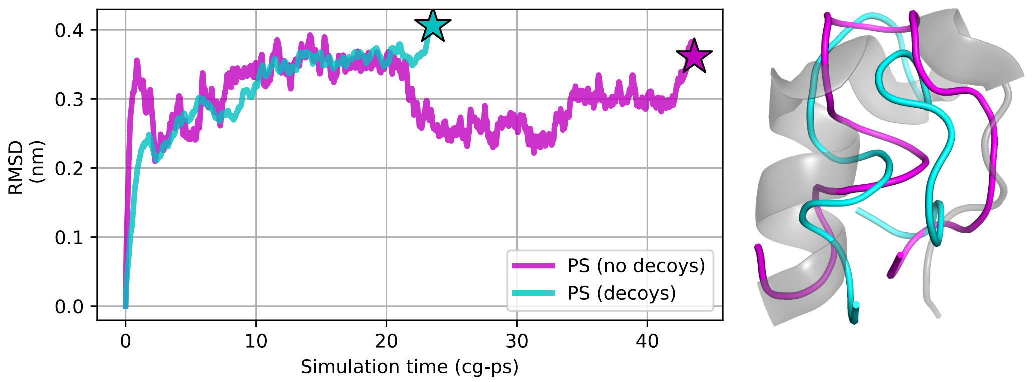

Fig. 9: Simulation of TRPcage with the protein specific Chignolin models of figure 8. (Left) C_α RMSD timeseries of two trajectories starting from the folded state of TRPcage. (Right) structures corresponding to the final frame of such simulations, with the folded starting structure in gray. In less than 40 ps, the simulations leave the folded state and populate a misfolded basin where parts of the molecular structure resemble the Chignolin folded state. After this simulation time, trajectories become unstable.

To assess the effect of the individual prior terms, we conducted a prior ablation study where several protein-specific models were trained by removing prior terms one by one. This study was done on a protein specific model rather than the transferable model to reduce the computational cost of generating delta-forces for each prior version and each individual frame of the training dataset. The results are shown in Supplementary Fig. 10. Unsurprisingly, a model trained without repulsion has a very rugged free energy surface containing many deep minima corresponding to structures where individual atoms are stuck together. Interestingly, the protein-specific model contains enough frames in low-populated regions to prevent too bad of an extrapolation (explosion) of a model without any prior. Also interesting is the fact that including all prior terms except an angle prior leads to a very unstable model that explodes after a few hundred steps. Further investigation of this observation may help to improve the performance of the transferable model in the future. Despite some models performing reasonably well, the model trained with the full set of prior terms performs significantly better than the others.

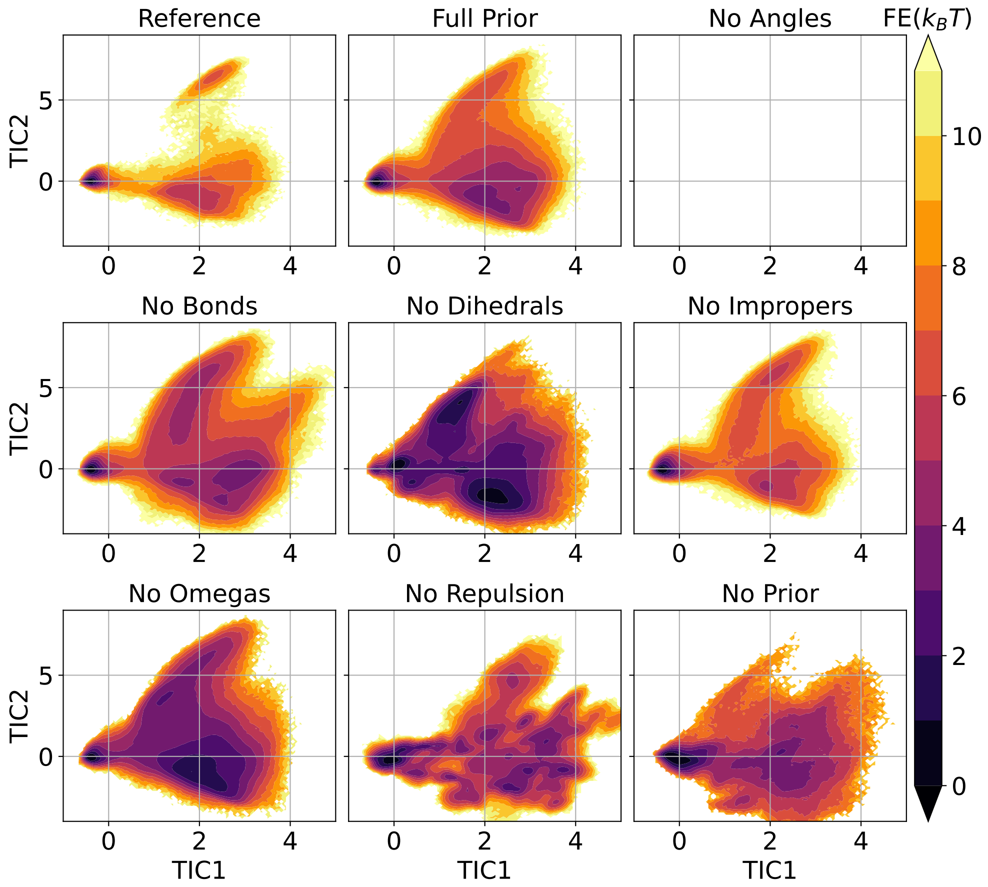

Fig. 10: Protein-specific models trained with different prior ablations. a) the all-atom reference on which all models were trained. b) the results of a model trained with the full prior used for the transferable model. c) to i) models trained selected prior terms removed. Note that the model trained without the a prior term for the angles in c) shows no data because the simulations systematically explode after a very small number of steps.

4 CG simulations

4.1 Langevin simulations

Following Husic, Charron, Lemm, et al [26], we performed Langevin simulations using the following BAOAB integration scheme:

$$
\begin{aligned}
(1:B) \qquad & \mathbf{v}_{t+1/2} = \mathbf{v}_t + \frac{dt}{2}\frac{\mathbf{F}(\mathbf{x}_t)}{m} \\
(2:A) \qquad & \mathbf{x}_{t+1/2} = \mathbf{x}_t + \frac{dt}{2}\mathbf{v}_{t+1/2} \\
(3:O) \qquad & \hat{\mathbf{v}}_{t+1/2} = \eta_v \mathbf{v}_{t+1/2} + \eta_n d\mathbf{W}_t \\
(4:A) \qquad & \mathbf{x}_{t+1} \quad = \mathbf{x}_{t+1/2} + \frac{dt}{2}\hat{\mathbf{v}}_{t+1/2} \\
(5:B) \qquad & \mathbf{v}_{t+1} \quad = \hat{\mathbf{v}}_{t+1/2} + \frac{dt}{2}\frac{\mathbf{F}(\mathbf{x}_{t+1})}{m}
\end{aligned}
$$

where $\mathbf{v}$ is the instantaneous velocity, dt is the integration timestep, $\mathbf{F}(\mathbf{x})_= - \nabla U(\mathbf{x})$ the force, $\eta_v = e^{-\gamma dt}$ and $\eta_n = \sqrt{K_B T(1-\eta_v^2)/m}$ are velocity and noise scales respectively, γ the friction parameter, and $d\mathbf{W}_t$ is a stochastic Wiener process. Table 5 contains the specific parameters used in all CGSchNet Langevin simulations. Multiple independent simulations were run on a single GPU in parallel for efficient sampling. The number of trajectories for each protein was chosen as the maximum number of trajectories that could fit on the GPU. For Chignolin, 50 folded and 50 unfolded configurations were used as starting configurations. For TRPcage, 35 and 35. For BBA, 25 and 25. For Villin, 5 and 5. For A3D, 5 from the folded state. For Homeodomain, 10 from the folded state. For the folding trajectories shown in Fig. 3 of the main text, 5 unfolded starting configurations were also used for Homeodomain and Alpha 3D. For the bigger proteins

shown in Fig. 4 of the main text (S6, SH3 and CI2), 5 trajectories were started from the crystal structure and for the IDP (3ZBE) 5 trajectories were started from conformations in the available NMR ensemble.

Integrator	Langevin
Friction (γ)	1 ps^{-1}
Timestep (dt)	4 fs
Temperature (T)	300K
Number of steps	(at least) 10^6
Masses (m)	Atomic

Table 5: CG Langevin simulation parameters

For the four smaller proteins (Chignolin, TRPcage, BBA, and Villin), the Langevin simulations were run for 4×10^6 steps at first, and convergence of the free energy was checked over time. If necessary, several more millions of MD steps were run until the landscape was converged. The initial 100,000 steps were always discarded for analysis to minimize the impact of the starting structure choices. For the larger proteins, for which we aimed to investigate stability and fluctuations near the native state, simulations were run for at least 4×10^6 MD steps (also throwing away the first 100,000 MD steps).

4.2 Parallel-tempering simulations

Following [27], parallel tempering (PT) simulations were performed in order to enhance the sampling. These simulations used the same integrator and integration settings as the Langevin simulations described above. The thermostat temperatures for different replicas range from below to above the target temperature (300K) and were selected as a geometrical progression with the possible exception of the target temperature, which was always included. For all simulations, an exchange between adjacent-temperature replicas is attempted every 2000 simulation steps. The number of replicas was adjusted so that the acceptance ratio of attempted exchanges between replicas with adjacent temperatures lies around 20% [30]. The number of replicas and temperature ranges specific to each simulated protein are reported in Supplementary Table 6.

The free energy surfaces were obtained by reweighting all the simulations to the target temperature through the MBAR procedure [31] as implemented in PyEmma [32]. The construction of the histogram required for the reweight was done using k-means clustering in the Q-RMSD space with 500 cluster centers.

For Chignolin, TRPcage, BBA, and Villin, the simulations were run for at least 2 million MD steps and extended as needed to achieve convergence in the landscape. For the other proteins, the PT simulations were run for at least 2 million steps. The analysis was performed after discarding the first 100,000 steps for all proteins.

Protein	Temperature range (K)	# replicas	# independent simulations
Chignolin (2RVD)	200-393.4	4	50
TRPcage (2JOF)	200-393.4	6	24
BBA (1FME)	200-393.4	6	18
Villin (1YRF)	200-406.8	8	4
Homeodomain (1ENH)	200-393.5	11	2
Alpha 3D (2A3D)	200-393.5	11	1
S6 (1RIS)	200-393.5	11	1
SH3 (2NUZ)	200-393.5	11	2
CI2 (2CI2)	200-393.5	11	2

Table 6: Parallel tempering parameters. Independent PT simulations were performed simultaneously to fully exploit the GPU capacity thus reducing the time spent on accumulating sufficient samples.

4.3 UNRES simulations

UNRES simulations of extrapolation targets were carried out using the UNRES web server [33, 34]. For each protein, several independent trajectories were run for 10 million MD steps each, using all of the default server settings and the latest version of the UNRES force field ("NEWCT-9P" [35]). For three of the four smaller proteins (Chignolin, TRPcage, BBA), 10 trajectories were started from the folded and 10 trajectories were started from the unfolded state. For every other target, 10 independent trajectories were started from the PDB crystal structure. No secondary structure, NMR, or SAXS restraints were used.

4.4 AWSEM simulations

AWSEM simulations were run using the AWSEM-MD code [36]. The simulation parameters used for these simulations are summarized in Supplementary Table 7. The total simulation time was set to a higher value to ensure correct sampling of the equilibrium NVT ensemble.

Integrator	Velocity-Verlet
Thermostat	Nose-Hoover
Number of Simulations	10
Damping	100 fs
Timestep	4 fs
Temperature	300 K
Number of steps	10×10^6

Table 7: AWSEM-MD simulation parameters

The Hamiltonian that was used for these simulations is the one as described in [36]:

$$V_{total} = V_{backbone} + V_{contact} + V_{burial} + V_{HB} + V_{FM} \tag{8}$$

To ensure comparability with our approach and assess the transferability of AWSEM to unseen proteins, only the homologs excluded database was used for the fragment memory term (V_{FM}), leaving roughly only sequences in the database that have less than 20% sequence similarity to the target protein. Secondary structure bias was used, but the secondary structure prediction was made using only knowledge of the primary sequence of the target protein to remain comparable with our approach. Secondary structure prediction was made using the JPred server [37] turning off the option to use a full structure from the PDB if existing.

4.5 MARTINI simulations

To assess the performance of our model against a widely used CG force field, we performed simulations of our four fast-folding targets, Chignolin (2RVD), TRPcage (2JOF), Villin (1YRF), and BBA (1FME), as well as two larger proteins, Homeodomain (1ENH) and alpha3D (2A3D) using the Martini 3 force field with explicit water. All input files for the simulations were prepared using CHARMM-GUI [38, 39], which prepares systems to be simulated in the NPT ensemble. All simulations were run with Gromacs 2021.3 [4]. Production simulations were run for 1 μs with a 20 fs time step, and for each system 5 simulations were run using the Velocity-Rescaling thermostat [40] and Parinello-Rahman barostat [41] at a simulation temperature and pressure of 300K and 1 bar, respectively. Non-bonded interactions were treated with the Verlet cut-off scheme, while a cut-off of 1.1 nm was used for van der Waals interactions and a dielectric constant of 15 and cut-off of 1.1 nm were used for Coulomb interactions.

Simulations of globular proteins with the Martini force fields are known to require an elastic network to preserve initial contacts formed in the folded state [42]. Our model, however, aims to explore the dynamics of a protein without the necessity for a known folded structure. Therefore, Martini simulations were run without the use of an elastic network to provide a fair comparison to our force field and other CG force fields evaluated here. To assess the performance of Martini 3 on stabilizing a known folded structure, simulations of Homeodomain and alpha3D were performed also using an elastic network and the same simulation parameters as described above.

In contrast to the other CG force fields discussed here, Martini CG mapping does not preserve the position of the carbon-alpha atom. Therefore, to accurately compare the performance of the different force fields, all Martini trajectories were back-mapped to all-atom structures using the Backward algorithm [43] from which only energy minimization was run and the equilibration steps were excluded.

5 Analysis details

In this section, we describe details on the simulation analysis presented in the main text.

5.1 Calculation of fraction of native contacts, Q

A common reaction coordinate for protein folding is the fraction of native contacts, Q, which characterizes the degree to which a given protein structure is similar to a known native structure. For a protein system with a set of native contacts distances, $\{r_0\}$, defined between a determined set of atom pairs, the value Q of a conformation with distances of the same

set of atom pairs $\{r\}$ is defined as:

$$Q(\{r\},\{r_0\}) = \frac{1}{|\{r_0\}|}\sum_{i}^{|\{r_0\}|} \frac{1}{1+\exp(\beta(r_i - \lambda r_{i,0})} \tag{9}$$

where the sum extends over all contacting pairs in the set $\{r_0\}$. The parameters β and λ control the contact membership smoothing and contact fluctuation allowance, respectively. This measure has been investigated and tuned for analysis of fast-folding proteins at the atomistic resolution [44].

To use this reaction coordinate for CG systems the following approach is taken. First, contacting residues (among residue pairs at least 3 residues apart in sequence) of an atomistic native state structure are defined if any two heavy atoms from either residue are within 4.5 Å of each other. Rather than recording these distances directly as elements of the set $\{r_0\}$, the distances of the corresponding C_α atoms of the contacting residues are recorded instead. After a set of C_α native contacts is identified, Eq. 9 can be used at the C_α resolution, which is applicable to any CG model with a resolution that retains or has knowledge of the C_α atom positions.

Additionally, λ and β must be adjusted, starting from their atomistically tuned values of 1.8 and 50 nm^{-1} respectively. Due to the higher positional rigidity of backbone carbon alpha atoms compared to sidechain atoms normally used to determine contacts in atomistic simulations, λ must be reduced. To this end, we set $\lambda = 1.5$, following the investigations of backbone-resolution CG Go-MARTINI protein models [45]. We set the parameter $\beta = 10$ nm^{-1} to obtain a smooth sigmoid for the Q function. This set of λ and β allows for interpretable and smooth free energy surfaces that can be employed for CG analysis of any protein in this study, all of which clearly separate native/native-like configurations from unfolded configurations (i.e., $Q \approx 1$ for configurations close to the native structure).

5.2 Calculation of the root mean square fluctuations

For the computation of the C_α Root Mean Square Fluctuations (RMSF) inside the folded state shown in Fig. 3, one trajectory starting from the folded state was chosen for each protein. A window of frames maintaining the folded state for at least 1 million MD steps was chosen to compute the C_α RMSF. The chosen trajectories and windows for both Homeodomain and alpha3D are shown in Supplementary Fig. 11.

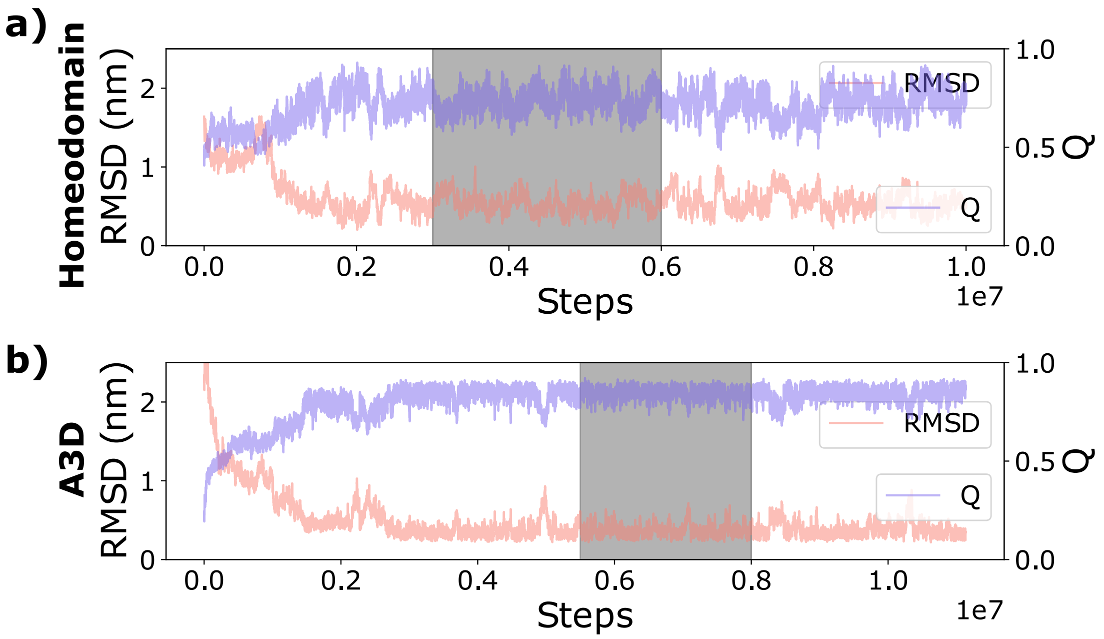

Fig. 11: Trajectory windows used for the computation of the C_α Root Mean Square Fluctuations for Homeodomain (1ENH) and alpha3D (2A3D).

5.3 Extrapolation target sequence similarity

We define the sequence similarity, o, for a target sequence S_T and a reference sequence S_R as $o = (|\text{Align}(S_T, S_R)|)/(\text{length}(S_T))$ where "Align" is a lexicographic alignment function that returns a scalar score. For alignments, we use Biopython's `pairwise2.align.localxs` function [46], with gap open and extend penalties both set to

−1. The alignments with the highest score are used. Table 1 in the main text shows the maximum sequence similarity (rounded to the nearest whole percent) with the CATH domains for each extrapolation target used in this work. All 50 CATH domains were considered in this analysis.

5.4 Computation of the structural metrics

5.4.1 Structural metrics

In Supplementary Fig. 12, we quantify the ability of the different force fields to populate and stabilize the native state structure, as measured by two metrics: the average GDT-TS scores [47] with respect to the corresponding reference folded structure, and the average Q values of the folded state for the set of fast-folding proteins considered in the main text. This provides a quantitative comparison between the folded states obtained with the CG models considered and an all-atom model (DESRES [17]). As an additional reference, we add the scores obtained by AlphaFold3 [48] predictions. We note, however, that for the CG models and for the all-atom DESRES model, structures are selected from a metastable basin as described in Supplementary Section 5.4.3. Thus, the metrics shown in Supplementary Fig. 12 illustrate the ability of the model to stabilize a metastable state close to the native structure. AlphaFold only predicts native structures rather than an entire FES and the associated metastable states, and therefore represents more of a theoretical goal to achieve for the force-fields than an actual comparison.

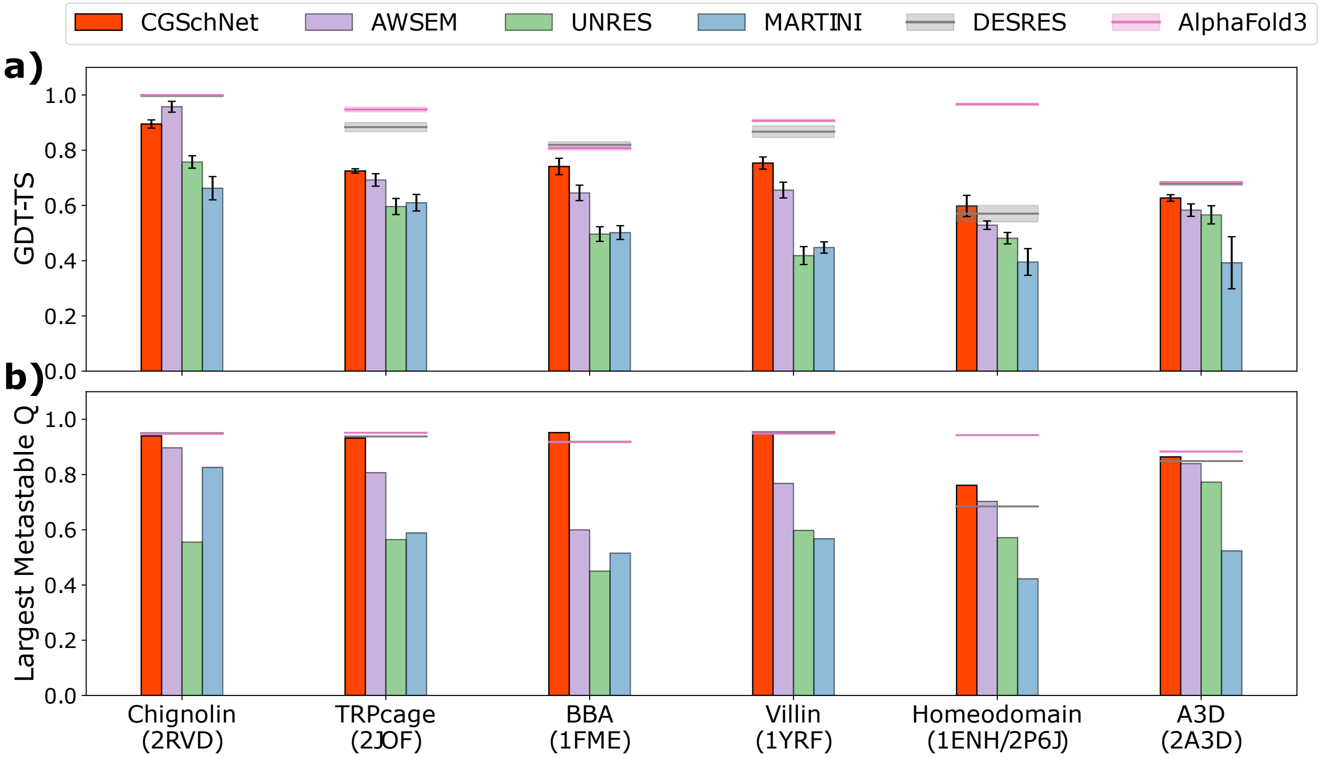

Fig. 12: Comparison of transferable CGSchNet against three other CG force fields of a comparable resolution, AWSEM, UNRES, and Martini, as assessed by the ability to stabilize structures near known native states. None of the proteins shown were used to parametrize the transferable CGSchNet. The upper lines on each plot show the comparison to the all-atom DESRES simulations [17] as well as to the Alphafold3 [48] prediction. a) Average GDT total scores for several proteins over 10 random structures. Representative structures were sampled from the most folded-like metastable state on the 2D Q vs. RMSD free energy surfaces of each CG force field shown in Fig. 4 (see Supplementary Section 5.4.3 for details). Error bars correspond to standard deviations in the scores across the 10 structures. b) Highest local maxima recovered from the 1D Q probability distribution for each CG force field over all proteins. Note that for Homeodomain, the DESRES simulations use a slightly different sequence than in our study, corresponding to the PDB code 2P6J [49].

5.4.2 GDT-TS score

The Global Distance Test (GDT) [50] was introduced as a more refined alternative to RMSD measurements for protein structure comparisons, being less sensitive to disordered regions. For a chosen distance cutoff, r_{GDT}, the GDT consists of finding the largest set of (potentially non-contiguous) matching protein residues between a query and a target structure whose corresponding C_α pair distances differ by no more than r_{GDT}. The GDT algorithm converges to a static set of maximally superimposable residues, and the output is given as a percentage of the total number of residues in the target structure. The GDT-TS ("Global Distance Test Total Score") is reported as the average percentage of maximally superimposeable residues from running the GDT algorithm for multiple r_{GDT} choices. The CASP3 structure prediction competition officially adopted the GDT-TS as a measure of how accurately a model-generated query structure matched a target structure for $r_{GDT} = 1, 2, 4, 8$ Å cutoffs [47]. As the GDT-TS only requires knowledge of query and target structures' carbon alpha atom positions, it can be used without modification for any CG model that retains knowledge of these atoms. In all GDT-TS measurements for any CG model in this manuscript, the default cutoff series ($r_{GDT} = 1, 2, 4, 8$ $\mathring{A}$) is used. All GDT-TS computations were done using the Zhang lab C++ standalone implementation of the TM-score program [51].

5.4.3 Extraction of structure ensembles

For each protein and each model, a free energy surface was plotted in Q vs. RMSD space (or as a function of the two first TICA components [16] in the case of Fig. 4) excluding the first 10% of the simulation for AWSEM and UNRES simulations and the first 100,000 MD steps for the CGSchNet simulations. This free energy surface was clustered using k-means clustering and the number of clusters shown in Supplementary Table 2 for Fig. 4 or 100 cluster centers for the computation of the structural metrics in Supplementary Fig. 12. For Fig. 4, the cluster best representing the minima of interesting basins (i.e. the most folded-like and other prominent metastable basins) was chosen by visual inspection and 10 structures were randomly sampled from it. For computation of the GDT-TS scores in Supplementary Fig. 12a, structures were randomly sampled from the cluster center near the most folded-like metastable basins in RMSD vs. Q space (i.e., the free energy minima with simultaneously the lowest RMSD and highest Q). These cluster centers are indicated for each protein and each force field in Supplementary Fig. 13. It is to be noted that the most folded-like metastable basin does not always correspond to the most populated cluster, neither in our model nor in the other CG models presented. As it is illustrated in Supplementary Fig. 13, this criterion allows a fair comparison between our model and the other CG models in the choice of the most folded-like metastable basin. The structures pulled for GDT-TS are aligned with the reference and visualized in Supplementary Fig. 14.

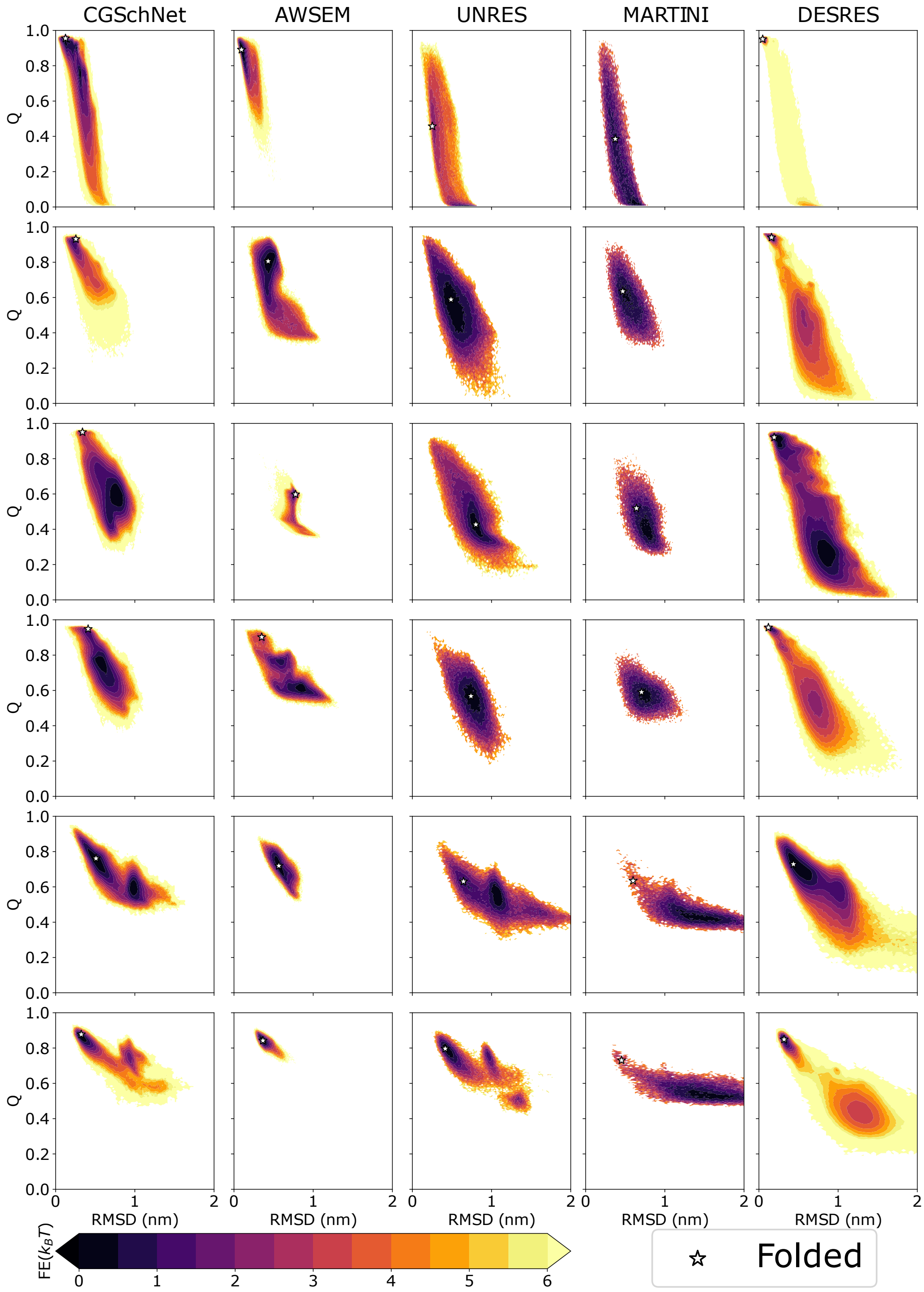

Fig. 13: Details on the computation of the structural metrics shown in Supplementary Figure 12. For each protein and each CG force field, the free energy surface in Q vs. RMSD space and the most folded-like cluster center, from which structures for the computation of the GDT-TS score were extracted, are plotted.

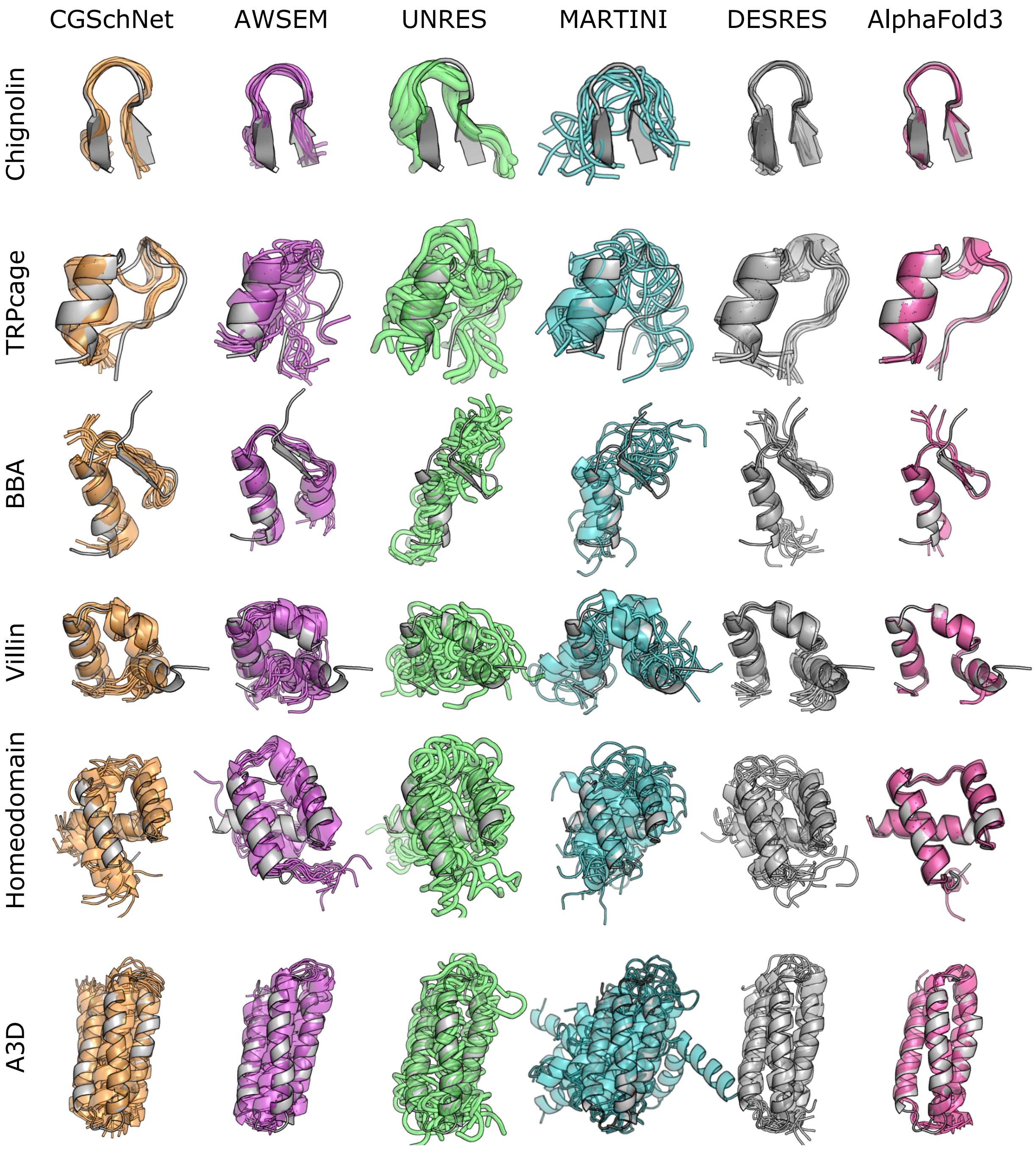

Fig. 14: Ensemble of structures pulled for the computation of the GDT-TS score for each protein and each CG force-field, as well as the structures pulled from the DESRES simulations [17] and the structures obtained with AlphaFold3 [48]. Subfigures generated using PyMol [7].

5.4.4 Computation of the highest metastable Q

For the computation of highest metastable Q scores in Supplementary Fig. 12b, we adopted the following procedure: The probability density of the fraction of native contacts (Q) was computed for the same part of the simulations as in Supplementary Fig. 12a, and a smoothed version was plotted using a Savitzky-Golay filter of order 10 (5 for 1ENH) [28, 52]. The order was chosen to give consistent results on all simulations of the same protein, minimizing the smoothing artifacts and making sure that the remaining artifacts did not impact the detection of the right peak. The relative maxima of the smoothed Q probability density were computed using SciPy and the highest Q value corresponding to a relative maximum in probability density was taken as the largest metastable Q.

5.5 Details on the MCL-1/PUMA simulations

The unfolded ligand was obtained by taking a folded PUMA conformation from the reference NMR structure of the folded MCL-1/PUMA complex (PDB code 2ROC [53]) and running high temperature (500K) atomistic NPT and NVT simulations using the AMBER ff-99SB-ILDN force-field [5]. A rather disordered conformation (coil fraction fraction 0.85) was then chosen from these simulations as an initial disordered structure for PUMA that was either simulated alone using our CG model, or placed next to the folded proteins MCL-1 and Ubiquitin before CG simulation. As a comparison, the coil fraction of the folded PUMA peptide from the NMR structure is 0.19.

For the CG simulations of the complexes, the disordered peptide was placed close to the binding pocket of the folded MCL-1 protein from the 2ROC structure and next to the folded Ubiquitin structure from PDB entry 1D3Z [54] using PyMOL [7]. Supplementary Table 8 summarizes the initial center of mass distance and closest contact for both complexes to ensure a fair comparison.

	**MCL1+PUMA**				**UBQ+PUMA**		
	Ref. folded	Init.	Average	Min.	Init.	Average	Min.
PUMA Rg (nm)	1.19	0.97	1.17	0.76	0.97	1.04	0.71
PUMA helix fraction	0.81	0.15	0.75	N.A.	0.15	0.58	N.A.
PUMA coil fraction	0.19	0.85	0.25	N.A.	0.85	0.41	N.A.
COM dist. (nm)	1.51	2.74	1.76	0.96	2.35	1.68	1.30
Closest contact (nm)	0.33	0.48	0.36	0.23	0.38	0.28	0.22

Table 8: Characteristics of the disordered PUMA peptide and the complexes formed with folded MCL-1 and Ubiquitin at CG resolution. The leftmost column contains the reference measurements for the folded MCL-1+PUMA complex from PDB entry 2ROC. For each complex, the initial, average and minimum measurement is then shown. The three first measures (radius of gyration, helix and coil fraction) are done on the PUMA peptide alone and the last two (center of mass distance and closest contact distance at the CG resolution) involve the entire complex. Fig. 15 shows the time series of the coil and helix fraction in the simulations of PUMA with MCL-1 and UBQ.

Coarse-grained simulations were then run similarly to all other systems presented in this work, using Langevin simulations at 300K without any additional constraints on the folded proteins. The results are presented in Fig. 5 and Supplementary Fig. 15.

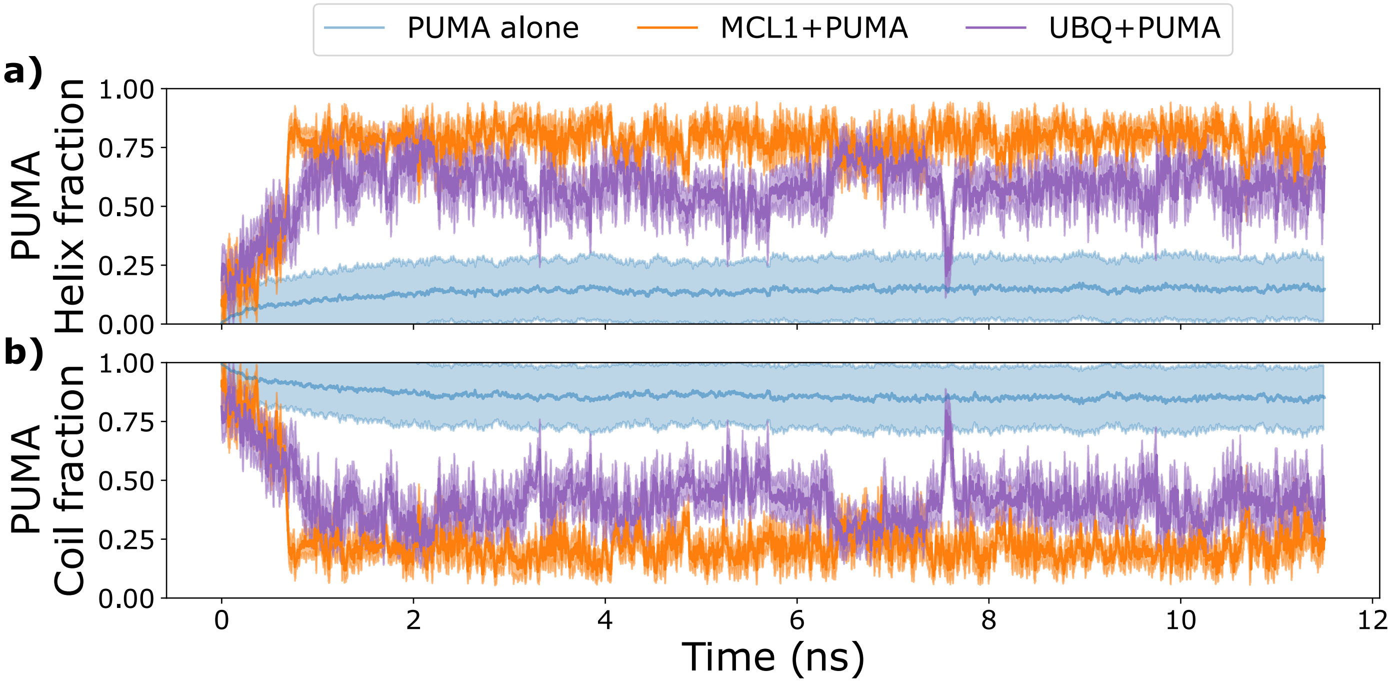

Fig. 15: Helix (a) and coil (b) fractions in the PUMA peptide during simulation alone (blue), in complex with MCL-1 (orange) and in complex with Ubiquitin (purple). For the two complexes, a rolling mean over 250 frames from the trajectories shown in Fig. 5 of the main text are shown with corresponding standard deviation. For the PUMA peptide alone, the mean and standard deviation are additionally taken over the 50 trajectories simulated, resulting in a smoother curve.

5.6 Details on the Ubiquitin mutational study

Following previous work [55, 56], under the assumption that a mutation does not significantly perturb the density of states of the conformational ensemble, the effect of the mutation on the protein stability can be estimated from perturbation theory as:

$$\beta \Delta\Delta G = ln \frac{\langle \exp(-\beta \delta E) \rangle_U}{\langle \exp(-\beta \delta E) \rangle_F} \tag{10}$$

where $\delta E = E_{mutant} - E_{WT}$ and the angular brackets represent the average over the folded (F) or unfolded (U) state ensembles of the unperturbed (WT) system.

For the $\Delta\Delta G$ estimation shown in Fig. 6 of the main text, we extract 100000 folded and 100000 unfolded frames from MBAR-reweighted PT simulations of Ubiquitin (PDB code 1D3Z [54]) with our transferable CG model, shown in Supplementary Fig. 5.6a. We define a range of RMSD and Q values for the folded and unfolded states and use the reweighted probabilities to extract frames in these two regions. The density plot of the extracted folded and unfolded frames is shown in Supplementary Fig. 5.6b.

On each of these frames, we change the identity of the bead representing the C_β (or remove it and change the C_α identity in case of mutations to GLY) to mimic the effect of a point mutation on the structure. Note that the WT used in [57] has the mutation F45W compared to the original 1D3Z chain, and we use F45W as the wild type in our study and add the other mutations on top of it. For this wild type and for each mutation, we then compute the CGSchNet energy on each folded and unfolded configuration and use these values to estimate $\Delta\Delta G$ according to Eq. 10. The results are shown in Fig. 6b and Supplementary Fig. 16c. Error bars are estimated via bootstrapping, resampling 99 times by taking batches of 10000 independent elements of the initial folded and unfolded ensemble and estimating the standard deviation of $\Delta\Delta G$ values obtained.

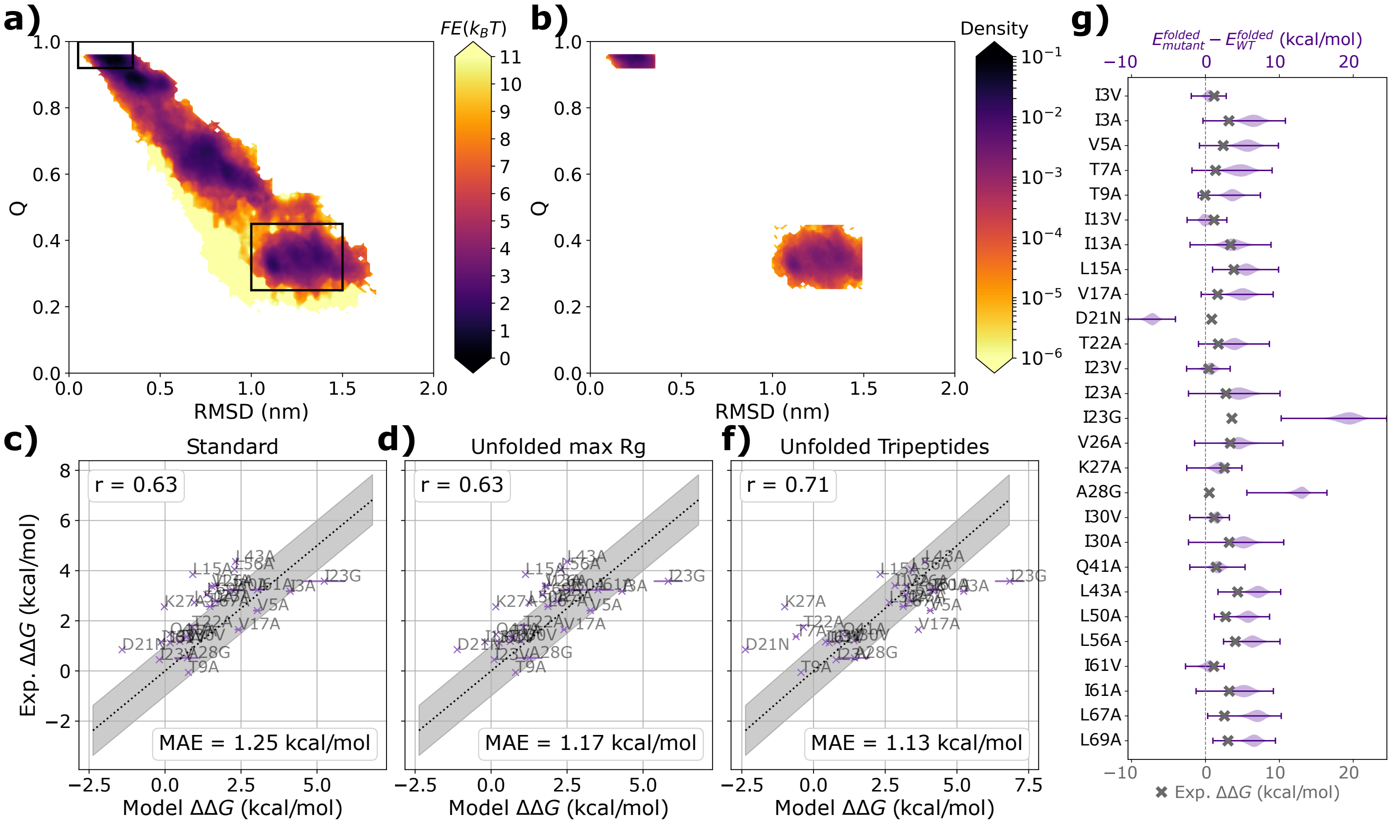

Fig. 16: Mutational study on Ubiquitin. a) shows the MBAR-reweighted free-energy surface obtained with PT simulations of Ubiquitin (PDB ID 1D3Z), b) shows the density plot of the extracted folded and unfolded ensembles used for $\Delta\Delta G$ computation. c), d) and f) show $\Delta\Delta G$ computations with three different unfolded ensembles described underneath, compared with experimental $\Delta\Delta G$s [57] and g) shows the energy difference between a mutant and a WT structure inside the folded state compared to the experimental $\Delta\Delta G$. The violin plot represents the entire range of distribution of energy differences for the 100000 structures in the folded ensemble, with bars at the extrema of the distribution. Error bars in c), d) and f) were estimated via bootstrapping, resampling 99 times by taking batches of 10000 independent elements of the initial folded and unfolded ensembles and estimating the standard deviation of $\Delta\Delta G$ values obtained for each mutation

A challenge in the computation of $\Delta\Delta G$ values with this approach with atomistic simulation is usually the definition of the unfolded state [58]. While using PT simulations should allow us to sample the equilibrium unfolded state of Ubiquitin as defined by our model, we observe a lot of residual secondary structure in the unfolded ensemble. To showcase the impact of the definition of the unfolded ensemble, we perform two modified $\Delta\Delta G$ estimation. In Supplementary Fig. 5.6d we show the $\Delta\Delta G$ estimation when using only the 20000 unfolded frames selected to have the highest radius of gyration from our original unfolded ensemble, to artificially decrease the amount of residual structure. This improves the mean average error slightly. In Supplementary Fig. 16f we show a $\Delta\Delta G$ computed using an approach inspired by common practices in alchemical calculations [58], where tripeptides are used as an approximation for estimating the effect of a mutation in the unfolded state. We performed simulations of GXG tripeptides (with X being a WT residue we wish to mutate) with our transferable CG model to sample 800000 structures for each tripeptide. For each mutation X2Y we then considered the structures from GXG as WT unfolded structures and mutated the central amino-acid to obtained an estimate of δE to insert in Eq. 10. The results are shown in Supplementary Fig. 16f. Note that this approach slightly improves the Pearson correlation and the mean average error. Finally, we analyze how the change in the energy predictions of the CG model in the folded state compare with experimental $\Delta\Delta G$ values, effectively using a first order approximation that assumes no effect in the unfolded state. On Supplementary Fig. 16g, we show that the energy difference between mutant and WT folded states (shown in the purple distributions) are in most cases close the experimental $\Delta\Delta G$ value. Exceptions are the GLY mutations, where the entropic effect of removing a bead is not properly accounted by merely computing $E^{folded}_{mutant} - E^{folded}_{WT}$, and the D21N mutation, which is the only mutation in the set that doesn't conserve charge.

6 Additional analysis

In this section, we present additional analysis not described in the main text, but relevant to an interested reader.

6.1 Additional systems

As an additional test, we examine the extrapolative performance of our CG model on other large proteins for which we only have experimental reference data: we examine the native state stability of three proteins with known PDB structure, the 97-residue ribosomal protein S6, a 55-residue SH3 domain, and 65-residue CI-2 domain, and the conformational heterogeneity of the partially disordered 71-residue antitoxin peptide PaaA2 for which helical fraction content is known from an ensemble of NMR structures. The results are illustrated in Supplementary Fig. 17.

For the three structured proteins, we see that the CG simulations stabilize structures near the folded state (Supplementary Fig. 17a-c) while also sampling alternative states: for S6, a metastable state is visited in which residues 36 and 44 undergo a sheet to helix rearrangement, while maintaining tertiary structure (Supplementary Fig. 17a).

While PaaA2 does not have a unique experimentally determined structure, an NMR ensemble of 50 structures shows that two short helical domains are formed with disordered linkers and termini. The CG model qualitatively predicts the same equilibrium helix fraction per residue as the NMR ensemble, although it overstructures the N-terminus (Supplementary Fig. 17d). In addition, the 50 NMR structures of PaaA2 are all located in the main two metastable states of the free energy landscape obtained from the CG simulations (labeled as states 4 and 5 in Supplementary Fig. 17d), while the model also explores additional, albeit shallower, metastable states (labeled as 1, 2, 3 in Supplementary Fig. 17d). All-atom simulations initialized from structures in these additional states remain within the corresponding states for at least 1 μs, indicating that the additional ensembles may be either too unstable to be experimentally detected, or inherited artifacts from the reference all-atom force field rather than spurious products of the CG model.

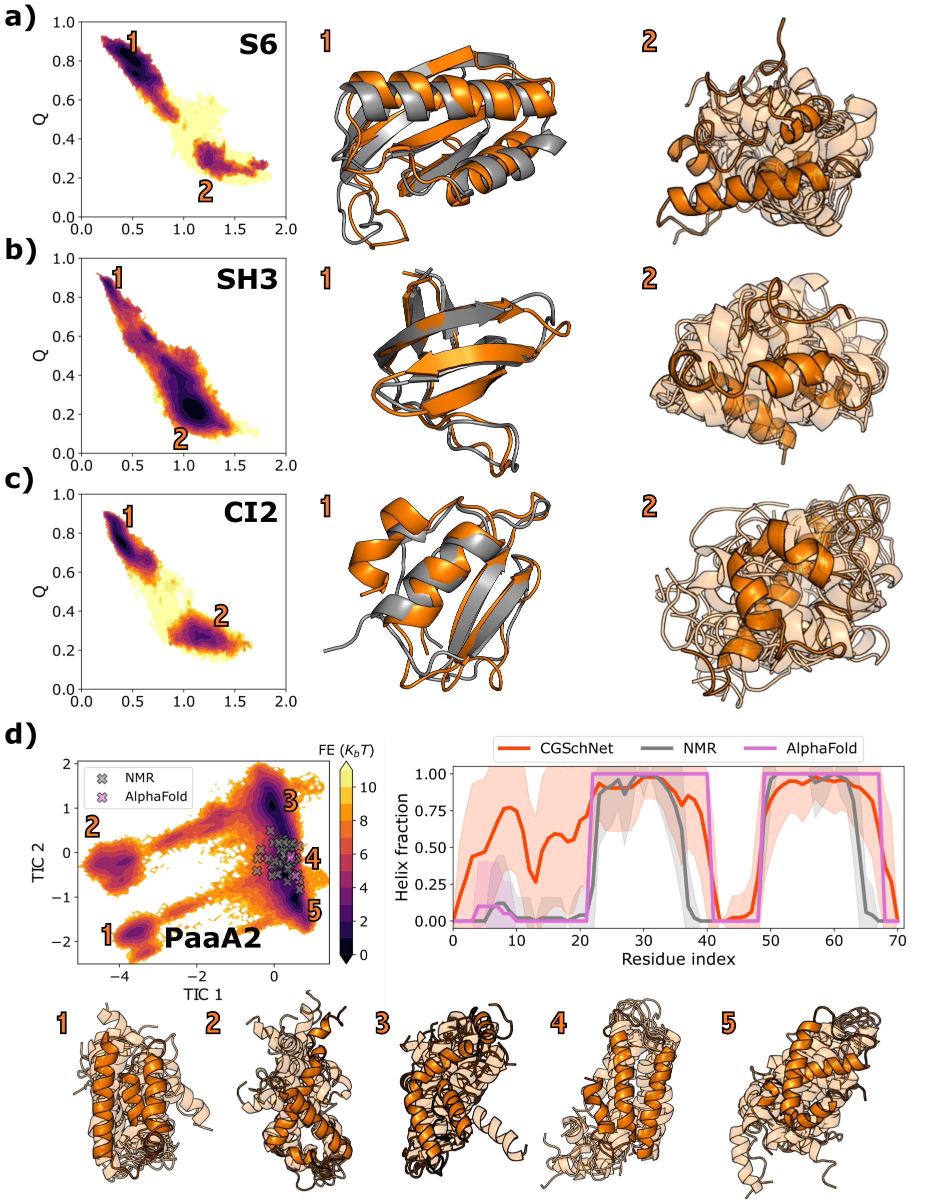

Fig. 17: Performance of the transferable CGSchNet on four large proteins outside of the training and validation sets for which there is no reference all-atom simulation data: a) 97-residue ribosomal protein S6 (1RIS), b) 55-residue SH3 domain (2NUZ), c) 65-residue CI-2 (2CI2) and d) Intrinsically disordered 71-residue antitoxin peptide PaaA2 (3ZBE). a-c, left: 2D free energy landscape as a function of the fraction of native contacts, Q, and the C_α RMSD to the native state, obtained through MBAR-reweighted [31] PT simulations; middle: structures from the most folded-like metastable basin for CGSchNet (orange) and crystal structure (grey); right: illustrative structures extracted from the metastable basins indicated by the label. d, left: Free energy surface of PaaA2 as a function of the two first TICA components [16], obtained through CG Langevin simulations, with structures from the NMR ensemble and from the AlphaFold prediction marked with an x. Right: helix fraction per residue resulting from the DSSP [2] analysis on our CG simulations, the NMR ensemble, and the AlphaFold prediction. Thick lines represent the mean and shaded regions represent the standard deviation of DSSP assignment for each residue over the respective ensembles (1.25e6 structures from the CG simulation, 50 NMR structures, 20 AlphaFold structures) Bottom: Representative structures extracted from different metastable states.

6.2 Computational efficiency of CG simulations

We compare the computational efficiency between our CG model (CGSchNet) and the reference all-atom simulations. While a single simulation timestep with run the neural network model in PyTorch takes more wall-clock time than a single all-atom timestep in OpenMM [59] using the same GPU (see Supplementary Table 9, this occurs because the all-atom potential is much simpler than our highly expressive machine-learned CG model. Furthermore, MD simulation software, such as OpenMM, is highly optimized for speed, whereas deep learning packages such as PyTorch are optimized for

parallel processing. Despite this "slowdown" per step, one CG simulation step corresponds to a much longer physical time equivalent. Two factors compound here: first, the CG representation is limited to a few beads with effective interactions, allowing the physical time step length to be larger than in an all-atom simulation. Second, the CG landscapes appear smoother overall than their atomistic counterparts as a result of averaging over subsets of the all-atom degrees of freedom, with lower energy barriers separating folded and unfolded states . Thus, our CG model is overall more efficient than the atomistic reference.

To quantify the effective speedup from the CG model, we estimate the wall-clock time required by both the CG and the all-atom models to obtain one uncorrelated sample for the four small folders in Fig. 2. This is measured in terms of the slowest relaxation timescale of a Markov state model [15, 60], which is typically due to the dominant folding/unfolding transition for small folders. The speed-up factor can be decomposed into two parts: (1) The decorrelation time, or slowest relaxation time, τ is the value of the implied timescale of the slowest process according to an MSM built over the trajectories of the simulation. For the systems described here, the slowest process is typically the folding/unfolding transition; (2) The "speed of simulation" v measures the computational cost of one simulation step on the same reference GPU. We can quantify the speed-up with the following formula:

$$s = \frac{\tau^{(AA)}}{\tau^{(CG)}} \frac{v^{(CG)}}{v^{(AA)}} \tag{11}$$

where AA and CG stand for all-atom and coarse-grained, respectively.

The CG simulation times were estimated from Langevin simulations with the temperature set to the same as the corresponding AA simulations (300K). Parallel tempering (PT) simulations are not considered here because the transitions are partially due to temperature swaps and are therefore not used to infer the physical transition time. The results of the speed-up calculation can be found in Table 9.

Protein	$\tau^{(AA)}$ (TS_{AA})	$\tau^{(CG)}$ (TS_{CG})	$v^{(AA)}$ (TS_{AA}/s)	$v^{(CG)}$ (TS_{CG}/s)	Speed up
CLN025	$2.262(4) \times 10^8$	$7.400(5) \times 10^4$	4.080×10^3	57.10	42.77
Trpcage	$1.694(6) \times 10^9$	$3.165(4) \times 10^4$	2.488×10^3	53.20	1144
BBA	$9.655(9) \times 10^8$	$9.89(1) \times 10^4$	2.488×10^3	55.60	218.0
Villin	$1.488(0) \times 10^{10}$	$7.947(5) \times 10^5$	4.051×10^3	52.40	242.1

Table 9: Speed-up calculation. Times for the AA simulations are presented in AA timestep (TS) = 2 fs. The simulation speeds v were measured on a single Nvidia A5000 GPU. The timescales τ reported are the mean value (and standard deviation as uncertainty) of a set of 10 independent estimations.

The reported CG simulation speed $v^{(CG)}$ is not affected by molecular size, since the wall time spent for each time step is dominated by the hardware communication overhead. In practice, multiple simulations can be executed in parallel to saturate the computation capacity of a GPU by amortizing the overhead of hardware communication, which usually brings 5-50x throughput (see Supplementary Table 10). It is also worth noting that this speed-up is achieved even though the MD simulations of our CG model were performed with our in-house code, which is not yet optimized for performance. The MD simulations for the reference atomistic model were performed with OpenMM, which is highly optimized. In general, MD simulations with neural network potentials are still very expensive compared to classical force fields.

Protein	$v^{(CG)}_{\text{batched}}$ (TS_{CG}/s)	Parallel trajectories	$v^{(CG)}_{\text{batched}}/v^{(CG)}$
CLN025	2.92×10^3	100	51.02
Trpcage	7.95×10^2	70	14.94
BBA	3.70×10^2	50	6.66
Villin	3.27×10^2	40	6.25

Table 10: GPU batching speed-up. The number of parallel trajectories was selected to maximize the usage of GPU memory. The simulation speeds v were measured on a single Nvidia A5000 GPU

Depending on the protein, the CG model is between a factor of 30 and 300 faster than all-atom simulations on the same GPU (see Supplementary Table 9 in the SI). Besides this speedup, our CG models have another substantial efficiency gain in terms of throughput: while OpenMM is designed to run one simulation per GPU, multiple simulations can effectively be run in parallel on a single processing unit with the PyTorch package with negligible increase in walltime. For the proteins shown here, this results in an *additional* 5× to 50× increase in simulation throughput (see Table 10). We note

that, in principle, a substantial throughput increase might also be possible for all-atom simulation software if their codes were designed for batch simulation.

6.3 Comparison to previous machine-learned CG models

In order to clarify the difference between the transferable model presented here and previous machine-learned CG models trained with a similar method, such as the one in [18], we perform simulations for three globular proteins with no available reference MD data. The model presented in [18] (hereafter called the interpolative model) is not a transferable model and was aimed to show the possibility of *interpolating* in sequence space, training a single model capable of simulating the same sequences that were present in the training set. The CGSchNet model presented in the current study was designed to be able to *extrapolate* to new sequences with very little similarity to those systems in the training set, making it more widely usable. To illustrate this difference, we simulate three such dissimilar sequences that have never been seen by either model during training.

The simulations with the interpolative model were performed using Langevin dynamics at the temperature of 300K with a friction coefficient of 1 ps^{-1}. Due to stability reasons, the interpolative model requires a timestep of 1 fs for the simulation. As it can be seen from Supplementary Fig. 18, our transferable model outperforms the interpolative model,

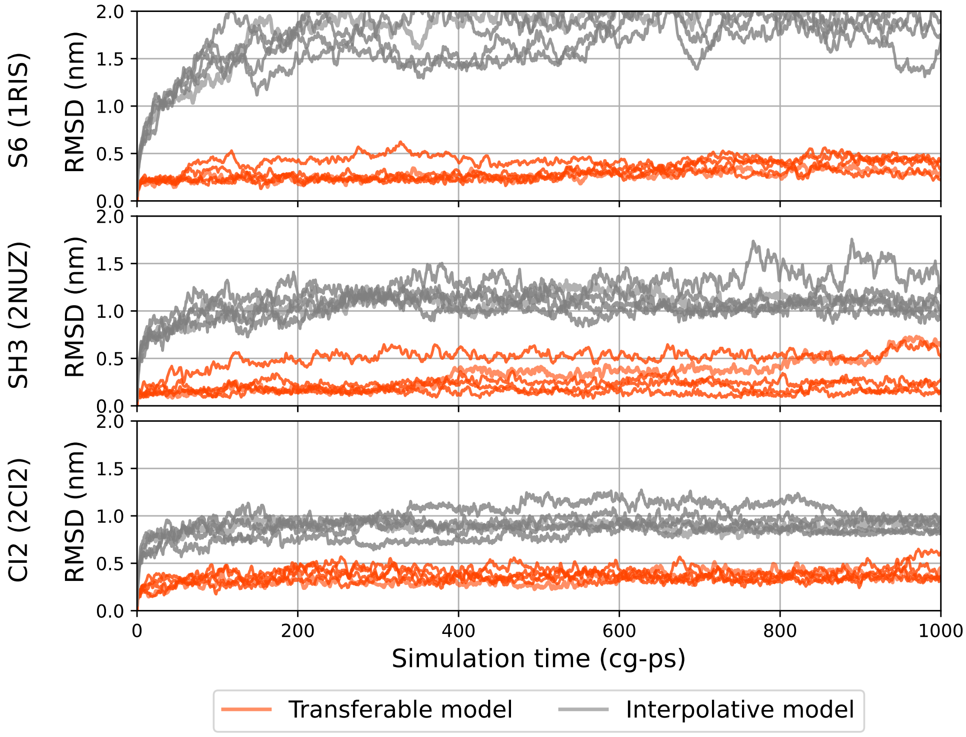

Fig. 18: Timeseries of RMSD as function of simulation time for 1RIS, 2NUZ and 2CI2. Different curves show independent trajectories starting from the same folded state.

as it able to stabilize the folded state (consistent with Supplementary Fig. 17) while the interpolative model is unable to do so and immediately loses the tertiary structure of the system.

6.4 Comparison between Langevin and parallel-tempering simulations

To demonstrate the stability of our model under various simulation setups and techniques, we present a comparison of the free energy landscape obtained through Langevin and parallel-tempering (PT) simulations in Supplementary Fig. 19. As in the main text, the free energy is reported as a function of the fraction of native contact and C_α RMSD to the native state.

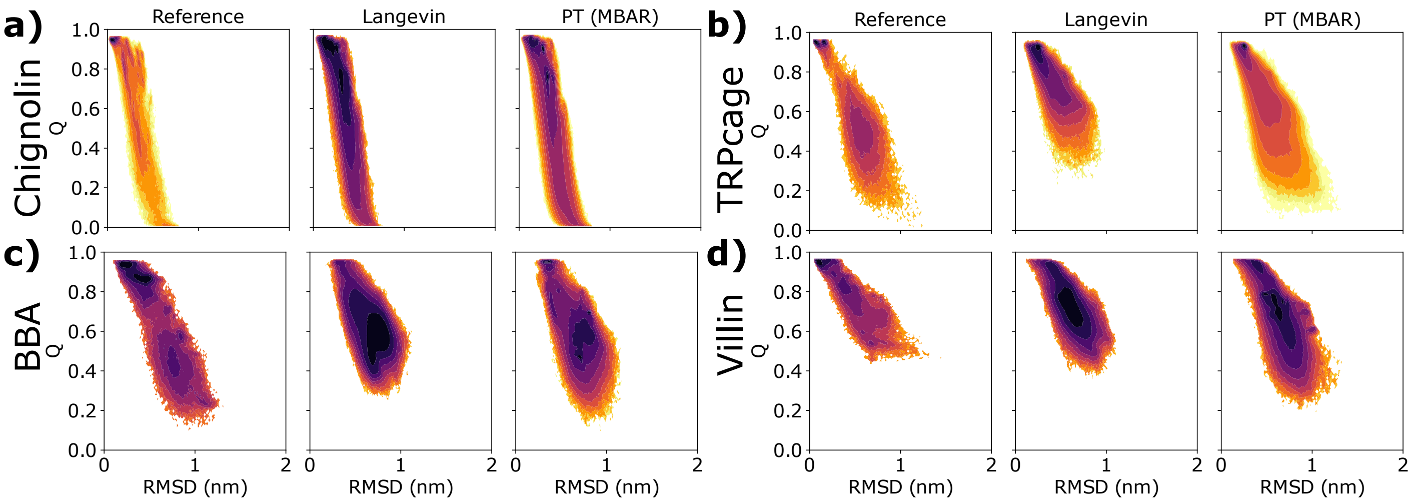

Fig. 19: Comparison of parallel-tempering (PT) and Langevin simulations for the four small proteins shown in the main text: a) Chignolin (2RVD), b) TRPcage (2JOF), c) BBA (1FME), and d) Villin (1YRF). Results from the PT simulations are MBAR-reweighted [31].

6.5 Dataset ablation study

The training dataset used to achieve our final model was carefully constructed to contain a significant diversity both in sequence and structure. To highlight the importance of the different components of our dataset, we conducted an ablation study by training a model on various subsets of the dataset, and evaluated these trained models on our four fast-folding targets, namely Chignolin, TRPcage, Villin, and BBA (Supplementary Fig. 20). All models in the ablation study are trained to a epoch comparable to our most successful model presented in the main text. Both models trained with a smaller training subset (respecting the composition of the whole dataset) demonstrate reasonable exploration of the free energy surface but are not able to stabilize the folded state of any of the targets, despite having the same sequence diversity as our main model. As expected, using only 10% of our training data yields significantly worse performance than using 50%. Training with only the CATH proteins while withholding the dimer dataset produces a model that is able to fold the primarily helical targets, TRPcage and Villin, but struggles to stabilize the folded states of Chignolin and BBA. If, however, this dataset is further reduced to contain only primarily helical or sheet proteins, the model's performance on all targets degenerates. Finally, withholding the CATH proteins and training only on the dimers produces the worst model of this ablation study, yielding results similar to the prior-only simulation (shown in Supplementary Fig. 7). This demonstrates that while the dimer dataset is an important addition to the training data in terms of adding residues interacting at short distances, it does not contain enough information about the multi-body terms that are required for a successful model.

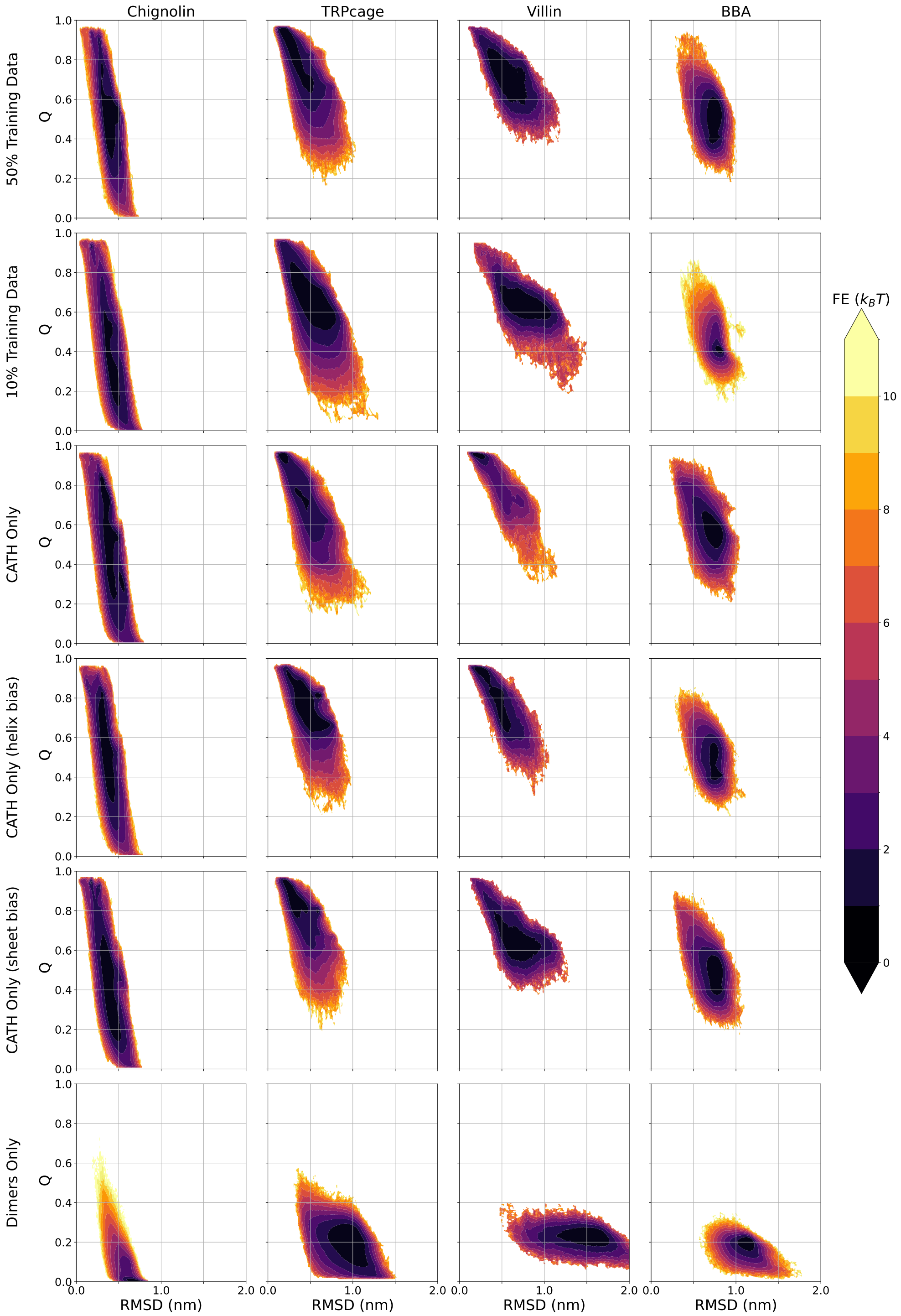

Fig. 20: Simulations of Chignolin, TRPcage, Villin, and BBA performed with various subsets of the training dataset. From top to bottom, these subsets are defined by: 50% of the total data, 10% of the total data, only the CATH proteins, only the 20 CATH proteins with the highest helical content, only the 20 CATH proteins with the highest sheet content, and only the dimer dataset.

6.6 Decoy study

The fraction of decoys with respect to the sampled structures, and the level of noise added to create the decoys were chosen empirically from a set of models trained with different amounts of decoys and noise levels. Apart from the noise level and amount of decoys, all models were trained with the same set of hyperparameters. The best performing noise level was chosen to be 0.5 Å and the amount of decoys chosen to be every 50th frame for each molecule in the training set. The results of models trained with a different noise level are shown in Supplementary Fig. 21a) and the models with different amounts of decoys are shown in Supplementary Fig. 21b). Note that the first row of Fig. 21b) shows the performance of a

model without decoys. The model producing a stable folded state for all fast-folders was chosen as the transferable model discussed in the main text.

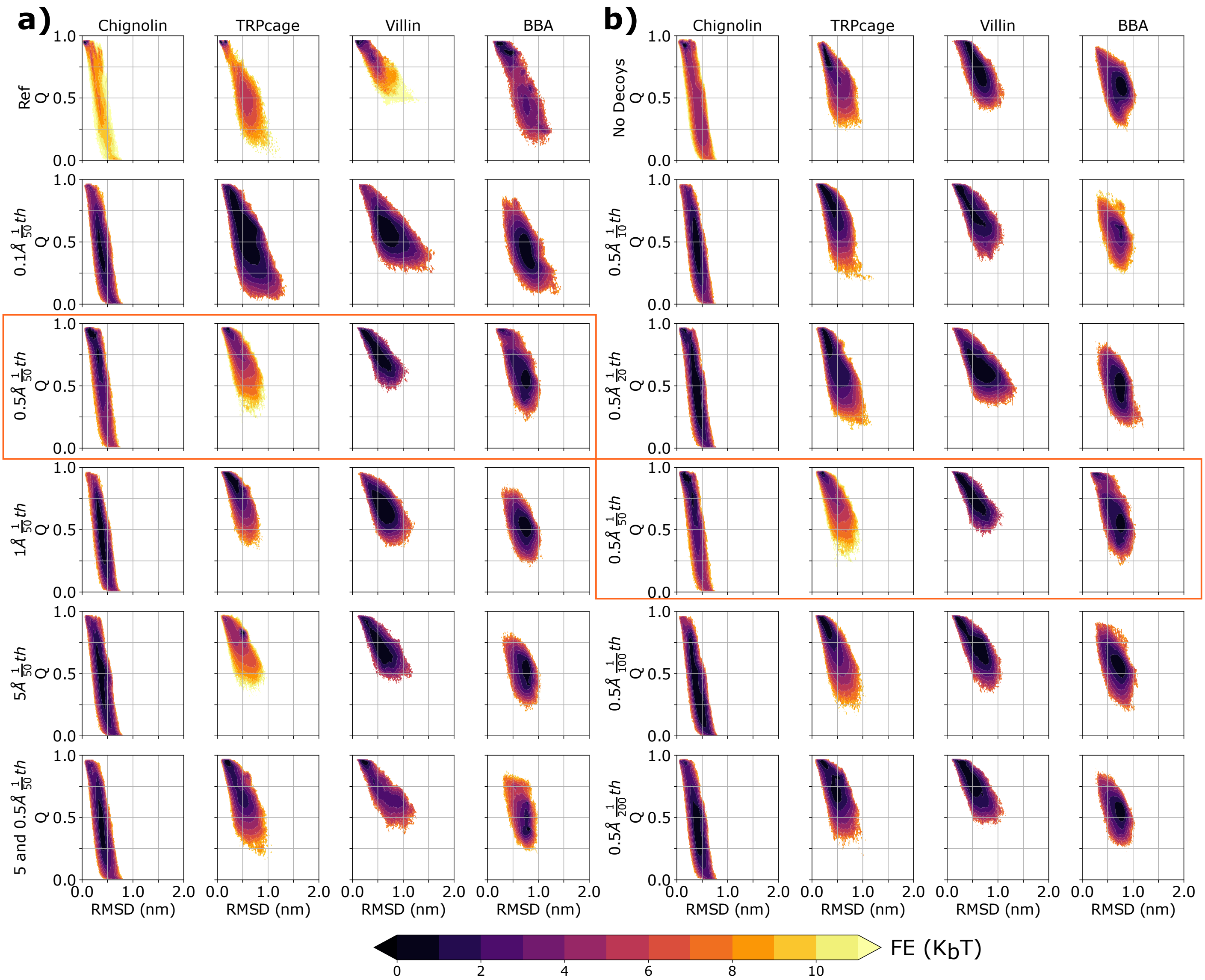

Fig. 21: Performance of models trained with different decoy noise levels (a) and different amount of decoys (b) on the four fast-folding proteins Chignolin (2RVD), TRPcage (2JOF), Villin (1YRF), and BBA (1FME). The transferable model discussed in the main text is highlighted in orange in both plots. The first row of (a) shows the reference all-atom (AMBER) simulations and the first row of (b) shows a model trained without decoys.

6.7 Timestep analysis

Issues with numerical stability during simulation of neural network force fields are well known [25, 61]. To assess the stability of our model at various simulation timesteps, we performed Langevin Dynamics simulations of the four smallest fast-folding proteins (Chignolin, TRPcage, BBA and Villin) with 2, 3, 4, 5, and 6 fs as integration timesteps. The simulations with a 6 fs timestep became numerically unstable after 10 steps for all the proteins. All the other simulations were stable in terms of energy conservation (Supplementary Fig. 23) and converged to a FES comparable to the 4 fs timestep simulations reported in the main text (Supplementary Fig. 22). The fact that the simulations become unstable for timesteps larger than 5 fs is not surprising, as our CG mapping preserves all the heavy atoms of the backbone as CG sites, along with the covalent bonds between those atoms; these physically-consistent bonds contribute a harmonic term to the prior energy function. The vibrational frequency of these physical bonds imposes an upper bound on the integration timestep: The timescale associated to the amide I vibrational stretch (1700-1600 cm^{-1}) is about 20 fs. The

common recommendation in MD simulations is to use a timestep about an order of magnitude smaller than the fastest time scale in the system, consistent with our analysis.

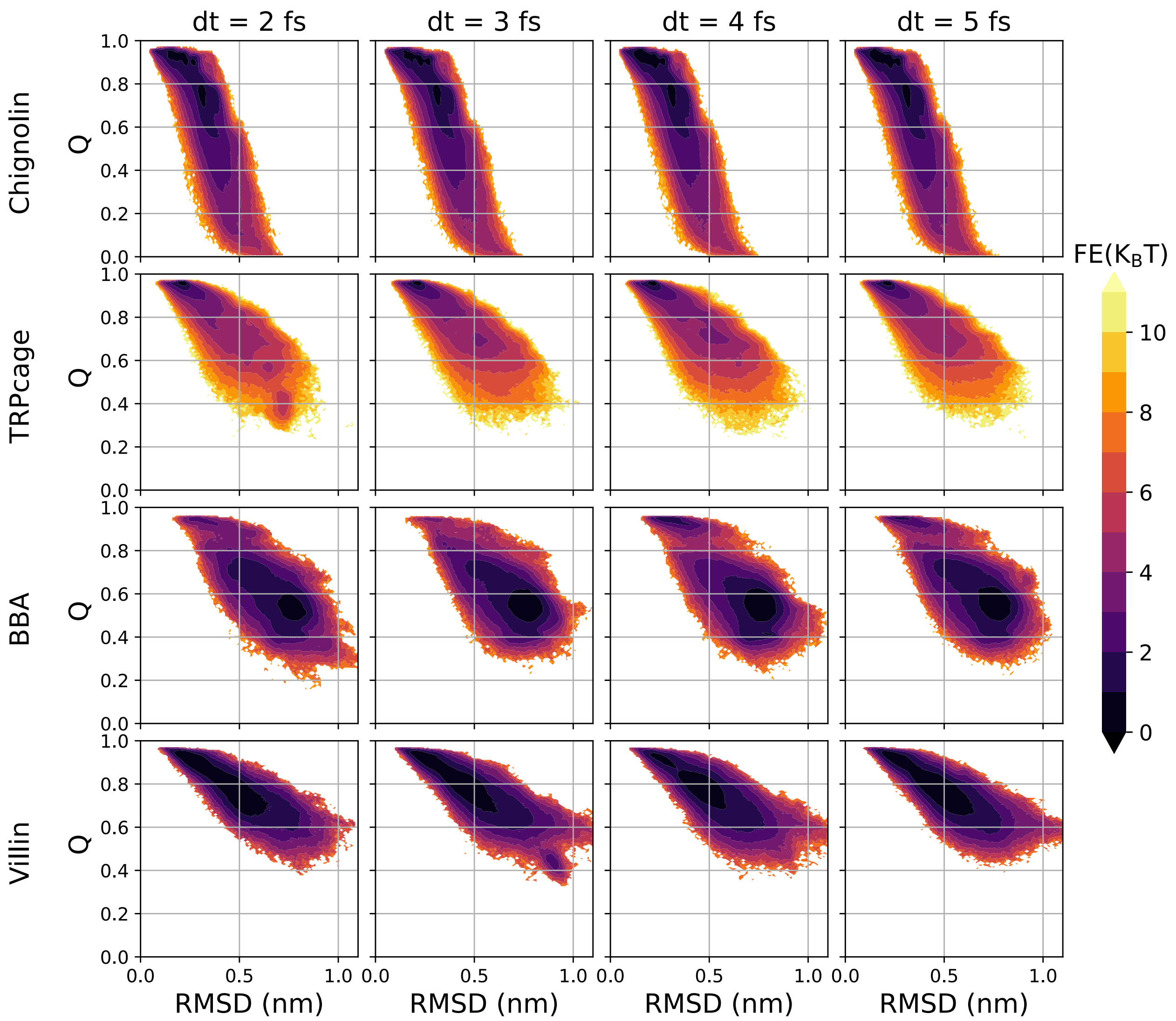

Fig. 22: Free energy surfaces for Langevin simulations with timesteps of 2, 3, 4, and 5 fs of the 4 fast-folding proteins. The initial frames corresponding to the first 2 CG-ns of simulation are discarded as equilibration.

6.8 Detailed analysis of alpha3D free energy landscape

For the protein A3D, the CG simulations from our transferable model visit predominantly two metastable states, both of which are visualized in Supplementary Fig. 24. The most populated metastable state corresponds quite closely to the folded state seen in reference DESRES simulations and the folded state predicted by AlphaFold3 (see Supplementary Fig. 12). The second, less populated, metastable state is characterized by a visually similar but fundamentally different structure, wherein the arrangement of the helix order is of the opposite sense (clockwise instead of counterclockwise, when viewing the bundle down the central axis from the perspective of the N terminus) and the position of the second helix is inverted. It is worth noting also that the UNRES CG simulations of 2A3D also stabilize this same misfolded state.

This misfolded state is not unprecendented. Before the *de novo* design of alpha3D[62], investigations on variations of its sequence folding into similar tri-helix bundle proteins also observed a similar alternative helical packing state [63, 64]. It was shown that the stability of different helical packing states was dominated primarily by inter-helical electrostatic interactions between GLU, LYS, and ARG residues, wherein (counter)clockwise packing with alternating residue charges placed at key positions in each helix promotes bundle stability [65].

Ten CG structures were extracted at random from this misfolded state and backmapped to atomistic resolution using ambertools [66] (version 23.6), specifically the command `pdb4amber`. The resulting atomistic were solvated in a

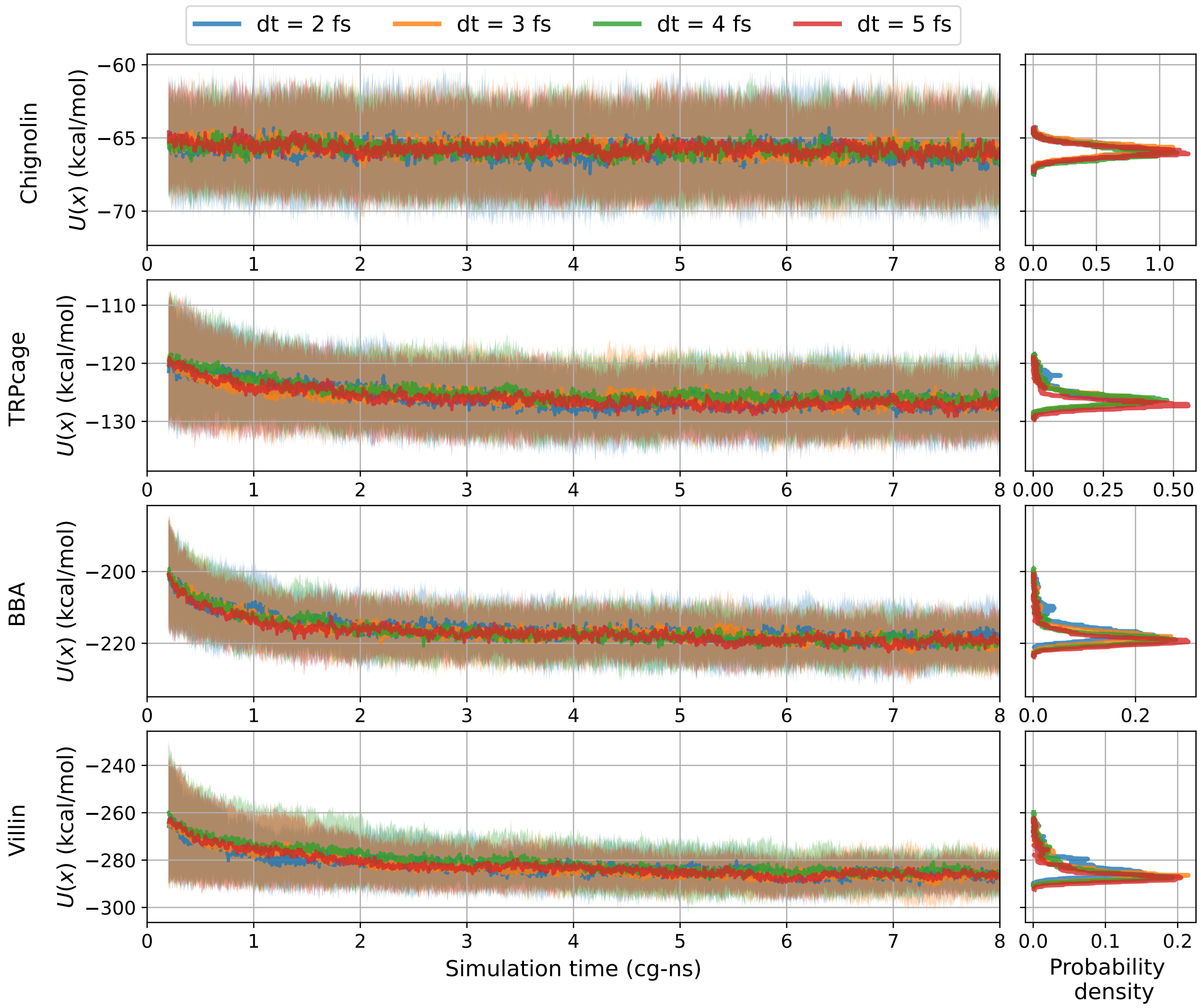

Fig. 23: (Left) Rolling mean of the potential energy as function of the simulation time; (Right) the distribution of the values for Langevin simulations with timesteps of 2,3,4, and 5 fs of the 4 smallest fast folding proteins. The initial frames corresponding to the first 2 CG-ns of simulation are discarded as equilibration.

dodecahedral box with a padding of 1.0 nm, at neutral pH and with 0.1 M ion concentration, using GROMACS [4] (version 2022.5), specifically the command `gmx solvate`. The solvated structures were energy minimized and then equilibrated at 300K in the NVT ensemble for 100 ps, using GROMACS. After equilibration, we performed 1 μs NPT simulations at 300K, with OpenMM and the AMBER ff-99SB-ILDN force field. Interestingly, this alternative geometry appears relatively stable in atomistic simulations as MD trajectories remain trapped in this metastable state for at least 1 μs.

This alternative state could be a remnant of the iterative design of this protein, or a spurious metastable state inherited from the reference force field.

6.9 Langevin trajectories and folding/unfolding events

In Fig. 3, folding trajectories for Homeodomain and Alpha3D are shown (from Langevin simulations at 300K). For the four fast-folding proteins, the free energy surfaces are shown as obtained from MBAR-reweighted PT simulations (Fig. 2) and from Langevin simulations (Supplementary Fig. 19). By inspecting the Langevin trajectories, multiple folding and unfolding events are observed for all these proteins, illustrating the good sampling of the folded states by the CG model. Examples of these trajectories are shown in Supplementary Fig. 25.

6.10 Simulation of training proteins

The main novelty of this work is the transferability of the model that can perform simulations on proteins completely outside its training set. It is nonetheless interesting to analyze the performance of the model on proteins inside the training set. To this end, we conduct PT simulations on four CATH domains from the training set, with the same parameters as

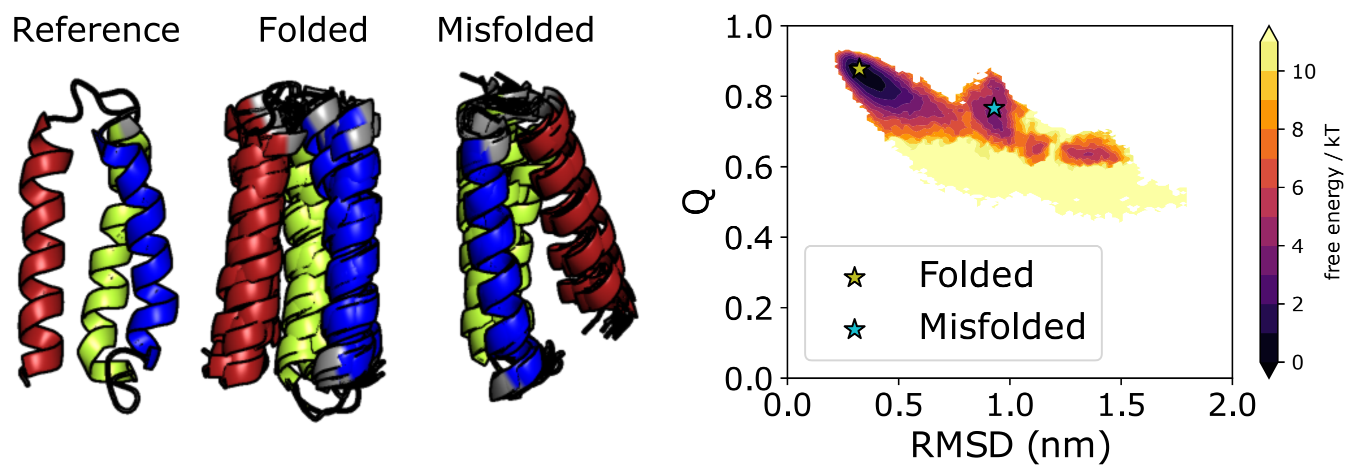

Fig. 24: Structures from the folded and misfolded basin from A3D simulations. The misfolded basin (that is also seen in the A3D UNRES simulations, see Supplementary Fig. 13) corresponds to a misfolded structure where the relative positions of the three helices are inverted compared to their positions in the folded and reference structures.

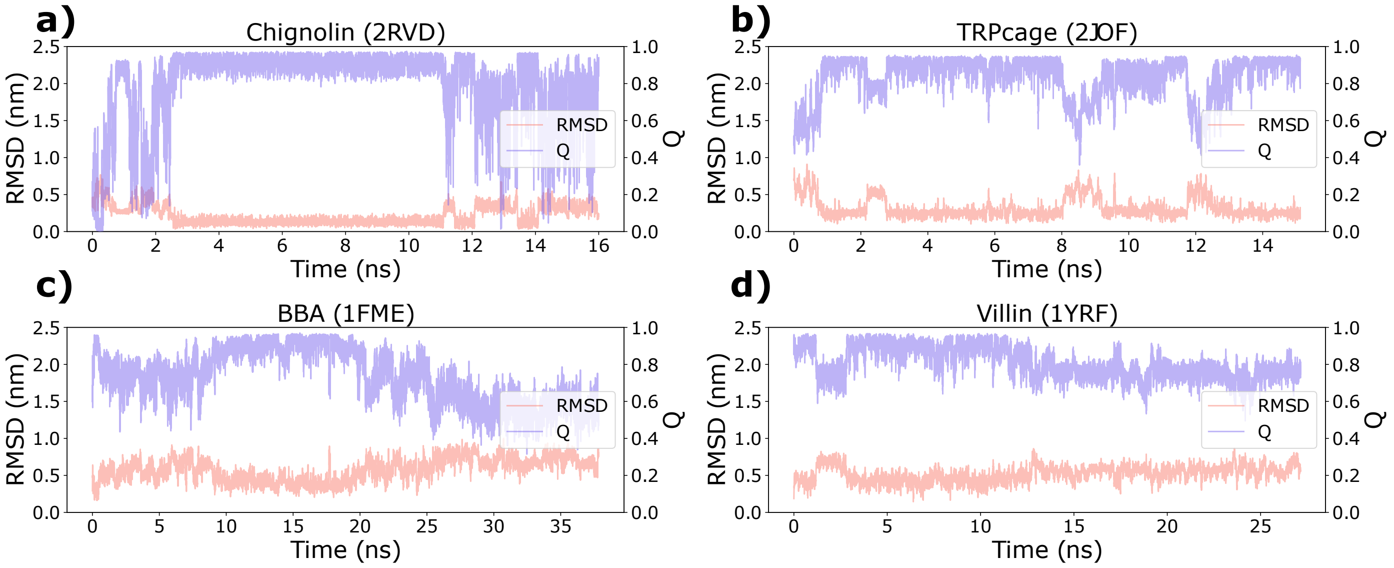

Fig. 25: Example Langevin trajectories obtained for the four fast-folding proteins discussed in the main text. Each of these trajectories exhibits folding as well as unfolding events.

SH3 and CI2 shown in Supplementary Table 6. The four CATH domains chosen are one mostly sheet-like domain (with a beta-barrel like structure), 1skyB01, one mostly helical domain, 2e8oA01, and two mixed alpha/beta structures, 3ethA03 (that also has a higher coil content) and 4o96A01. Supplementary Fig. 26 shows the free energy surface obtained through these simulations as well as structures from the labeled basins. The model is able to stabilize folded states close to the reference experimental structures of these domains and also explore other parts of the conformational space, sampling additional misfolded and unfolded states. 3ethA03 has a folded state with a slightly lower fraction of native contacts because of its high coil content, providing a higher flexibility to the protein during simulation. 4o96A01 also has a slightly lower Q in its folded state due to the position of the helix that tilts somewhat during simulation. The protein exhibiting a decreased stability of its folded state is 2e8oA01, the most helical-like protein, and the only one where the reference PDB structure is obtained by NMR instead of X-ray. The folded state stabilized by the CG model has a full helix on residues 62 to 74 instead of a smaller helix on the last 6 residues and a turn on residues 63-64 (see the right part of the orange and grey structures respectively). It is worth noting that for most of these domains, and especially 2e8oA01, the residual helix content in the sampled unfolded state is very high, likely due to the fact that only structures from the folded state of these proteins are present in the training set. Indeed, the all-atom simulations of these domains comprising the training dataset (as described in Supplementary Section 1.1) are too short to observe any large scale structural changes and only sample their folded state.

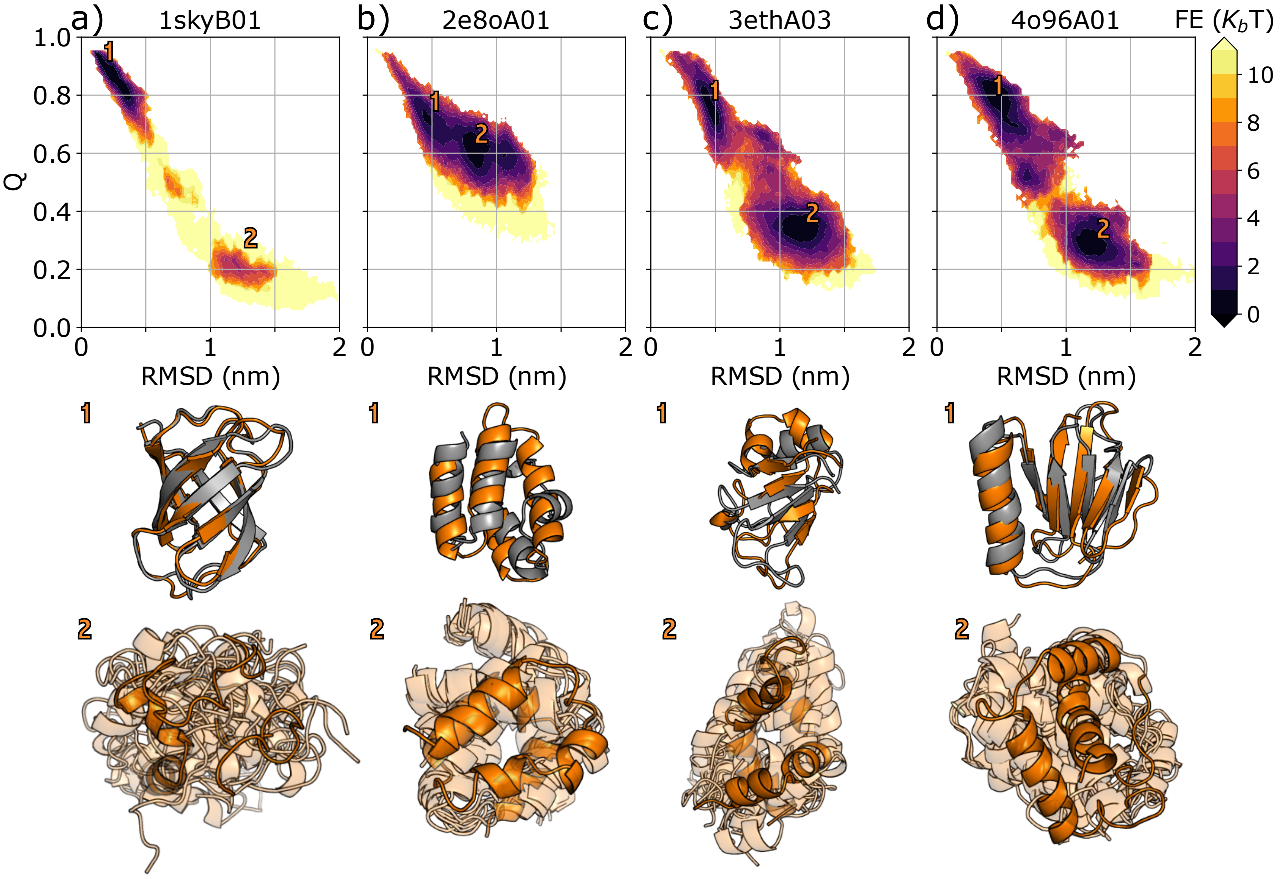

Fig. 26: Simulations of four CATH domains taken from the training set. Each column shows the exploration of the free energy landscape as obtained with MBAR-reweighted PT simulations of these CATH domains and structures from the metastable basins indicated with the labels in orange. The grey structures are reference experimental structures for these domains (NMR structure for 2e8oA01 and crystal structures for the others).

6.11 Martini large protein simulations

The Martini force fields [67] are known to need an elastic network to preserve contacts formed in a protein's folded state [42]. In contrast, our goal in designing a transferable CG model using a machine-learned force field is to explore the full free energy landscape of a protein system without the necessity of *a priori* knowledge of the system's most stable conformation. For this reason, all Martini simulations to which we compare our model's performance do not use an elastic network to bias the system to its folded state structure. To assess whether the addition of such a network would yield better performance for our test systems, we simulated the Homeodomain and A3D with Martini both with and without an elastic network model, as prescribed in [42]. The results are shown in Supplementary Fig. 27. Clearly, the simulations performed without an elastic network are not able to stabilize the folded state of the systems of interest, in contrast to our model. In addition, the use of a biasing network strongly prevents the system from exploring the entire conformational landscape of the proteins. Rather, the biasing network traps these systems in the folded state.

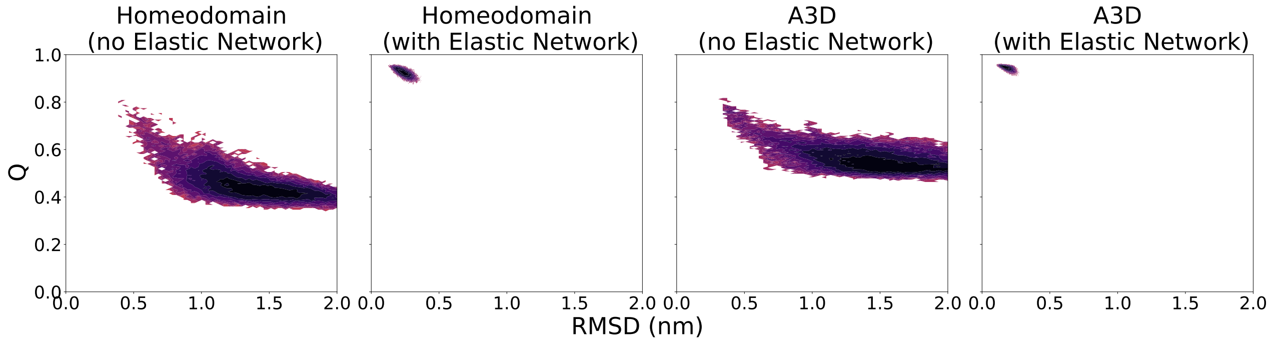

Fig. 27: Simulations of the two larger protein test systems, Homeodomain (1ENH) and alpha3D (2A3D) with the Martini force field with and without the incorporation of an elastic network model.

6.12 Contact maps

In addition to the free energy surfaces and structural metric comparisons provided in Supplementary Figs. 13 and 12, we also compute the C_α contact maps for all folded structures from all the CG models (as well as the all-atom DESRES simulations) discussed in the main text. We use the structures extracted for Supplementary Fig. 12 from the most folded-like metastable basin following the procedure described in Supplementary Section 5.4.3 and compute a contact map for each of them. We then average the contact maps to obtain a combined map where the darker the color, the more structures, on average, contain this contact. We then compare the contact map directly against the one corresponding to the PDB structure. We also compute the contact maps for the larger proteins presented in Supplementary Fig. 17, using the same method. The results are summarized in Supplementary Fig. 28 a and b respectively.

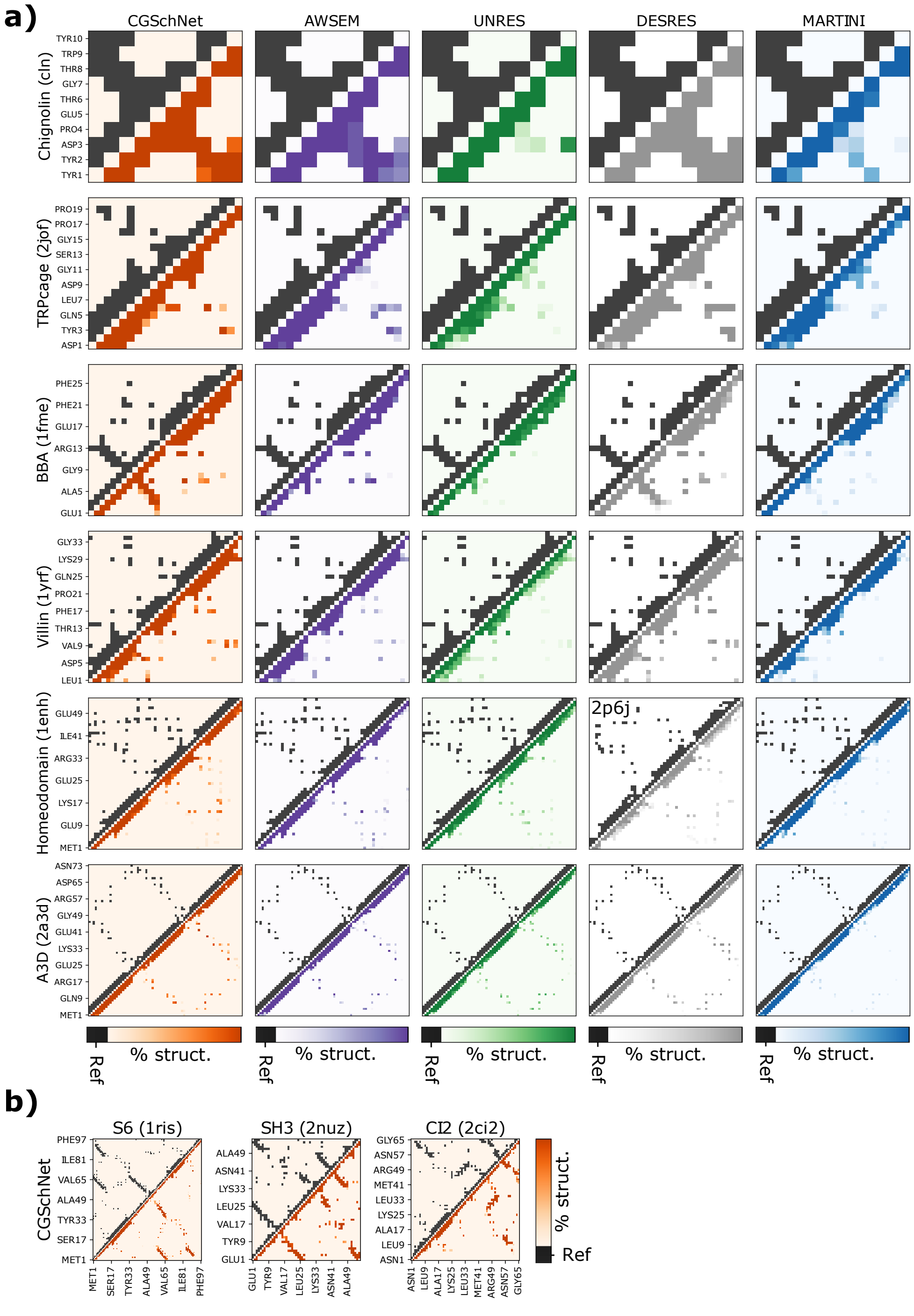

Fig. 28: Contact maps for the fast folding proteins presented in the main text. For each protein and each force field, 10 folded structures were extracted from the most folded-like metastable basin and compared to reference folded structures. In each contact map, the upper left region (dark gray) shows the contacts within the reference structure while the lower right region shows the contacts within the 10 structures extracted from simulations. The contacts are averaged over the 10 structures, thus a contact with a very light color is present in only a few of the extracted structures and a contact with a darker color is present in most of the extracted structures. a) shows a comparison between CGSchNet, other CG force fields and the DESRES simulations [17] and b) shows the contact maps for the larger proteins, whose free energy surfaces appear in Supplementary Fig. 17, simulated with CGSchNet.

References

[1] Sillitoe, I., Lewis, T.E., Cuff, A., Das, S., Ashford, P., Dawson, N.L., Furnham, N., Laskowski, R.A., Lee, D., Lees, J.G., Lehtinen, S., Studer, R.A., Thornton, J., Orengo, C.A.: Cath: comprehensive structural and functional annotations for genome sequences. Nucleic Acids Res. **43**(D1), 376–381 (2015)

[2] Kabsch, W., Sander, C.: Dictionary of protein secondary structure: pattern recognition of hydrogen-bonded and geometrical features. Biopolymers **22**(12), 2577–2637 (1983)

[3] McGibbon, R.T., Beauchamp, K.A., Harrigan, M.P., Klein, C., Swails, J.M., Hernández, C.X., Schwantes, C.R., Wang, L.-P., Lane, T.J., Pande, V.S.: Mdtraj: A modern open library for the analysis of molecular dynamics trajectories. Biophys. J. **109**(8), 1528–1532 (2015)

[4] Abraham, M.J., Murtola, T., Schulz, R., Páll, S., Smith, J.C., Hess, B., Lindahl, E.: Gromacs: High performance molecular simulations through multi-level parallelism from laptops to supercomputers. SoftwareX **1**, 19–25 (2015)

[5] Lindorff-Larsen, K., Piana, S., Palmo, K., Maragakis, P., Klepeis, J.L., Dror, R.O., Shaw, D.E.: Improved side-chain torsion potentials for the amber ff99sb protein force field. Proteins **78**(8), 1950–1958 (2010)

[6] Jorgensen, W.L., Chandrasekhar, J., Madura, J.D., Impey, R.W., Klein, M.L.: Comparison of simple potential functions for simulating liquid water. J. Chem. Phys. **79**(2), 926–935 (1983)

[7] Schrödinger, L.: The PyMOL Molecular Graphics System, Version 1.8 (2015)

[8] Eastman, P., Swails, J., Chodera, J.D., McGibbon, R.T., Zhao, Y., Beauchamp, K.A., Wang, L.-P., Simmonett, A.C., Harrigan, M.P., Stern, C.D., Wiewiora, R.P., Brooks, B.R., Pande, V.S.: Openmm 7: Rapid development of high performance algorithms for molecular dynamics. PLOS Comput. Biol. **13**(7), 1005659 (2017)

[9] Durumeric, A.E.P., Chen, Y., Noé, F., Clementi, C.: Learning data efficient coarse-grained molecular dynamics from forces and noise. arXiv preprint arXiv:2407.01286 (2024)

[10] Wang, G., Dunbrack, R.L.: Pisces: a protein sequence culling server. Bioinformatics **19**(12), 1589–1591 (2003)

[11] Zimmerman, J.M., Eliezer, N., Simha, R.: The characterization of amino acid sequences in proteins by statistical methods. J. Theor. Biol. **21**(2), 170–201 (1968)

[12] Harvey, M.J., Giupponi, G., Fabritiis, G.D.: Acemd: Accelerating biomolecular dynamics in the microsecond time scale. J. Chem. Theory Comput. **5**(6), 1632–1639 (2009)

[13] Doerr, S., De Fabritiis, G.: On-the-fly learning and sampling of ligand binding by high-throughput molecular simulations. J. Chem. Theory Comput. **10**(5), 2064–2069 (2014)

[14] Prinz, J.-H., Wu, H., Sarich, M., Keller, B., Senne, M., Held, M., Chodera, J.D., Schütte, C., Noé, F.: Markov models of molecular kinetics: Generation and validation. J. Chem. Phys. **134**(17), 174105 (2011)

[15] Husic, B.E., Pande, V.S.: Markov state models: From an art to a science. J. Am. Chem. Soc. **140**, 2386–2396 (2018)

[16] Pérez-Hernández, G., Paul, F., Giorgino, T., De Fabritiis, G., Noé, F.: Identification of slow molecular order parameters for markov model construction. J. Chem. Phys. **139**(1), 015102 (2013)

[17] Lindorff-Larsen, K., Piana, S., Dror, R.O., Shaw, D.E.: How fast-folding proteins fold. Science **334**(6055), 517–520 (2011)

[18] Majewski, M., Pérez, A., Thölke, P., Doerr, S., Charron, N.E., Giorgino, T., Husic, B.E., Clementi, C., Noé, F., De Fabritiis, G.: Machine learning coarse-grained potentials of protein thermodynamics. Nat. Commun. **14**(1), 5739 (2023)

[19] Hoffmann, M., Scherer, M., Hempel, T., Mardt, A., Silva, B., Husic, B.E., Klus, S., Wu, H., Kutz, N., Brunton, S.L., Noé, F.: Deeptime: a python library for machine learning dynamical models from time series data. Mach. Learn.: Sci. Technol. **3**, 015009 (2022)

[20] Schütt, K.T., Kessel, P., Gastegger, M., Nicoli, K.A., Tkatchenko, A., Müller, K.-R.: Schnetpack: A deep learning

toolbox for atomistic systems. J. Chem. Theory Comput. **15**(1), 448–455 (2019)

[21] Wang, J., Olsson, S., Wehmeyer, C., Pérez, A., Charron, N.E., Fabritiis, G., Noé, F., Clementi, C.: Machine learning of coarse-grained molecular dynamics force fields. ACS Cent. Sci. **5**(5), 755–767 (2019)

[22] Unke, O.T., Meuwly, M.: Physnet: A neural network for predicting energies, forces, dipole moments, and partial charges. J. Chem. Theory Comput. **15**(6), 3678–3693 (2019)

[23] Kingma, D.P., Ba, J.: Adam: A method for stochastic optimization. In: Bengio, Y., LeCun, Y. (eds.) ICLR 3rd International Conference on Learning Representations. Curran Associates Inc., ??? (2015)

[24] Falcon, W., The PyTorch Lightning team: PyTorch Lightning. https://github.com/Lightning-AI/lightning

[25] Fu, X., Wu, Z., Wang, W., Xie, T., Keten, S., Gomez-Bombarelli, R., Jaakkola, T.: Forces are not enough: Benchmark and critical evaluation for machine learning force fields with molecular simulations. arXiv preptint arXiv:2210.07237 (2022)

[26] Husic, B.E., Charron, N.E., Lemm, D., Wang, J., Pérez, A., Krämer, A., Chen, Y., Olsson, S., Fabritiis, G., Noé, F., Clementi, C.: Coarse graining molecular dynamics with graph neural networks. J. Chem. Phys. **153**(19), 194101 (2020)

[27] Chen, Y., Krämer, A., Charron, N.E., Husic, B.E., Clementi, C., Noé, F.: Machine learning implicit solvation for molecular dynamics. J. Chem. Phys. **155**(8), 084101 (2021)

[28] Virtanen, P., Gommers, R., Oliphant, T.E., Haberland, M., Reddy, T., Cournapeau, D., Burovski, E., Peterson, P., Weckesser, W., Bright, J., van der Walt, S.J., Brett, M., Wilson, J., Millman, K.J., Mayorov, N., Nelson, A.R.J., Jones, E., Kern, R., Larson, E., Carey, C.J., Polat, İ., Feng, Y., Moore, E.W., VanderPlas, J., Laxalde, D., Perktold, J., Cimrman, R., Henriksen, I., Quintero, E.A., Harris, C.R., Archibald, A.M., Ribeiro, A.H., Pedregosa, F., van Mulbregt, P., SciPy 1.0 Contributors: SciPy 1.0: Fundamental Algorithms for Scientific Computing in Python. Nat. Methods **17**, 261–272 (2020)

[29] Krämer, A., Durumeric, A.E.P., Charron, N.E., Chen, Y., Clementi, C., Noé, F.: Statistically optimal force aggregation for coarse-graining molecular dynamics. J. Phys. Chem. Lett. **14**(17), 3970–3979 (2023)

[30] Rathore, N., Chopra, M., Pablo, J.J.: Optimal allocation of replicas in parallel tempering simulations. J. Chem. Phys. **122**(2) (2005)

[31] Shirts, M.R., Chodera, J.D.: Statistically optimal analysis of samples from multiple equilibrium states. J. Chem. Phys. **129**(12), 124105 (2008)

[32] Scherer, M.K., Trendelkamp-Schroer, B., Paul, F., Pérez-Hernández, G., Hoffmann, M., Plattner, N., Wehmeyer, C., Prinz, J.-H., Noé, F.: PyEMMA 2: A Software Package for Estimation, Validation, and Analysis of Markov Models. J. Chem. Theory Comput. **11**, 5525–5542 (2015)

[33] Czaplewski, C., Karczynska, A., Sieradzan, A.K., Liwo, A.: Unres server for physics-based coarse-grained simulations and prediction of protein structure, dynamics and thermodynamics. Nucleic Acids Res. **46**(W1), 304–309 (2018)

[34] Slusarz, R., Lubecka, E.A., Czaplewski, C., Liwo, A.: Improvements and new functionalities of unres server for coarse-grained modeling of protein structure, dynamics, and interactions. Front. Mol. Biosci. **9** (2022)

[35] Liwo, A., Sieradzan, A.K., Lipska, A.G., Czaplewski, C., Joung, I., Zmudzinska, W., Halabis, A., Oldziej, S.: A general method for the derivation of the functional forms of the effective energy terms in coarse-grained energy functions of polymers. iii. determination of scale-consistent backbone-local and correlation potentials in the unres force field and force-field calibration and validation. J. Chem. Phys. **150**(15), 155104 (2019)

[36] Davtyan, A., Schafer, N.P., Zheng, W., Clementi, C., Wolynes, P.G., Papoian, G.A.: AWSEM-MD: protein structure prediction using coarse-grained physical potentials and bioinformatically based local structure biasing. J. Phys. Chem. B **116**(29), 8494–8503 (2012)

[37] Drozdetskiy, A., Cole, C., Procter, J., Barton, G.J.: JPred4: a protein secondary structure prediction server. Nucleic Acids Res. **43**(W1), 389–394 (2015)

[38] Jo, S., Kim, T., Iyer, V.G., Im, W.: Charmm-gui: A web-based graphical user interface for charmm. J. Comput. Chem. **29**(11), 1859–1865 (2008)

[39] Qi, Y., Ingólfsson, H.I., Cheng, X., Lee, J., Marrink, S.J., Im, W.: Charmm-gui martini maker for modeling and simulation of complex biomolecular systems with martini 3. J. Chem. Inf. Model. **62**(3), 646–658 (2022)

[40] Bussi, G., Donadio, D., Parrinello, M.: Canonical sampling through velocity rescaling. J. Chem. Phys. **126**(1), 014101 (2007)

[41] Parrinello, M., Rahman, A.: Polymorphic transitions in single crystals: A new molecular dynamics method. J. Appl. Phys. **52**(12), 7182–7190 (1981)

[42] Periole, X., Cavalli, M., Marrink, S.-J., Ceruso, M.A.: Combining an elastic network with a coarse-grained molecular force field: Structure, dynamics, and intermolecular recognition. J. Chem. Theory Comput. **5**(9), 2531–2543 (2009)

[43] Wassenaar, T.A., Pluhackova, K., Böckmann, R.A., Marrink, S.J., Tieleman, D.P.: Going backward: A flexible geometric approach to reverse transformation from coarse grained to atomistic models. J. Chem. Theory Comput. **10**(2), 676–690 (2014)

[44] Best, R.B., Hummer, G., Eaton, W.A.: Native contacts determine protein folding mechanisms in atomistic simulations. Proc. Natl. Acad. Sci. U.S.A. **110**(44), 17874–17879 (2013)

[45] Poma, A.B., Cieplak, M., Theodorakis, P.E.: Combining the martini and structure-based coarse-grained approaches for the molecular dynamics studies of conformational transitions in proteins. J. Chem. Theory Comput. **13**(3), 1366–1374 (2017)

[46] Cock, P.J., Antao, T., Chang, J.T., Chapman, B.A., Cox, C.J., Dalke, A., Friedberg, I., Hamelryck, T., Kauff, F., Wilczynski, B., *et al.*: Biopython: freely available python tools for computational molecular biology and bioinformatics. Bioinformatics **25**(11), 1422–1423 (2009)

[47] Zemla, A., Venclovas, Č., Moult, J., Fidelis, K.: Processing and analysis of casp3 protein structure predictions. Proteins **37**(S3), 22–29 (1999)

[48] Abramson, J., Adler, J., Dunger, J., Evans, R., Green, T., Pritzel, A., Ronneberger, O., Willmore, L., Ballard, A.J., Bambrick, J., Bodenstein, S.W., Evans, D.A., Hung, C.-C., O'Neill, M., Reiman, D., Tunyasuvunakool, K., Wu, Z., Žemgulytė, A., Arvaniti, E., Beattie, C., Bertolli, O., Bridgland, A., Cherepanov, A., Congreve, M., Cowen-Rivers, A.I., Cowie, A., Figurnov, M., Fuchs, F.B., Gladman, H., Jain, R., Khan, Y.A., Low, C.M.R., Perlin, K., Potapenko, A., Savy, P., Singh, S., Stecula, A., Thillaisundaram, A., Tong, C., Yakneen, S., Zhong, E.D., Zielinski, M., Žídek, A., Bapst, V., Kohli, P., Jaderberg, M., Hassabis, D., Jumper, J.M.: Accurate structure prediction of biomolecular interactions with AlphaFold 3. Nature **630**(8016), 493–500 (2024)

[49] Shah, P.S., Hom, G.K., Ross, S.A., Lassila, J.K., Crowhurst, K.A., Mayo, S.L.: Full-sequence computational design and solution structure of a thermostable protein variant. J. Mol. Biol. **372**(1), 1–6 (2007)

[50] Zemla, A.: Lga: a method for finding 3d similarities in protein structures. Nucleic Acids Res. **31**(13), 3370–3374 (2003)

[51] Zhang, Y., Skolnick, J.: Scoring function for automated assessment of protein structure template quality. Proteins **57**(4), 702–710 (2004)

[52] Savitzky, A., Golay, M.J.E.: Smoothing and differentiation of data by simplified least squares procedures. Anal. Chem. **36**(8), 1627–1639 (1964)

[53] Day, C.L., Smits, C., Fan, F.C., Lee, E.F., Fairlie, W.D., Hinds, M.G.: Structure of the bh3 domains from the p53-inducible bh3-only proteins noxa and puma in complex with mcl-1. J. Mol. Biol. **380**(5), 958–971 (2008)

[54] Cornilescu, G., Marquardt, J.L., Ottiger, M., Bax, A.: Validation of protein structure from anisotropic carbonyl chemical shifts in a dilute liquid crystalline phase. J. Am. Chem. Soc. **120**(27), 6836–6837 (1998)

[55] Matysiak, S., Clementi, C.: Optimal combination of theory and experiment for the characterization of the protein folding landscape of s6: How far can a minimalist model go? J. Mol. Biol. **343**, 235–248 (2004)

[56] Matysiak, S., Clementi, C.: Minimalist protein model as a diagnostic tool for misfolding and aggregation. J. Mol. Biol. **363**(1), 297–308 (2006)

[57] Went, H.M., Jackson, S.E.: Ubiquitin folds through a highly polarized transition state. Prot. Eng. Des. Sel. **18**(5), 229–237 (2005)

[58] Aldeghi, M., Groot, B.L., Gapsys, V.: Accurate calculation of free energy changes upon amino acid mutation. In: Sikosek, T. (ed.) Computational Methods in Protein Evolution, pp. 19–47. Springer, New York, New York, United States (2019)

[59] Eastman, P., Galvelis, R., Peláez, R.P., Abreu, C.R.A., Farr, S.E., Gallicchio, E., Gorenko, A., Henry, M.M., Hu, F., Huang, J., Krämer, A., Michel, J., Mitchell, J.A., Pande, V.S., Rodrigues, J.P., Rodriguez-Guerra, J., Simmonett, A.C., Singh, S., Swails, J., Turner, P., Wang, Y., Zhang, I., Chodera, J.D., De Fabritiis, G., Markland, T.E.: Openmm 8: Molecular dynamics simulation with machine learning potentials. J. Phys. Chem. B **128**(1), 109–116 (2023)

[60] Noé, F., Clementi, C.: Collective variables for the study of long-time kinetics from molecular trajectories: theory and methods. Curr. Opin. Struct. Biol. **43**, 141–147 (2017)

[61] Stocker, S., Gasteiger, J., Becker, F., Günnemann, S., Margraf, J.T.: How robust are modern graph neural network potentials in long and hot molecular dynamics simulations? Mach. learn.: sci. technol. **3**(4), 045010 (2022)

[62] Walsh, S.T.R., Cheng, H., Bryson, J.W., Roder, H., DeGrado, W.F.: Solution structure and dynamics of a de novo designed three-helix bundle protein. Proc. Natl. Acad. Sci. U.S.A. **96**(10), 5486–5491 (1999)

[63] O'Neil, K.T., DeGrado, W.F.: A thermodynamic scale for the helix-forming tendencies of the commonly occurring amino acids. Science **250**(4981), 646–651 (1990)

[64] Lovejoy, B., Choe, S., Cascio, D., McRorie, D.K., DeGrado, W.F., Eisenberg, D.: Crystal structure of a synthetic triple-stranded α-helical bundle. Science **259**(5099), 1288–1293 (1993)

[65] Bryson, J.W., Desjarlais, J.R., Handel, T.M., Degrado, W.F.: From coiled coils to small globular proteins: Design of a native-like three-helix bundle. Prot. Sci. **7**(6), 1404–1414 (1998)

[66] Case, D.A., Aktulga, H.M., Belfon, K., Cerutti, D.S., Cisneros, G.A., Cruzeiro, V.W.D., Forouzesh, N., Giese, T.J., Götz, A.W., Gohlke, H., Izadi, S., Kasavajhala, K., Kaymak, M.C., King, E., Kurtzman, T., Lee, T.-S., Li, P., Liu, J., Luchko, T., Luo, R., Manathunga, M., Machado, M.R., Nguyen, H.M., O'Hearn, K.A., Onufriev, A.V., Pan, F., Pantano, S., Qi, R., Rahnamoun, A., Risheh, A., Schott-Verdugo, S., Shajan, A., Swails, J., Wang, J., Wei, H., Wu, X., Wu, Y., Zhang, S., Zhao, S., Zhu, Q., Cheatham, T.E.I., Roe, D.R., Roitberg, A., Simmerling, C., York, D.M., Nagan, M.C., Merz, K.M.J.: AmberTools. J. Chem. Inf. Model. **63**(20), 6183–6191 (2023)

[67] Souza, P.C.T., Alessandri, R., Barnoud, J., Thallmair, S., Faustino, I., Grünewald, F., Patmanidis, I., Abdizadeh, H., Bruininks, B.M.H., Wassenaar, T.A., Kroon, P.C., Melcr, J., Nieto, V., Corradi, V., Khan, H.M., Domański, J., Javanainen, M., Martinez-Seara, H., Reuter, N., Best, R.B., Vattulainen, I., Monticelli, L., Periole, X., Tieleman, D.P., Vries, A.H., Marrink, S.J.: Martini 3: a general purpose force field for coarse-grained molecular dynamics. Nat. Methods **18**(44), 382–388 (2021)
